# Molecular and System-Level Characterization of MMP12 Suppression in Lung Cancer: A Combined Bioinformatics and Molecular Approach

**DOI:** 10.3390/ijms262411802

**Published:** 2025-12-06

**Authors:** Shriefa Almutairi, Rima Hajjo, Dima A. Sabbah, Kamal Sweidan, Zainab Ahmed Rashid, Sanaa K. Bardaweel

**Affiliations:** 1 Department of Pharmaceutical Sciences, School of Pharmacy, The University of Jordan, Amman 11942, Jordan; 2 Department of Pharmacy, Faculty of Pharmacy, Al-Zaytoonah University of Jordan, Amman 11733, Jordan; r.hajjo@zuj.edu.jo (R.H.); dima.sabbah@zuj.edu.jo (D.A.S.); 3 Laboratory for Molecular Modeling, Division of Chemical Biology and Medicinal Chemistry, Eshelman School of Pharmacy, The University of North Carolina at Chapel Hill, Chapel Hill, NC 27599, USA; 4 Department of Chemistry, The University of Jordan, Amman 11942, Jordan; k.sweidan@ju.edu.jo

**Keywords:** apoptosis, bioinformatics, cell cycle arrest, computational chemical biology, lung cancer, metastasis, migration, MMP12 inhibition, signaling pathways

## Abstract

Lung cancer remains a major cause of cancer-related death, highlighting the need for new molecular targets and novel therapeutics. Matrix metalloproteinases are key regulators of invasion and microenvironment remodeling, and among them, matrix metalloproteinase-12 (MMP12) is a particularly attractive candidate whose network-level effects in cancer are still poorly defined. Herein, we applied an integrative strategy that combines bioinformatics methods with experimental validation in non-small cell lung cancer (NSCLC) cells. Protein–protein interaction (PPI) and pathway analyses of MMP12-regulated genes identified 113 downstream targets enriched in the extracellular matrix, PI3K–AKT, and immune pathways, from which an eight-gene panel (MMP12, CD44, ADAM9, NFKBIA, PSME3, SPARCL1, CCL15, and APOA1) was prioritized as a biomarker signature. Guided by these predictions, we screened a 31-compound MMP12 inhibitor library and selected five leads (**C1**, **C7**, **C9**, **C10**, and **C15**) for testing in H1299 cells, with **C9** showing the strongest antiproliferative activity. These compounds showed antimigratory activity (**C1** achieving a 90% inhibition of wound closure at its IC_50_ concentration), reduced clonogenic growth, cell cycle perturbation, and induction of apoptosis. Gene- and protein-expression analyses confirmed MMP12 suppression and modulation of the eight-gene panel. Upstream regulator predictions implicated reduced AKT signaling alongside an ADAM9-centered adaptive axis. Collectively, these findings highlight **C1**, **C7**, **C9**, **C10**, and **C15** as promising MMP12 inhibitors, supporting their further development in preclinical lung cancer and nominating the eight-gene panel as a pharmacodynamic signature for MMP12-targeted therapies.

## 1. Introduction

Matrix metalloproteinases (MMPs) are a family of zinc- and calcium-dependent endopeptidases that play crucial roles in the degradation of extracellular matrix (ECM) components [1]. They are involved in various biological processes, including tissue remodeling, inflammation, wound healing, and cancer progression [2,3,4,5,6,7,8,9,10,11]. In cancer biology, MMPs are particularly recognized for facilitating tumor invasion, metastasis, and angiogenesis by remodeling the tumor microenvironment [3,5,12,13,14,15]. Among the 23 known human MMPs, several have been investigated as potential drug targets; however, the translation into effective therapies has faced challenges, such as a lack of specificity and dose-limiting toxicity [12,16,17].

MMP12, also known as macrophage metalloelastase, has garnered significant attention for its role in both physiological and pathological processes [2]. It is primarily secreted by macrophages and is involved in the degradation of elastin and other ECM proteins [18]. Beyond its traditional association with pulmonary diseases like chronic obstructive pulmonary disease (COPD), MMP12 has increasingly been recognized for its role in cancer biology [19,20,21]. Research has linked MMP12 to tumor cell invasion, angiogenesis, and metastasis by breaking down physical barriers within the tumor microenvironment [20,22]. It is often overexpressed in various human cancers, including lung adenocarcinoma and squamous cell carcinoma, where its expression is associated with increased invasiveness, enhanced migratory capacity, angiogenesis, and poor clinical outcomes [20,23,24,25,26,27].

Additionally, MMP12 modulates immune responses by regulating cytokines and chemokines, thereby promoting a tumor-supportive inflammatory environment [19,28]. Notably, MMP12 is expressed not only by tumor cells but also by tumor-associated macrophages, contributing to the dynamic interaction between the tumor and its microenvironment [29]. These findings highlight MMP12 as a promising yet underexplored target for therapeutic intervention in lung cancer. Given the strong involvement of MMP12 in tumor invasion, inflammation, and ECM remodeling, its activity is particularly relevant in lung cancer biology. Lung cancer is primarily divided into two main types: non-small cell lung cancer (NSCLC) and small cell lung cancer (SCLC) [30,31]. NSCLC accounts for nearly 85% of all lung cancer cases [32]. This type typically progresses more slowly than SCLC, but it is often diagnosed at advanced or metastatic stages, which results in a poor prognosis and limited treatment options [33].

NSCLC often exhibits significant ECM remodeling, increased inflammatory signaling, and unregulated protease activity [34,35,36]. This underscores the role of matrix metalloproteinases, such as MMP12, in lung cancer development. In contrast, SCLC is characterized by neuroendocrine differentiation, a more rapid clinical progression, and distinct biological drivers [37]. While interactions with the ECM may still play a role in SCLC progression, they are less emphasized in its typical pathobiology. As a result, targeting MMP12 is particularly relevant in NSCLC, making it the most suitable model for investigating its role in lung cancer progression and metastasis. These features align closely with the known functions of MMP12, underscoring its relevance as a potential driver of NSCLC progression.

Despite this compelling profile, the development of effective MMP12 inhibitors has been constrained due to the high structural homology among MMP family members, particularly within the catalytic domain [38,39]. Critically, most existing studies often evaluate MMP12 inhibition solely at the enzymatic level, overlooking its broader cellular impacts and the system-level mechanisms by which it influences cancer progression. We have addressed this gap through an integrative strategy that combines computational network biology with experimental validation. Building on a previous study on recently validated MMP12 inhibitors [40], this study aims to characterize the anticancer potential of selected MMP12 inhibitor candidates, identify associated genomic biomarkers, and define the mechanistic consequences of MMP12 suppression in lung cancer. We first used bioinformatics to map MMP12-associated pathways and regulatory networks. These in silico predictions then guided a two-stage experimental validation in non-small cell lung cancer (NSCLC) cells, starting with gene regulation and culminating in phenotypic screens of viability, migration, clonogenicity, apoptosis, and cell cycle progression.

The central hypothesis of this study is that targeted inhibition of MMP12 disrupts key oncogenic networks, as measured by specific genomic biomarkers, leading to suppressed lung cancer cell growth and metastatic potential. By integrating network-based discovery with molecular and cellular validation, this work provides a comprehensive framework for evaluating MMP12 as a therapeutic target and advances the rational development of specific inhibitors and MMP12 inhibition biomarkers.

## 2. Results

### 2.1. Systems Biology Analysis

#### 2.1.1. System Biology Informatics Workflow

**Figure 1:**
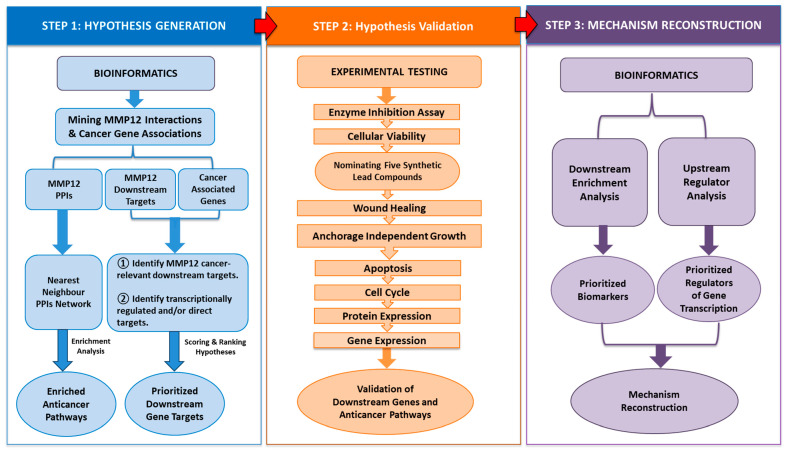
Integrative informatics workflow for investigating network biology of MMP12. Data mining and prioritization were followed by in vitro validation and computational pathway analysis to reconstruct potential mechanisms underlying MMP12 inhibition. PPIs refer to protein–protein interactions.

The adopted systems biology approach facilitated the integration of bioinformatics analyses with experimental validation. The overall workflow for identifying and analyzing biomarkers of MMP12 in lung cancer is illustrated in Figure 1. This workflow is based on the methods developed by Hajjo et al. [41,42,43] and has been tweaked to suit the available data for MMP12.

#### 2.1.2. Mining MMP12 Interactions

To delineate the functional landscape of MMP12 and better understand its anticancer potential in NCSLC, we performed an interaction analysis using the MetaCore^TM^ platform. This study specifically focused on downstream interactions, as they represent the direct functional consequences of MMP12 proteolytic activity and provide critical insight into the biological pathways modulated by its inhibition. A query for “MMP12” restricted to outgoing interactions identified 113 downstream genes functionally regulated by MMP12. This curated list (Table 1) formed the basis for subsequent analyses to categorize the regulatory mechanisms employed by MMP12. Critically, this integrative informatics workflow helped define the key experimental endpoints: (i) direct MMP12 enzyme inhibition; (ii) phenotypic readouts linked to predicted pathways (proliferation, migration, anchorage-independent growth, cell cycle distribution, and apoptosis); and (iii) modulation of a seven-gene panel (CD44, NFKBIA, ADAM9, PSME3, SPARCL1, CCL15, and APOA1) as a pharmacodynamic signature of MMP12 blockade.

**Figure 2:**
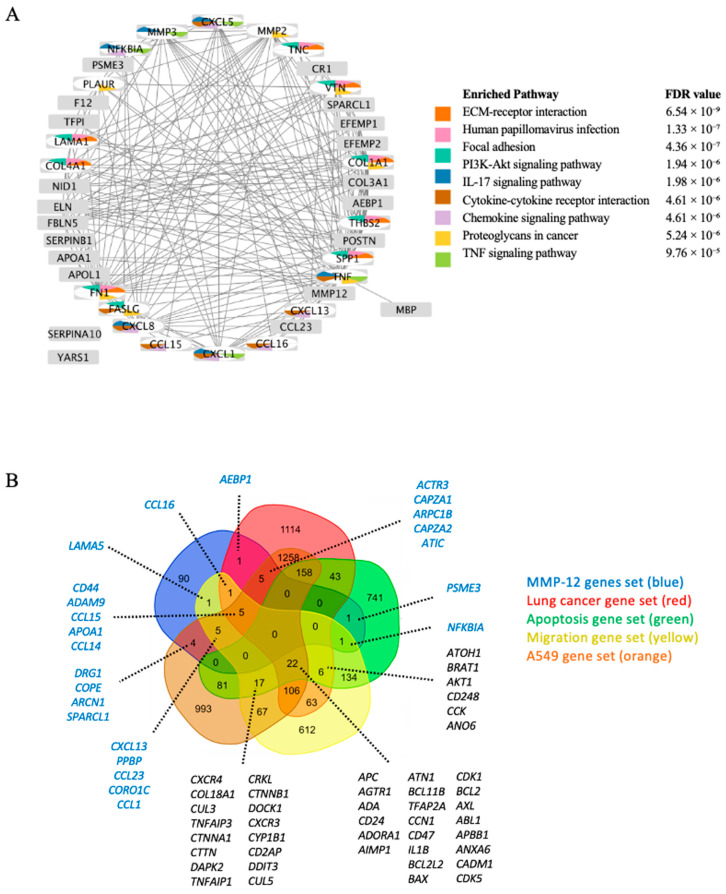
Analysis of MMP12 downstream targets and their overlap with cancer-associated gene sets: (**A**) Direct interaction network of MMP12 and its downstream targets. Nodes are color-coded according to the top 10 enriched KEGG pathways shown on the right. (**B**) Five-set Venn diagram showing the overlap between MMP12 downstream genes (blue) and four cancer-related MetaCore^TM^ datasets: lung cancer pathogenesis (red), apoptosis regulation (green), cell migration (yellow), and expressed in the A549 cell line (orange). Blue genes lists represent MMP12 outgoing genes, even if they also appear in other gene lists. Black genes represent genes that appear in at least one cancer-related gene list.

Further analysis revealed that while most downstream targets are modulated by MMP12 via proteolytic cleavage, three genes (*NFKBIA*, *PSME3*, and *SPARCL1*) are uniquely regulated at the transcriptional level. This suggests a non-canonical, indirect regulatory role for MMP12 beyond its primary proteolytic function. The connectivity landscape of MMP12 and its downstream targets highlighted its convergence on major cancer signaling axes, including ECM remodeling, PI3K-AKT, and cytokine signaling (Figure 2A). Critically, these enriched pathways directly anticipated the phenotypic outcomes to design the biological experiments: pathways like ECM–receptor interaction and focal adhesion aligned with suppressed migration and clonogenicity; PI3K-AKT and TNF signaling underpinned reduced proliferation and increased apoptosis; and cytokine and cell cycle networks corresponded to altered cell cycle distribution.

#### 2.1.3. Identification of Cancer-Associated and MMP12 Overlapping Genes

To examine the systems biology of MMP12 in lung cancer pathogenesis, we first established a framework of disease-relevant genes using the MetaCore^TM^ database. We systematically curated four gene sets associated with key oncogenic processes: lung cancer pathogenesis (2785 genes), apoptosis regulation (1204 genes), cell migration (1040 genes), and genes expressed in the A549 cell line (2721 genes). Subsequently, we performed a comparative overlap analysis to identify candidates linking MMP12 signaling to these processes. A five-set Venn diagram was generated to map the intersections between the 113 downstream targets of MMP12 and the four cancer-linked gene sets (Figure 2B). This analysis identified a core set of 24 genes that are downstream of MMP12 and implicated in at least one cancer-related category. These overlapping genes represent high-priority candidates, suggesting a functional convergence between MMP12 activity and established pathways in lung cancer development, apoptosis, and migration.

#### 2.1.4. Prioritization of MMP12-Responsive Genes for Experimental Validation

To identify the most promising candidates for downstream validation, we developed a prioritization scoring system based on three key criteria: (1) evidence of a direct interaction with MMP12, (2) annotation of transcriptional regulation by MMP12, and (3) overlap with cancer-associated gene sets (lung cancer, apoptosis, migration, expressed in A549). Genes received one point for each direct or transcriptional effect, and 1–3 points for overlapping with 1, 2, or 3 cancer gene sets (lung cancer, apoptosis, migration, expressed in A549), respectively, yielding a cumulative score from 0 to 5 (Table 2). Application of this scoring system identified *NFKBIA* and *CD44* as the top-ranked candidates, each with a score of 4, followed by a group of six genes (*ADAM9*, *APOA1*, *CCL15*, *PSME3*, and *SPARCL1*), which achieved a score of 3. Several other genes, including *CCL16* and *CXCL13*, scored 2, while *MMP2* and *TNF* scored 1 (Table 2).

For subsequent qPCR analysis, we selected a final panel of seven high-priority genes with scores ≥ 3: *NFKBIA*, *CD44*, *ADAM9*, *APOA1*, *CCL15*, *PSME3*, and *SPARCL1*. This selection was further guided by literature support and commercial assay availability. Based on MetaCore^TM^ predictions, *NFKBIA* and *CCL15* are activated by *MMP12*, whereas *PSME3*, *SPARCL1*, and *APOA1* are inhibited. The regulatory effects on *CD44* and *ADAM9* were unspecified.

#### 2.1.5. Pathway Enrichment of the Prioritized Genes

Pathway enrichment of the eight prioritized genes placed the MMP12 network in ECM remodeling, immune/inflammatory regulation, and cancer signaling (Table 3); each gene mapped to ≥1 significant pathway. Top hit “HOTAIR Regulatory Pathway” (FDR = 1.68 × 10^−3^; *CD44*, *MMP12*, *NFKBIA*), implicates epigenetic/EMT control. ECM programs were prominent—Collagen Degradation (FDR = 6.92 × 10^−3^; *MMP12*, *ADAM9*) and Extracellular Matrix Degradation (FDR = 8.35 × 10^−3^; *CD44, MMP12*). Immune pathways were also enriched: Role of Osteoclasts in Rheumatoid Arthritis (FDR = 5.88 × 10^−3^; *MMP12*, *ADAM9*, *NFKBIA*), T-cell receptor and Interleukin-1 signaling (both FDR = 8.83 × 10^−3^; *NFKBIA*, *PSME3*). Additional signals, such as post-translational phosphorylation and regulation of IGF transport/uptake by IGFBPs (both FDR = 8.83 × 10^−3^; *APOA1*, *SPARCL1*), suggest growth-factor control, while centrosomal KIAA0586 (FDR = 8.83 × 10^−3^; *CD44*, *PSME3*) points to polarity/immune-synapse roles.

**Figure 3:**
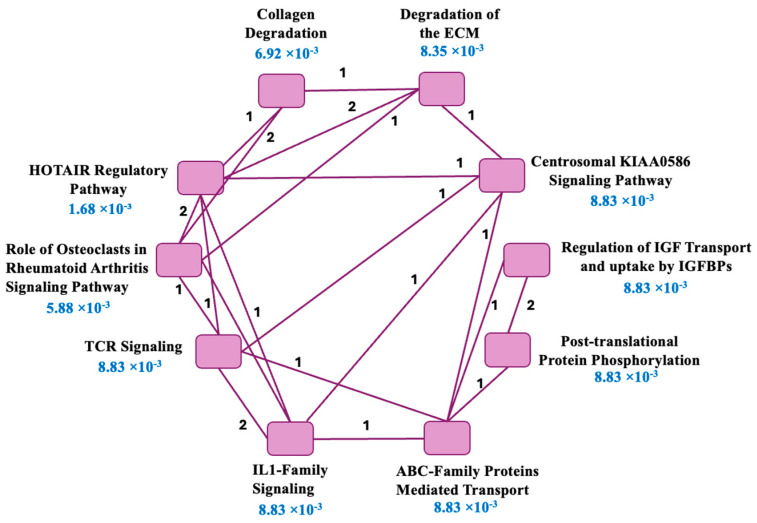
Pathway connectivity map for the eight MMP12-responsive genes prioritized for validation. Ingenuity pathway analysis (IPA) was used to identify the top 10 enriched canonical pathways linked to the prioritized downstream genes, and the network was redrawn for clarity. Purple nodes denote significantly enriched pathways; purple connecting edges indicate pathway pairs that share genes. The numbers on edges (1 or 2) specify the count of shared genes. FDR values for each pathway are shown in blue under the pathway label. This network summarizes how ECM remodeling, immune/inflammatory signaling, epigenetic/HOTAIR regulation, and transport/metabolic programs are interrelated within the MMP12 signaling landscape.

The network shown in Figure 3 illustrates pathway interconnectivity and organizes the enriched results into four functional modules: (i) ECM remodeling, (ii) immune/inflammatory signaling (IL-1, TCR, osteoclast), (iii) HOTAIR/epigenetic regulation with post-translational links, and (iv) transport/metabolic processes (IGF and molecular transport). Shared-gene connections identify CD44, NFKBIA, PSME3, and MMP12 as multifunctional bridges linking these modules. Collectively, this network positions MMP12 at the center of an integrated program coordinating matrix turnover, inflammatory signaling, and growth-factor regulation, consistent with the experimentally observed anti-metastatic and immunomodulatory effects.

### 2.2. Experimental Biological Validation

To translate network predictions into functional evidence, we implemented a multilayer validation cascade spanning enzyme, cellular, and molecular readouts. Although our previous work demonstrated that indole-3-acetic acid derivatives exhibited the strongest enzymatic inhibition of MMP-12 among the tested scaffolds, the lead compounds identified in the present study (**C1**, **C7**, **C9**, **C10**, and **C15**) originate from different chemical families. Specifically, **C7** is a cinnamic acid derivative; **C1** is a para-chloro-cinnamic acid analogue; **C9** and **C10** are ferulic acid derivatives; and **C15** is derived from a 2-chloro-benzoyl chloride scaffold. First, we quantified the MMP12 enzymatic inhibition of the newly synthesized inhibitors (**C1**–**C6**), whereas the inhibition data for **C7**–**C27** and **H1**–**H4** had been previously reported [40]. Details of the chemical synthesis and structure elucidation for compounds (**C1**–**C6**) are presented in the Supplementary Material, along with the corresponding figures (Figures S1–S6, Schemes S1 and S2, and Table S3). We then profiled antiproliferative activity across NSCLC lines (A549, H1299, H661) over 48–96 h (Table S4), followed by phenotypic assays of migration (wound healing at IC_50_, ½ IC_50_, ¼ IC_50_) (Figures S7–S11 and Table S5) and anchorage-independent growth (soft agar) (Figures S12–S16 and Table S6). Mechanistically, we assessed target engagement by qPCR (MMP12 and prioritized downstream genes at 0.1× IC_50_) (Figures S17 and S18) and Western blot (MMP12 protein at 0.1, ¼, ½ IC_50_) (Tables S7–S9) and characterized cell cycle and apoptosis responses by flow cytometry (Table S10). All assays were performed in replicate with appropriate controls; normal dermal fibroblasts were included for preliminary tolerability assessment.

#### 2.2.1. Effect of MMP12 Inhibitors on Enzyme Activity

The enzyme inhibition potential of synthesized *p*-chlorocinnamic acid derivatives against MMP12 was assessed using the Abcam colorimetric assay [40]. At 50 µM, inhibition ranged from 11.5% to 35.0%, with **C2** as the most active (35.0%) and **C5** the least (11.5%). Other derivatives showed low to moderate effects: **C1** at 32.9%, **C3** at 23.5%, **C4** at 23.3%, and **C6** at 21.3%, resulting in a mean inhibition of approximately 24.6%.

#### 2.2.2. Effect of MMP12 Inhibitors on the Viability of Lung Cancer Cells

To evaluate the impact of MMP12 inhibitors on lung cancer cell lines, an MTT assay was performed on A549, H1299, and H661 cells exposed to varying concentrations of inhibitors over 48, 72, and 96 h. Significant antiproliferative effects were noted, with decreased viability in treated cells compared to controls (Table S4). Compound **C1** showed strong activity against all cell lines at 72 h, with IC_50_ values of 118 µM (A549), 91 µM (H1299), and 34 µM (H661). Compound **C7** also affected H1299 and H661, yielding IC_50_ values of 48 µM and 52 µM, respectively. Compound **C9** showed values of 99.7 µM (A549), 43.4 µM (H1299), and 51.3 µM (H661), while **C10** had IC_50_ values of 101.2 µM, 65.5 µM, and 55.8 µM for the three cell lines. Compound **C15** yielded IC_50_ values of 145 µM and 130 µM, respectively. The IC_50_ values of compound **C1**, **C7**, **C9**, **C10**, and **C15** against the H1299 cell line are listed in Table 4. Evaluation in normal dermal fibroblasts indicated reasonable tolerance and cellular safety at the tested concentrations.

#### 2.2.3. Effect of MMP12 Inhibitors on H1299 Lung Cancer Cells Migration

**Figure 4:**
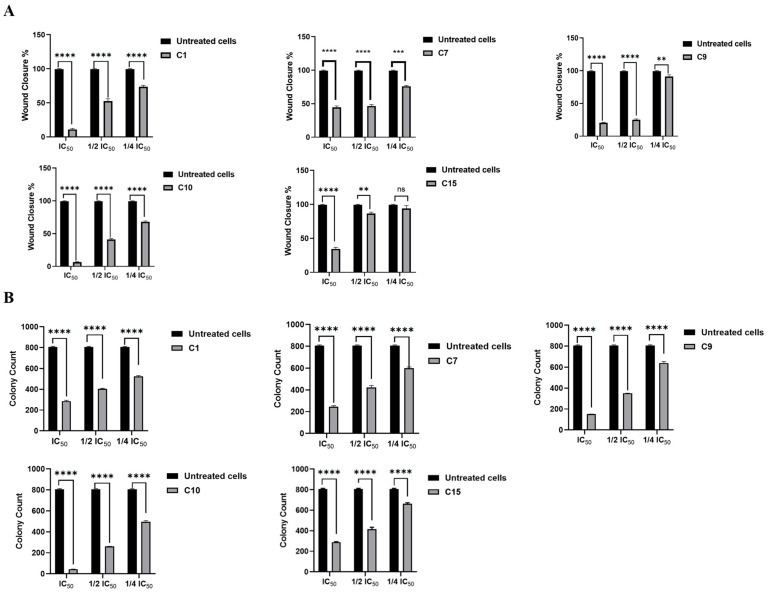
MMP12 inhibitors suppress migration and clonogenicity in H1299 cells: (**A**) Wound-healing assay showing percent closure after 48 h treatment with compounds **C1**, **C7**, **C9**, **C10**, or **C15** at indicated concentrations relative to IC_50_. (**B**) Soft agar colony formation assay following 72 h pretreatment. Data represent mean ± SD from n independent experiments normalized to untreated controls. Statistical significance was determined using GraphPad’s Prism 9 (** *p* ≤ 0.01, *** *p* ≤ 0.001, **** *p* ≤ 0.0001). ns: not significant.

To assess the impact of MMP12 inhibitors on the migration of H1299 lung cancer cells, a wound healing assay was conducted using IC_50_, ½ IC_50_, and ¼ IC_50_ concentrations of compounds **C1**, **C7**, **C9**, **C10**, and **C15**. After 48 h, untreated cells showed nearly complete wound closure. In contrast, MMP12 inhibitors significantly reduced cell migration in a concentration-dependent manner (Table S5). Specifically, treatment with **C1** (91.7, 45.9, and 23 µM), **C7** (48.1, 24.1, and 12.1 µM), **C9** (43.2, 21.7, and 10.9 µM), **C10** (65.5, 32.7, and 16.4 µM), and **C15** (145.2, 72.6, and 36.3 µM) resulted in approximately 90%, 50%, and 28%; 54%, 52%, and 23%; 80%, 76%, and 11%; 94%, 60%, and 33%; and 67%, 15%, and 9% inhibition of wound closure, respectively. The effects are illustrated in Figure 4A, with additional wound images in Figures S7–S11 in the Supplementary Materials.

#### 2.2.4. Effect of MMP12 Inhibitors on Anchorage-Independent Growth of H1299

To investigate the impact of MMP12 inhibitors on the colony formation capabilities of H1299 cancer cells, the cells were treated with either IC_50_, ½ IC_50_, or ¼ IC_50_ concentrations of **C1**, **C7**, **C9**, **C10**, and **C15** for 72 h. When compared to untreated control cells, treatment with MMP12 inhibitors (**C1**, **C7**, **C9**, **C10**, and **C15**) inhibited the formation of cell colonies by reducing both the number and size of colonies. The effect of MMP12 inhibitors on the colony numbers and size histogram views is shown in Table S6, Figure 4B, with statistical significance. Figures S12–S16 show a selection of images captured on day 15 at different magnifications: 4× and 20×.

#### 2.2.5. Effect of MMP12 Inhibitors on Gene Expression of H1299 Lung Cancer Cells

Quantitative real-time PCR (qPCR) was used to assess the impact of MMP12 inhibition in H1299 lung cancer cells. Cells were treated with synthesized inhibitors (**C1**, **C7**, **C9**, **C10**, and **C15**) at a concentration of 0.1× IC_50_. An initial baseline assessment of *MMP12* expression across three untreated lung cancer cell lines (H1299, A549, and H661) using the 2^−ΔCt^ method identified H661 as the highest expressor, followed by H1299, and A549 as the lowest (Figure S17). All five compounds significantly downregulated *MMP12* mRNA in H1299 cells, confirming successful target engagement (Table 5). Analysis of downstream targets revealed a complex transcriptional response: while *NFKBIA* and *ADMA9* were upregulated, *CD44*, *CCL15*, *PSME3*, and *SPARCL1* were consistently downregulated (Figure S18). *APOA1* exhibited variable, compound-specific regulation. These findings demonstrated that pharmacological inhibition of MMP12 induces significant and multifaceted shifts in the expression of genes central to cancer-associated pathways.

#### 2.2.6. Effect of MMP12 Inhibitors on Protein Expression in H1299 Lung

**Figure 5:**
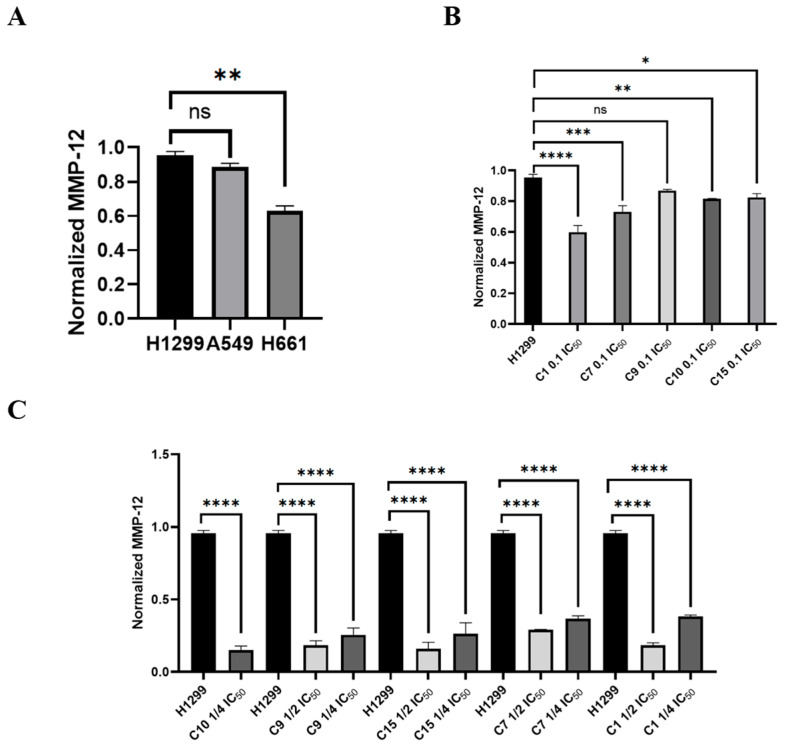
Effects of MMP12 inhibition on MMP12 protein expression in lung cancer cells: (**A**) Normalized basal MMP12 protein expression in untreated H1299, A549, and H661 lung cancer cell lines. (**B**) Effect of MMP12 inhibitors at 0.1 IC_50_ concentration on MMP12 and GAPDH protein levels in H1299 cells relative to untreated control. (**C**) Effect of MMP12 inhibitors at ¼ IC_50_ and ½ IC_50_ concentrations on MMP12 and GAPDH protein levels in H1299 cells relative to untreated control. Protein expression levels were evaluated based on band size and densitometric intensity. MMP12: matrix metalloproteinase-12; GAPDH: glyceraldehyde 3-phosphate dehydrogenase. (* *p* ≤ 0.05, ** *p* ≤ 0.01, *** *p* ≤ 0.001, **** *p* ≤ 0.0001). ns: not significant.

Western blot analysis was performed to evaluate MMP12 expression in H1299 cells treated with MMP12 inhibitors **C1**, **C7**, **C9**, **C10**, and **C15** at 0.1, ¼, and ½ IC_50_ concentrations, using GAPDH as a loading control (Figure 5B,C). Prior to treatment, baseline MMP12 levels were assessed in untreated lung cancer cell lines H1299, A549, and H661 (Figure 5A), with H1299 showing the highest expression. Results showed a significant reduction in MMP12 expression in inhibitor-treated cells compared to controls (Tables S6–S8). **C10** was the most potent, completely eliminating detectable protein at ¼ IC_50_. **C15** and **C9** also effectively suppressed MMP12, while **C1** and **C7** were less effective. At 0.1 IC_50_, complete inhibition was not achieved.

#### 2.2.7. Effect of MMP12 Inhibitors on Cell Cycle of H1299 Lung Cancer Cell Line

**Figure 6:**
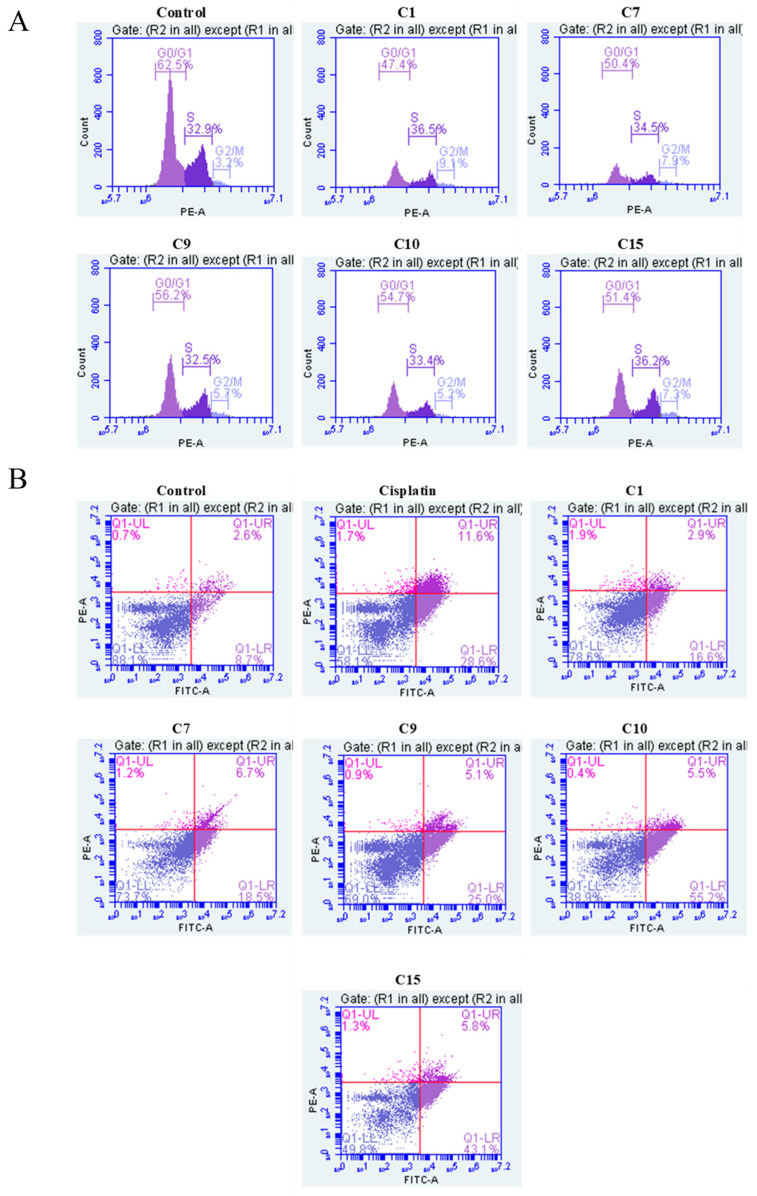
Effect of MMP12 inhibitors on cell cycle and apoptosis in H1299 cells: (**A**) Cell cycle distribution in treated and control samples of H1299 cells. H1299 cells were treated with each compound at their 1/2 IC_50_ concentrations for 48 h, followed by PI staining and flow cytometric analysis. (**B**) Apoptosis analysis by Annexin V-FITC/PI dual staining in H1299 cells. Representative dot plots show the distribution of cells across four populations: viable (Annexin^−^/PI^−^, lower left), early apoptotic (Annexin^+^/PI^−^, lower right), late apoptotic (Annexin^+^/PI^+^, upper right), and necrotic (Annexin^−^/PI^+^, upper left).

Flow cytometry was used to evaluate the effects of compounds (**C1**, **C7**, **C9**, **C10**, and **C15**) at their ½ IC_50_ concentrations on the cell cycle progression of H1299 cells. The untreated control group showed a typical proliferative profile, with 62.5% of cells in the G0/G1 phase (Figure 6A). Treatment with these compounds significantly altered the cell cycle distribution (Figure S19). Compound **C1** had the most pronounced effect, reducing the G0/G1 population to 47.4% while increasing the S phase to 36.5% and G2/M phase to 9.1%. Compounds **C7** and **C15** caused moderate reductions in G0/G1 of 50.4% and 51.4%, respectively. In contrast, **C9** and **C10** retained more cells in G0/G1, at 56.2% and 54.7%, respectively.

Overall, **C1** demonstrated the most disruptive effect on cell cycle regulation in H1299 cells, while **C9** and **C10** showed milder cytostatic actions, and **C7** and **C15** exhibited intermediate effects. These findings suggest that the mechanisms involved are p53-independent, especially relevant in the context of p53-deficient H1299 cells.

#### 2.2.8. Effect of MMP12 Inhibitors on Apoptosis of H1299 Lung Cancer Cell Line

Flow cytometric analysis using Annexin V-FITC/PI dual staining assessed apoptosis induction in H1299 cells after 72 h treatment with compounds at double IC_50_ concentrations. Untreated controls had high viability (88.1%) and low apoptosis (11.3%). Cisplatin reduced viability to 58.1% and increased total apoptosis to 40.2%. Among the tested compounds, **C10** had the strongest pro-apoptotic effect, decreasing viability to 38.9% and inducing 60.7% total apoptosis, mainly through early apoptosis (55.2%). **C15** showed similar effects with 49.8% viability and 48.9% total apoptosis. Moderate activity was noted with **C9** (31.1% total apoptosis) and **C7** (25.2%). **C1** had the mildest impact with 78.6% viability and 19.5% total apoptosis. All compounds favoured early apoptosis, indicating rapid apoptotic signaling without significant secondary necrosis (Figure 6B and Figure S20).

### 2.3. Mechanism Elucidation Using Bioinformatics Methods

To systematically decipher the molecular mechanisms underlying the transcriptional changes observed following MMP12 inhibition, we employed a multifaceted bioinformatics approach. This involved identifying the upstream regulators responsible for the gene expression shifts, reconstructing the integrated signaling network downstream of MMP12, and evaluating the clinical relevance of the key genes as biomarkers backed by sound computational evidence.

#### 2.3.1. Identifying Upstream Regulators Based on Experimental Expression Data

To identify the upstream regulators responsible for the observed transcriptional changes following MMP12 inhibition, we conducted an upstream regulator analysis in IPA using the fold-change values from the five compound treatments. The analysis was filtered to focus on statistically significant (*p*-value < 0.05), protein-coding regulators (e.g., enzymes, kinases, transcriptional regulators), excluding non-biological entities. The identified top 10 regulators for each compound are summarized in Table 6.

This analysis revealed a core set of conserved regulators across four compounds (**C1**, **C7**, **C10**, and **C15**). This core network featured consistently inhibited nodes, such as the AKT family and kinases MAP3K11/MAP2K7, alongside activated regulators like thioredoxin (TXN). Notably, MMP12 itself was also predicted to be an activated upstream regulator (z-score = +1.73), despite its mRNA and protein levels being downregulated by our inhibitors.

This pattern suggests a common mechanism suppressing survival and stress-response pathways. In contrast, Compound **C9** engaged a unique regulatory network involving *CRK/CRKL*, SMAD2/3/4, and ERBB family, indicating a distinct mechanism potentially operating through TGF-β and growth-factor signaling.

#### 2.3.2. Reconstruction of an Integrated Signaling Mechanism

**Figure 7:**
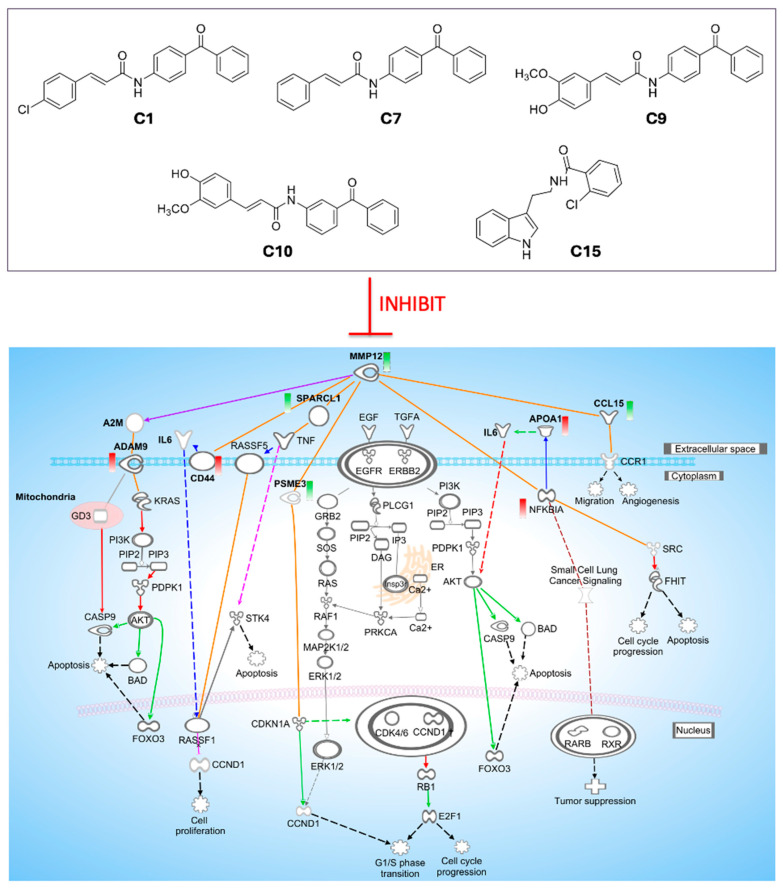
Lead MMP12 inhibitors and their reconstructed signaling network in NSCLC. The top panel shows the chemical structures of the five validated inhibitors (**C1**, **C7**, **C9**, **C10**, and **C15**). The bottom panel shows the reconstructed pathway map of MMP12 and its downstream effectors uncovered from experimental results following inhibitor treatment. Node color on the map indicates expression change (red: upregulated; green: downregulated). Edges show interaction types (solid: direct; dashed: indirect; red: activation; green: inhibition). Cellular compartments provide spatial context.

We synthesized the study findings into a coherent mechanistic model centered on the seven validated downstream genes, using curated interactions from IPA and published literature (Figure 7). The generated network revealed that MMP12 inhibition coordinately regulates apoptosis, proliferation, and metastatic programs, albeit with a critical compensatory response. The tumor-suppressive outcome is driven by combined molecular changes: downregulation of *SPARCL1* and *PSME3*, coupled with upregulation of the NF-κB inhibitor *NFKBIA* and downregulation of *CD44* and *CCL15*, collectively promotes apoptosis while suppressing proliferation, migration, and angiogenesis.

However, this therapeutic effect may be countered by a compensatory axis, evidenced by consistent *ADAM9* upregulation and its connection to pro-survival KRAS-PI3K-AKT signaling. The context-dependent regulation of *APOA1* further underscores the network’s complexity. This model establishes that the net anti-tumor activity results from this balance, while suggesting that co-targeting the ADAM9-driven axis could significantly enhance therapeutic efficacy.

#### 2.3.3. Biomarker Identification

Using IPA’s Biomarker Filter (Homo sapiens; cancer; lung cancer cell lines), three genes in our panel mapped to established biomarkers (not specifically for MMP12 inhibition): *ADAM9* (efficacy/prognosis), *CD44* (diagnosis/progression/prognosis), and MMP12 (efficacy). In contrast, *NFKBIA*, *PSME3*, *SPARCL1*, *CCL15*, and *APOA1* did not return established entries under these filters and at the time of query, indicating that, within IPA, they are not currently categorized as clinical lung cancer biomarkers. This does not preclude literature support elsewhere; rather, it suggests these genes are less characterized in this context and may represent novel pharmacodynamic (PD) readouts of MMP12 pathway inhibition.

Given their robust, directionally consistent modulation upon MMP12 inhibition in our experiments, we nominate a composite eight-gene PD panel for MMP12-targeted therapy in NSCLC: *NFKBIA*, *PSME3*, *SPARCL1*, *CCL15*, *APOA1*, *ADAM9*, and *CD44*, with *MMP12* serving as the direct target anchor. These markers warrant prioritization for clinical testing (multiplex qPCR/protein assays, ROC analyses in patient cohorts, correlation with treatment response, longitudinal sampling) to refine a clinically deployable PD signature.

## 3. Discussion

This study established an integrative, informatics- and network-guided framework to evaluate MMP12 as a therapeutic target in lung cancer. As outlined in Figure 1, the study proceeded through hypothesis generation (bioinformatics), multilayer experimental validation (gene, protein, phenotype), and mechanism reconstruction, integrating both datasets [41,42,43]. Using this three-stage design, we demonstrated that pharmacological inhibition of MMP12 produces coordinated antimigratory, anti-clonogenic, and pro-apoptotic effects in NSCLC cells. Critically, this approach also revealed a robust compensatory axis centered on *ADAM9*, which clarifies the limitations of single-agent therapy and points to rational combination strategies. A key feature of this study’s design is that bioinformatics analyses prospectively defined the experimental endpoints. The phenotypic assays, including proliferation, migration, and clonogenicity, in addition to the seven-gene pharmacodynamic signature, were not selected retrospectively but were pre-specified from network analysis and enriched pathways, including ECM remodeling, immune signaling, PI3K–AKT, and HOTAIR pathways. This approach created a closed loop from computational prediction to experimental measurement and mechanistic validation.

Our initial bioinformatic analysis mined MMP12 interactions to identify 113 downstream targets enriched in ECM remodeling, PI3K-AKT signaling, and immune pathways (Figure 2A). Noting that most downstream effects were proteolytic, while three nodes (*NFKBIA*, *PSME3*, *SPARCL1*) were uniquely annotated as transcriptionally regulated, we developed a transparent 0–5 scoring rubric. This system integrated evidence of direct or transcriptional regulation with overlap from lung cancer-relevant gene sets contributing to pathogenesis (apoptosis, migration, and expressed in A549) to prioritize candidates. This triage nominated seven genes (*CD44*, *NFKBIA*, *ADAM9*, *PSME3*, *SPARCL1*, *CCL15*, and *APOA1*) for experimental validation (Figure 2B; Table 2). Pathway enrichment on this nominated gene panel highlighted HOTAIR, ECM degradation, and IL-1/TCR signaling, placing MMP12 at the nexus of matrix dynamics, inflammatory reprogramming, and growth-factor control (Figure 3; Table 3). Collectively, these regulatory circuits converge on core oncogenic hallmarks, including sustained proliferation, immune-driven inflammation, and ECM remodeling, all of which are tightly linked to aggressive lung cancer progression [44,45,46,47,48].

Phenotypic screening of a 31-compound library of designed MMP12 inhibitors [40] yielded five leads (**C1**, **C7**, **C9**, **C10**, and **C15**) with time-dependent anti-proliferative activity in H1299 cells (Table 4). At sub-cytotoxic doses, all five compounds robustly affected hallmarks of aggressiveness: each significantly curtailed wound closure in scratch assays, indicating impaired motility (Figure 4A; Table S5), and each reduced anchorage-independent growth in soft agar, reflecting diminished clonogenic potential under non-adherent conditions (Figure 4B; Table S6). This anti-metastatic profile is concordant with the expected consequences of disrupting MMP12-driven ECM turnover and survival signaling and with prior links between MMP12, EMT, and the uPA/uPAR/TGF-β/AKT axis in NSCLC [20,25,49]. Notably, the enriched pathways from our informatics stage anticipated these readouts: ECM–receptor interaction and focal adhesion aligned with suppressed migration and clonogenicity; PI3K–AKT and TNF signaling with reduced proliferation and increased apoptosis; and cytokine/cell cycle networks with the observed shifts in cell cycle distribution.

Gene expression analysis confirmed successful target engagement, showing universal MMP12 downregulation and coherent modulation of its predicted downstream signaling network in treated lung cancer cells (Table 5; Figure S18). The validated expression signature was distinctive: *NFKBIA* increased ~18–29-fold, consistent with enhanced IκBα-mediated restraint of NF-κB; *CCL15* decreased by ~95–97%, indicating diminished chemotactic drive; and *ADAM9* rose up to ~267-fold, suggesting an adaptive response. At the protein level, Western blot analysis corroborated the transcriptomic findings, showing a clear, dose-dependent reduction in MMP12 abundance (Figure 5; Tables S6–S8). The concordance between mRNA and protein data provides strong validation of target suppression at multiple biological layers. Collectively, these results confirm the efficacy of the intervention in engaging MMP12, modulating downstream effectors, and reshaping the molecular landscape of lung cancer cells toward a less invasive and less inflammatory state.

Upstream regulator analysis provided key insights that informed our mechanistic model. First, it paradoxically predicted MMP12 activation (positive z-score) despite its pharmacological inhibition. This is a recognized phenomenon where the score reflects downstream network activity rather than the expression level of the upstream regulator itself [50]. The signature of its downstream targets suggests that compound inhibition may trigger a feedback response that over-activates MMP12-regulated pathways. Second, the analysis revealed a conserved inhibition of survival kinases (AKT, MAP3K11, MAP2K7) alongside the pronounced upregulation of ADAM9, which was predicted to interface with KRAS-PI3K-AKT and ERBB networks. This positions ADAM9 as a central feature of a compensatory resistance axis, supported by evidence that it activates PI3K-AKT signaling, reactivates ERBB pathways, and promotes survival through ligand shedding. Mechanistically, MMP12 inhibition may alter α2-macroglobulin processing (a known MMP12 substrate that regulates ADAM9), thereby inducing ADAM9 expression and its pro-survival signaling through AKT-mediated suppression of BAD [51,52,53,54,55,56,57]. Although ADAM9 can contextually promote GD3-caspase-9-mediated apoptosis [58,59,60,61], its consistent and strong upregulation alongside key survival networks may suggest that its oncogenic role predominates in this setting. Thus, ADAM9 induction represents a key adaptive resistance mechanism, revealing both a limitation of single-agent MMP12 inhibition and a rationale for combination therapy targeting this escape pathway.

Synthesizing these results, we propose a compact mechanistic model (Figure 7). In this model, the downregulation of *SPARCL1* and *PSME3* promotes apoptosis and cell cycle arrest, while the upregulation of *NFKBIA* and the downregulation of *CD44* and *CCL15* suppress survival signaling and metastatic capacity. This framework is supported by prior studies showing that *SPARCL1* loss enhances *TNF*-mediated apoptosis and weakens ECM-derived survival cues [62,63,64], while reduced *PSME3* strengthens p21-RB checkpoint control and promotes G0/G1 arrest [65,66,67]. Furthermore, *NFKBIA* upregulation suppresses NF-κB survival signaling and activates *SRC-FHIT* tumor-suppressive pathways [68,69,70], and *CCL15* downregulation diminishes chemotactic and angiogenic activity [71]. The role of *ADAM9* as a compensatory node is reinforced by its known ability to engage *KRAS-PI3K-AKT* and *ERBB* networks, where it can paradoxically promote survival or trigger mitochondrial apoptosis depending on context [51,52,53,54,55,56,57].

*Translational biomarker implications*

Our study revealed that the experimentally validated eight-gene panel (*MMP12*, *CD44*, *ADAM9*, *NFKBIA*, *PSME3*, *SPARCL1*, *CCL15*, and *APOA1*) captures both on-target engagement (MMP12 downregulation) and a predictable adaptive response (*ADAM9* upregulation). Conceptually, the panel is positioned as a PD signature (not a baseline prognostic set): it should change directionally with MMP12 blockade and could (i) verify target engagement in early phase trials, (ii) quantify pathway suppression vs. adaptation, and (iii) guide combination strategies (e.g., multi-targeting ADAM9/ERBB/AKT in patients showing *ADAM9* overexpression). Practical deployment can leverage multiplex qPCR or targeted proteomics from minimally invasive specimens (e.g., liquid biopsy-derived RNA).

In conclusion, by employing a network-guided framework from discovery to experimental validation, this work not only delivers a validated pharmacodynamic signature for MMP12 inhibition and confirms its potent anti-tumor phenotypes but also identifies an *ADAM9*-driven adaptive response as a putative central determinant of therapeutic durability that awaits further testing. The discovery of the *ADAM9*-driven adaptive response not only identifies a likely limitation of single-agent therapy but also provides a clear rationale for future combination strategies targeting ERBB or AKT pathways. To translate these findings, future work must validate this signature across diverse NSCLC models in vivo and evaluate rational drug combinations to overcome resistance and achieve durable therapeutic outcomes.

## 4. Materials and Methods

### 4.1. Databases and Software

#### 4.1.1. Cortellis Drug Discovery Intelligence (CDDI) Database

The Cortellis Drug Discovery Intelligence (CDDI) database, provided by Clarivate Analytics, served as the initial source of information for MMP12 inhibitors [72]. CDDI integrates a wide array of data, including peer-reviewed literature, experimental pharmacology, clinical trial records, and patent filings, making it an invaluable resource for early-stage drug discovery. The database enables systematic exploration of targets, associated biomarkers, and therapeutic indications. In this study, CDDI was searched using the term “MMP12” under the biomarker types of “gene” and “protein,” specifically in the context of “cancer.” The retrieved information included reported inhibitors along with their corresponding pharmacological data, providing essential background for the subsequent bioinformatics analyses.

#### 4.1.2. MetaCore^TM^

MetaCore^TM^ version 21.4 (build 70,700) [73] was used to explore the molecular landscape of MMP12 molecular interactions to gain a better understanding of its functional networks. This platform offers manually curated ontologies, canonical pathways, and process networks that are supported by experimental evidence. It also enables the visualization of molecular interactions, highlighting mechanisms, directionality, and biological effects in a clear manner. In this study, we specifically queried MetaCore^TM^ for the “outgoing” signaling interactions of MMP12 to identify downstream genes/proteins and their functional associations with cancer. This approach helped prioritize candidate genes that may be transcriptionally or functionally regulated by MMP12 in the context of lung cancer progression. All retrieved interacting genes/proteins were represented in terms of these gene symbols per the HUGO Gene Nomenclature Committee (HGNC) [74].

#### 4.1.3. Cytoscape

Cytoscape (version 3.10.3) is an open source platform designed for the visualization and analysis of complex biological networks [75]. Cytoscape is widely utilized for exploring molecular interaction data because it consolidates extensive collections of both experimentally validated and computationally predicted interactions from public databases. In this study, Cytoscape was utilized to visualize the interaction landscape of MMP12 and its associated proteins. This approach allowed for a clear interpretation of connectivity patterns, relationships between nodes, and interactions at the pathway level. The STRING app within Cytoscape was employed to import high-confidence protein–protein interaction data.

#### 4.1.4. Ingenuity Pathway Analysis (IPA)

Ingenuity Pathway Analysis (IPA, QIAGEN Redwood City, CA, USA) was used to explore the biological role of MMP12 [76]. This involved performing enrichment analysis to identify the most significantly enriched canonical pathways, disease associations, and cellular functions related to MMP12. Additionally, causal network analysis was conducted to predict potential upstream regulators and downstream molecular interactions. IPA is specifically designed to aid in interpreting complex biological datasets by mapping genes and proteins onto well-curated pathways, molecular functions, and disease mechanisms. This approach allowed for the construction of a mechanistic framework for MMP12 in lung cancer, providing insights into its role in cancer-related signaling and progression.

### 4.2. Systems Biology 

#### 4.2.1. Systems Biology Informatics Workflow

A bioinformatics framework developed by Hajjo et al. [41,42,77,78] was used in this study. This computational pipeline combines curated database mining, network-based prioritization of targets and genes, pathway enrichment analysis, and the inference of causal regulators. Candidate genes were identified from high-confidence biological databases and prioritized based on their relevance to the disease and predicted regulatory associations. The prioritized biomarkers were then experimentally validated in target-inhibited cells, followed by an analysis of pathways and upstream regulators related to the confirmed gene signature. Network visualization and mechanistic mapping were employed to contextualize molecular interactions and emphasize biologically relevant signaling programs. This adaptable framework has been successfully applied in various biological contexts, and in this study, it was tailored to explore the regulatory landscape of MMP12 in NSCLC.

#### 4.2.2. Mining Interactions

*Identification of downstream targets of MMP12*

MetaCore^TM^ was utilized to systematically identify the genes regulated by MMP12. Understanding these downstream targets is crucial because they represent the functional consequences of MMP12 activity and inhibition, which helps clarify the role of this enzyme in lung cancer. The database was searched using the keyword “MMP12,” with the species filter set to *Homo sapiens*. From the search results, the “Interactions” option was selected, and the interaction type was filtered to “Outgoing” to obtain a comprehensive list of genes that are functionally regulated by MMP12. The resulting gene list was exported for further processing, including conversion to official HUGO Gene Nomenclature Committee (HGNC) gene symbols to standardize identifiers, and de-duplication to ensure a non-redundant dataset. This collection of downstream genes served as the foundation for subsequent focused analyses.

*Identification of Genes Annotated with “Influence on Expression” from MMP12*

A separate analysis was conducted using MetaCore^TM^ to identify genes that are transcriptionally regulated by MMP12. From the outgoing interaction list, the “Mechanism” filter was applied and set to “Transcription Regulation” to identify genes whose expression is controlled at the transcriptional level by MMP12. The filtered gene set was identified as a panel of targets that are regulated at the transcriptional level.

*Identification of Direct Targets of MMP12*

To identify genes directly regulated by MMP12, the previously generated outgoing gene list was uploaded into MetaCore’s network building tool. MMP12 was allocated as the central node, and the “Shortest Path” algorithm was applied using the one-step setting. This approach allowed us to focus exclusively on direct, first-degree curated interactions. This approach ensured that only direct molecular connections between MMP12 and its target genes were captured.

#### 4.2.3. Identification of Cancer-Associated Genes

To identify genes associated with MMP12 and their role in lung cancer, the advanced search function in MetaCore^TM^ was utilized. The database was queried using keywords related to the disease and relevant processes, including “apoptosis”, “migration”, “gene expressed in A549”, and “genes expressed in lung cancer”. The resulting sets of genes were compiled and used in computational analyses to assess their potential contribution to the progression of lung cancer.

*Identification of Overlapping Functional Sets*

The intersections among the retrieved gene sets from MetaCore^TM^ were analyzed to identify candidates that appeared in multiple cancer-related categories in addition to MMP12. These overlapping genes are considered particularly important, as their recurrence suggests a stronger and more central role in linking MMP12 to critical pathways in lung cancer. To illustrate this overlap, a Venn diagram was created, visually representing the convergence of the gene sets and highlighting the significance of genes that are present in multiple categories [79].

#### 4.2.4. Prioritize MMP12-Responsive Genes for Expression Testing

To refine the list of genes regulated by MMP12 for experimental validation, we used a predictive strategy that combined interconnectivity mapping with mechanistic scoring. This approach aimed to prioritize genes that not only showed a bioinformatic association with MMP12 but also demonstrated convergence across functional categories related to cancer. The process involved using a structured scoring system that ranked the genes based on their likelihood of being modulated downstream by MMP12 and evaluated their overlap with gene sets relevant to cancer.

A scoring system was designed around three mechanistic criteria. First, genes identified as direct targets of MMP12 through shortest-path network analysis in MetaCore^TM^ were assigned 1 point; genes without direct interaction received 0 points. Second, genes annotated in MetaCore^TM^ with an “influence on expression” relationship from MMP12 to the gene were also assigned 1 point. Third, genes were scored based on the number of functional gene lists in which they overlapped with the MMP12 outgoing gene list: 1 point for overlapping with one other list, 2 points for two lists, and 3 points for overlapping with three additional gene sets. The total cumulative score (ranging from 0 to 5) was used to rank gene relevance for downstream validation.

#### 4.2.5. Canonical Pathway Enrichment of the Prioritized Genes

To investigate the biological significance and pathway associations of the selected genes, Ingenuity Pathway Analysis (IPA, QIAGEN Inc., Hilden, Germany) was employed using a gene list-based approach [76]. The list of prioritized genes was uploaded to IPA, and the gene symbol column was manually designated as the molecular identifier (ID).

The analysis was conducted using default parameters. IPA automatically mapped the genes to its curated knowledge base and performed enrichment analysis to identify associated canonical pathways.

Statistical significance was determined using IPA’s right-tailed Fisher’s exact test, which evaluates whether the observed overlap between the input genes and each pathway exceeds what would be expected by chance. The significance of the enrichment was assessed using their hypergeometric *p*-values, with *p*-values less than 0.05 considered statistically significant.

#### 4.2.6. Upstream Regulator Inference from Expression Datasets

To identify potential upstream regulators responsible for the observed gene expression changes following MMP12 inhibitor treatment, expression-based analysis was performed using IPA [76].

In this phase of the analysis, we examined causal regulatory networks influenced by compound treatment using five expression datasets corresponding to treatments with **C1**, **C7**, **C9**, **C10**, and **C15**. Each dataset included gene symbols, log_2_ (fold change) values representing expression changes after treatment, and *p*-values comparing treated cells to the untreated control group. Upon uploading each dataset to IPA, the gene symbol column was manually assigned as the molecular identifier (ID). In contrast, the log_2_ (fold change) and *p*-value columns were designated as observation values. Before running the Core Analysis, we retained all default settings except for the species filter, which was set to *Homo sapiens*. The platform then recalculated mapping to ensure that all uploaded genes were recognized and correctly matched. Core Analysis was conducted separately for each compound using log_2_ (fold change) as the expression measurement. No additional cutoffs for fold change or *p*-value were applied during the analysis, allowing all experimentally modulated genes to be included for downstream interpretation, including the generation of causal networks.

This method predicts the activation or inhibition state of upstream regulators by analysing observed gene expression patterns. The confidence, strength, and direction of these predictions are quantified using the activation z-score, where values greater than 2 indicate activation and values less than −2 indicate inhibition. Statistical significance is determined through Fisher’s exact test, based on the hypergeometric distribution, which calculates the probability that the overlap between differentially expressed genes and known regulator targets occurs by chance. Regulators with *p*-values below 0.05 are considered statistically significant, and all identified regulators are ranked according to their *p*-values.

#### 4.2.7. Biomarker Identifications

The Biomarker Filter tool in IPA was applied to the predefined gene list to identify potential biomarkers. The analysis was restricted to *Homo sapiens*, limited to the disease category ‘cancer,’ further refined to lung cancer cell lines, and all biomarker types were included for detection.

#### 4.2.8. Mechanism Reconstruction

An integrated mechanistic network was developed to investigate and model the role of MMP12 and its inhibition in lung cancer, utilizing the IPA platform. A disease-focused map for non-small cell lung cancer was created by searching the IPA database with the term “MMP12 in lung cancer.” Using the Path Designer tool, the map was expanded into a mechanistic network that illustrates the functional role of MMP12 and the downstream effects of the differentially expressed genes that have been experimentally validated and are associated with lung cancer progression.

### 4.3. Synthesis of p-Chlorocinnamic Acid Derivatives (***C1**–**C6***)

The cinnamic acid derivatives (**C1**–**C6**) were synthesized using the validated protocol outlined in our previous publication [40]. Complete experimental details can be found in the Supplementary file.

### 4.4. Experimental Validation

#### 4.4.1. Cell Lines and Cell Maintenance

The lung cancer cell lines referenced as A549, H1299, and H661 were acquired from the American Type Culture Collection (ATCC, Manassas, VA, USA). Fibroblast cells of dermal origin were used as a model for normal human tissue, which were also purchased from ATCC. A549, H1299, and H661 cells were maintained in the RPMI culture medium, while fibroblast cells were maintained in the DMEM high glucose culture medium. Both RPMI and DMEM culture media were supplemented with 100 U/mL penicillin, 0.1 mg/mL streptomycin to prevent bacterial growth, 10% FBS to promote cell growth, and 2 mM L-glutamine as an energy source. Cells were cultured in 75 cm^2^ or 25 cm^2^ flasks, depending on the cell count needed for the experiment, and incubated in an incubator at 37 °C with 95% humidity and 5% CO_2_.

#### 4.4.2. MMP12 Enzyme Inhibition Assay

The inhibitory activity of the newly synthesized compounds against MMP12 was assessed using a previously established enzymatic assay protocol, as described in our earlier study [40]. In brief, we conducted a colorimetric thiopeptide-based inhibition assay for MMP12 using recombinant human MMP12 and the reference inhibitor NNGH as a positive control. The IC_50_ values were calculated using GraphPad Prism version 9 (GraphPad Software, San Diego, CA, USA).

#### 4.4.3. Cell Viability Assay

The 3-(4,5-dimethylthiazol-2-yl)-2,5-diphenyltetrazolium bromide (MTT) assay was employed to evaluate cell proliferation as previously noted [80]. All experiments were performed in triplicate wells and were repeated at least twice independently. The percentage of the relative cell viability of treated cells versus the untreated cells (negative controls) was calculated using the following formula in Equation (1) (cell viability calculation):

(1) $$Cell\;viability\left(\%\right)=\frac{optical\;density\;of\;treated\;cells}{optical\;density\;of\;untreated\;cells}\times 100$$

Optical density data were retrieved using the MTT assay.

#### 4.4.4. Wound Healing Assay

The wound healing migration assay was conducted using H1299 cells, following an established protocol [81]. In brief, cells were seeded at a density of 35,000 cells per insert side and treated with compounds **C1**, **C7**, **C9**, **C10**, and **C15** at concentrations of IC_50_, ½ IC_50_, and ¼ IC_50_. Images were captured at 0 and 48 h, and wound closure was quantified using ImageJ. Digital images were analyzed for wound area using the ImageJ software version 1.53e [82].

#### 4.4.5. Soft Agar Colony Formation Assay

The anchorage-independent colony formation assay was conducted using a previously described method with minor modifications [83]. In summary, H1299 lung cancer cells (1 × 10^4^ cells per well) were embedded in soft agar and treated with compounds **C1**, **C7**, **C9**, **C10**, and **C15** at concentrations of IC_50_, ½ IC_50_, and ¼ IC_50_ in duplicates. After 14 days of incubation, the colonies were imaged using the EVOS XL Core microscope ()Thermo Fisher Scientific, Waltham, MA, USA. and quantified.

#### 4.4.6. Annexin V-FITC/Propidium Iodide Apoptosis Assay

Apoptosis was assessed using Annexin-V/PI staining, following the method described earlier with minor modifications [84]. H1299 cells (3 × 10^5^ cells per well) were treated for 72 h with double the IC_50_ concentrations of compounds **C1**, **C7**, **C9**, **C10**, and **C15**, with cisplatin serving as a positive control. After the treatment, the cells were collected and stained with Annexin-V-FITC and propidium iodide. The stained cells were then analyzed using a BD FACSCanto II flow cytometer (Becton, Dickinson and Company (BD), Franklin Lakes, NJ, USA) and FACSDiva software (Becton, Dickinson and Company (BD), Franklin Lakes, NJ, USA).

#### 4.4.7. Cell Cycle Analysis Assay

Cell cycle distribution was evaluated in H1299 cells using a modified version of the method described [84]. Specifically, approximately 8.5 × 10^5^ cells were cultured in each T-25 flask and treated for 48 h with half the IC_50_ concentrations of compounds **C1**, **C7**, **C9**, **C10**, and **C15**, along with untreated control cells. After the treatment period, the cells were fixed with ethanol, stained with a propidium iodide/RNase A solution, and analyzed using a BD FACSCanto II flow cytometer. The data were processed with BD FACSDiva software version 8 (BD Biosciences, San Jose, CA, USA).

#### 4.4.8. Real-Time Polymerase Chain Reaction

Quantitative real-time PCR (qPCR) was conducted as previously described [85]. Lung cancer cell lines (A549, H1299, H661) were seeded at approximately 4 × 10^5^ cells per T-25 flask and incubated for 24 h. The H1299 cells were treated for 72 h with 0.1× IC_50_ concentrations of compounds **C1**, **C7**, **C9**, **C10**, and **C15**, while A549, H661, and fibroblast cells served as untreated controls. Total RNA was extracted, reverse-transcribed into cDNA, and qPCR was performed using SYBR Green on an Applied Biosystems platform, Foster City, CA, USA. Gene expression was normalized to GAPDH, and the fold-change was calculated using the 2^−ΔΔCt^ method. The basal expression of MMP12 in untreated A549, H1299, and H661 cells was assessed using the 2^−ΔCt^ method. Primer sequences and annealing temperatures (T_a_) are listed in Table S2 (Supplementary Materials).

#### 4.4.9. Effect of MMP12 Inhibitors on MMP12 Protein Expression

Western blotting was conducted to assess the protein expression of MMP12 in lung cancer cell lines (A549, H1299, and H661), following the protocol established earlier [86]. In summary, cells were seeded and incubated for 24 h. After this, H1299 cells were treated for 72 h with 0.1×, 0.5×, and 0.25× IC_50_ concentrations of compounds **C1**, **C7**, **C9**, **C10**, and **C15**, while untreated cells served as controls. Total protein lysates were prepared, separated by SDS-PAGE, and transferred to nitrocellulose membranes. These membranes were then probed with primary antibodies against MMP12 and GAPDH. The protein bands were visualized using an HRP substrate and analyzed for relative expression.

#### 4.4.10. Statistical Analysis

Data analysis was performed using GraphPad Prism software (GraphPad Prism version 8.0.0 for Windows, GraphPad Software, San Diego, CA, USA). Two-way ANOVA, one-way ANOVA, and multiple *t*-tests determined the differences between treatment groups. Data is expressed as mean ± SD, and *p* < 0.05 was considered a statistically significant difference. IC_50_ values were calculated using a non-linear regression analysis. Wound area, colony size, colony numbers, and band quantification were measured using ImageJ software version 1.53e.

## 5. Conclusions

This study enhances our understanding of MMP12, an understudied therapeutic target in lung cancer, by combining computational biology with comprehensive cellular and molecular validation. The selected inhibitors (**C1**, **C7**, **C9**, **C10**, and **C15**) significantly reduced malignant behaviors, including cell viability, migration, and anchorage-independent growth, while also inducing G0/G1 cell cycle arrest and apoptosis. Following treatment, both the *MMP12* gene and protein expression levels were significantly decreased, confirming strong target engagement.

Through mechanistic reconstruction and analysis of upstream regulators, we found that inhibiting MMP12 disrupts the signaling pathways that regulate extracellular matrix remodeling, inflammation, cell cycle progression, and metastatic potential. These findings provide a solid mechanistic rationale for targeting MMP12 to reduce lung cancer aggressiveness, laying the groundwork for advancing these inhibitors toward in vivo assessments and future preclinical development.

## Figures and Tables

**Table 1:** Downstream targets of MMP12 identified via molecular interaction analysis.

Downstream Network Object	Gene Symbol *	Effect	Mechanism
C3b	*CR1*	Activation	Cleavage
CCL15	*CCL15*	Activation	Cleavage
CCL16	*CCL16*	Activation	Cleavage
CCL23	*CCL23*	Activation	Cleavage
MMP-2	*MMP2*	Activation	Cleavage
NFKBIA	*NFKBIA*	Activation	Transcription regulation
Osteopontin	*SPP1*	Activation	Cleavage
Stromelysin-1(MMP-3)	*MMP3*	Activation	Unspecified
TNF-alpha	*TNF*	Activation	Cleavage
TyrRS	*YARS1*	Activation	Cleavage
AEBP1	*AEBP1*	Inhibition	Cleavage
Alpha 1-antitrypsin	*SERPINA10*	Inhibition	Cleavage
APOA1	*APOA1*	Inhibition	Cleavage
APOL1	*APOL1*	Inhibition	Cleavage
Coagulation factor XII	*F12*	Inhibition	Cleavage
Collagen I	*COL1A1*	Inhibition	Cleavage
Collagen III	*COL3A1*	Inhibition	Cleavage
Collagen IV	*COL4A1*	Inhibition	Cleavage
CXCL13	*CXCL13*	Inhibition	Cleavage
EFEMP2	*EFEMP2*	Inhibition	Cleavage
Elastin	*ELN*	Inhibition	Cleavage
ENA-78	*CXCL5*	Inhibition	Cleavage
FasL(TNFSF6)	*FASLG*	Inhibition	Cleavage
Fibronectin	*FN1*	Inhibition	Cleavage
Fibulin-3	*EFEMP1*	Inhibition	Cleavage
Fibulin-5	*FBLN5*	Inhibition	Cleavage
GRO-1	*CXCL1*	Inhibition	Cleavage
IL-8	*CXCL8*	Inhibition	Cleavage
Laminin 1	*LAMA1*	Inhibition	Cleavage
Myelin basic protein	*MBP*	Inhibition	Cleavage
Nidogen	*NID*	Inhibition	Cleavage
OSF-2	*POSTN*	Inhibition	Cleavage
PLAUR	*PLAUR*	Inhibition	Cleavage
PSME3	*PSME3*	Inhibition	Transcription regulation
SERPINB1	*SERPINB1*	Inhibition	Cleavage
SPARCL1	*SPARCL1*	Inhibition	Transcription regulation
Tenascin-C	*TNC*	Inhibition	Cleavage
TFPI	*TFPI*	Inhibition	Cleavage
Thrombospondin 2	*THBS2*	Inhibition	Cleavage
Vitronectin	*VTN*	Inhibition	Cleavage

* Gene symbols per HUGO Gene Nomenclature Committee (HGNC). The full inventory of downstream genes and proteins, their functional annotations, and their regulatory links to MMP-12 (without specification of activation or inhibition) can be found in Supplementary Table S1.

**Table 2:** Prioritization of MMP12 downstream genes based on functional priority and pathological relevance.

Gene	Direct Effect	Transcriptional Effect	Overlapping Gene	Total Score
*CD44*	1	0	3	4
*NFKBIA*	1	1	2	4
*PSME3*	1	1	1	3
*SPARCL1*	1	1	1	3
*ADAM9*	0	0	3	3
*APOA1*	0	0	3	3
*CCL14*	0	0	3	3
*CCL15*	0	0	3	3
*ACTR3*	0	0	2	2
*ARPC1B*	0	0	2	2
*ATIC*	0	0	2	2
*CAPZA1*	0	0	2	2
*CAPZA2*	0	0	2	2
*CCL1*	0	0	2	2
*CCL16*	0	0	2	2
*CCL23*	0	0	2	2
*CORO1C*	0	0	2	2
*CXCL13*	0	0	2	2
*PPBP*	0	0	2	2
*MMP2*	1	0	0	1
*TNF*	1	0	0	1

Genes were ranked by a cumulative score (0–5) derived from three criteria: direct interaction (1 point for evidence of a direct effect), transcriptional regulation (1 point for annotation as a transcriptional target), and pathobiological overlap (1–3 points for co-occurrence in 1, 2, or 3 lung cancer-related gene sets, respectively). The resulting total score reflects the strength of evidence for each gene’s functional and pathological relevance to MMP12 signaling in lung cancer. Genes that received a total score of 0 are not listed. Color coding represents scoring tiers: dark orange = highest priority (total score 4); medium orange = transcriptionally regulated high-priority genes (score 3); light orange = non-transcriptional high-priority genes (score 3); grey/white medium priority (score 2).

**Table 3:** Top 10 enriched canonical pathways.

No.	Pathway	FDR	Enriched Genes
1	HOTAIR regulatory pathway	1.68 × 10^−3^	*CD44*, *MMP12*, *NFKBIA*
2	Role of Osteoclasts in rheumatoid arthritis	5.88 × 10^−3^	*MMP12*, *ADAM9*, *NFKBIA*
3	Collagen degradation	6.92 × 10^−3^	*MMP12*, *ADAM9*
4	Degradation of the extracellular matrix	8.35 × 10^−3^	*CD44*, *MMP12*
5	ABC-family protein-mediated transport	8.83 × 10^−3^	*PSME3*, *APOA1*
6	Post-translational protein phosphorylation	8.83 × 10^−3^	*APOA1*, *SPARCL1*
7	Regulation of IGF transport and uptake by IGFBPs	8.83 × 10^−3^	*APOA1*, *SPARCL1*
8	TCR signaling	8.83 × 10^−3^	*NFKBIA*, *PSME3*
9	Interleukin-1 family signaling	8.83 × 10^−3^	*NFKBIA*, *PSME3*
10	Centrosomal KIAA0586 signaling pathway	8.83 × 10^−3^	*CD44*, *PSME3*

Analysis was performed using IPA. The FDR (false discovery rate) indicates the statistical significance of the enrichment.

**Table 4:** The IC_50_ values for compounds (**C1**, **C7**, **C9**, **C10**, and **C15**) against H1299 lung cancer cells.

Compound	IC_50_ (µM, 48 h)	IC_50_ (µM, 72 h)	IC_50_ (µM, 96 h)
**C1**	358.90	91.72	99.70
**C7**	197.51	48.11	71.83
**C9**	496.33	43.41	66.81
**C10**	622.50	65.51	65.34
**C15**	222.20	145.23	178.64

Experiments were carried out for 48, 72, and 96 h of treatment duration. Experiments were run in duplicate for at least three independent trials (*n* = 6). Standard deviation did not exceed 10%; μM: micromolar.

**Table 5:** Effect of MMP12 inhibitors on the gene expression of MMP12 and downstream targets in H1299 lung cancer cells.

Compound	Gene	Fold Change	% Change	Direction
**C1**	*CD44*	0.48	−52%	Dn
	*NFKBIA*	24.33	+2333%	Up
	*PSME3*	0.54	−46%	Dn
	*SPARCL1*	0.49	−51%	Dn
	*ADAM9*	123.60	+12,260%	Up
	*APOA1*	0.79	−21%	Dn
	*CCL15*	0.03	−97%	Dn
	*MMP12*	0.78	−22%	Dn
**C7**	*CD44*	0.23	−77%	Dn
	*NFKBIA*	25.19	+2419%	Up
	*PSME3*	0.66	−34%	Dn
	*SPARCL1*	0.56	−44%	Dn
	*ADAM9*	29.45	+28.45	Up
	*APOA1*	12.17	+1117	Up
	*CCL15*	0.05	−95%	Dn
	*MMP12*	0.15	−85%	Dn
**C9**	*CD44*	0.91	−9%	Dn
	*NFKBIA*	29.10	+2810%	Up
	*PSME3*	0.36	−64%	Dn
	*SPARCL1*	0.34	−66%	Dn
	*ADAM9*	111.04	+11,004%	Up
	*APOA1*	7.67	+667%	Up
	*CCL15*	0.05	−95%	Dn
	*MMP12*	0.23	−77%	Dn
**C10**	*CD44*	0.45	−55%	Dn
	*NFKBIA*	18.50	+1750%	Up
	*PSME3*	0.97	−3%	Dn
	*SPARCL1*	0.26	−74%	Dn
	*ADAM9*	267.80	+26,680%	Up
	*APOA1*	0.81	−19%	Dn
	*CCL15*	0.05	−95%	Dn
	*MMP12*	0.57	−43%	Dn
**C15**	*CD44*	0.68	−32%	Dn
	*NFKBIA*	28.34	+2734%	Up
	*PSME3*	0.52	−48%	Dn
	*SPARCL1*	0.79	−21%	Dn
	*ADAM9*	39.26	+3826%	Up
	*APOA1*	6.34	+534%	Up
	*CCL15*	0.03	−97%	Dn
	*MMP12*	0.20	−80%	Dn

Relative mRNA expression levels of MMP12 and selected downstream genes following treatment with compounds **C1**, **C7**, **C9**, **C10**, and **C15** at 0.1 IC_50_ in H1299 cells, as determined by real-time quantitative PCR. Data are expressed as fold change (2^−ΔΔCt^) relative to untreated cells and the corresponding percent inhibition compared to control. GAPDH was used as the internal housekeeping gene for normalization. The results reflect the average of three independent experiments (*n* = 2). Dn: downregulation; Up: upregulation.

**Table 6:** Key upstream regulators modulated by MMP12 inhibition, identified by IPA, and shared across multiple compounds.

No.	Ustream Regulator	Effect Direction	Z-Score (Range)	Compounds
1	MMP12	↑ NFKBIA, ↓ PSME3, ↓ SPARCL1	+1.73	**C1**, **C7**, **C9**, **C10**, **C15**
2	TXN	↓ APOA1, ↓ CD44, ↓ MMP12, ↑ NFKBIA	+2.00 (**C1**, **C10**) +1.00 (**C7**, **C15**)	**C1**, **C7**, **C10**, **C15**
3	ITCH	↑ ADAM9, ↓ CD44, ↓ MMP12, ↑ NFKBIA	−1.00	**C1**, **C7**, **C10**, **C15**
4	AKT family	↓ CD44, ↓ MMP12, ↑ NFKBIA	−1.73	**C1**, **C7**, **C10**, **C15**
5	MAP3K11	↓ CD44, ↓ MMP12, ↑ NFKBIA, ↓ PSME3	−1.00	**C1**, **C7**, **C10**, **C15**
6	MAP2K7	↓ CD44, ↓ MMP12, ↑ NFKBIA	−0.58	**C1**, **C7**, **C10**, **C15**
7	MAP3K14	↓ CD44, ↑ NFKBIA	0.00	**C1**, **C7**, **C10**, **C15**
8	ECSIT	↓ CD44, ↑ NFKBIA	0.00	**C7**, **C10**, **C15**
9	DGKH	↓ CD44, ↓ MMP12, ↑ NFKBIA	−0.58	**C1**, **C7**, **C10**, **C15**
10	SH3RF1	↓ CD44, ↓ MMP12, ↑ NFKBIA	−0.58	**C1**, **C7**, **C10**, **C15**
11	VEGFA	↓ CD44, ↓ MMP12	−1.41	**C1**
12	CRK/CRKL	↑ APOA1, ↓ CCL15, ↓ CD44, ↓ MMP12, ↑ NFKBIA	−2.24	**C9**
13	SMAD2/3/4 complex	↑ ADAM9, ↑ APOA1, ↓ CD44, ↓ MMP12, ↑ NFKBIA	+0.45	**C9**
14	EIF3H	↑ ADAM9, ↓ CD44, ↓ MMP12, ↑ NFKBIA, ↓ PSME3	+1.34	**C9**
15	NRG4	↑ ADAM9, ↑ APOA1, ↓ CD44, ↓ MMP12, ↑ NFKBIA, ↓ SPARCL1	−0.82	**C9**
16	ERBB family	↑ ADAM9, ↑ APOA1, ↓ CD44, ↓ MMP12, ↑ NFKBIA, ↓ SPARCL1	−0.82	**C9**
17	RHO-GDI	↑ ADAM9, ↑ APOA1, ↓ CD44, ↓ MMP12, ↑ NFKBIA	−1.34	**C9**
18	EGFR	↑ ADAM9, ↑ APOA1, ↓ CD44, ↓ MMP12, ↑ NFKBIA, ↓ SPARCL1	−0.82	**C9**
19	BHLH	↑ APOA1, ↓ CD44, ↓ MMP12, ↑ NFKBIA, ↓ SPARCL1	−0.45	**C9**
20	ERBB4/APOE complex	↑ ADAM9, ↑ APOA1, ↓ CD44, ↓ SPARCL1	+1.00	**C9**

The table lists key upstream regulators predicted from the gene expression changes induced by compounds **C1**, **C7**, **C9**, **C10**, and **C15**. It includes both regulators shared across multiple compounds and those unique to specific treatments (e.g., **C9**). Arrows (↑, ↓) denote the observed direction of change in target gene expression as upregulated and downregulated, respectively. The z-score predicts the activation state of the regulator, where a positive value indicates predicted activation and a negative value indicates predicted inhibition.

## Data Availability

Data that support the findings in this paper have been provided as Supplementary Figures and Tables that can be accessed through the journal’s website. The original contributions presented in this study are included in the article/Supplementary Materials. Further inquiries can be directed to the corresponding author.

## Supplementary Materials

The following supporting information can be downloaded at: https://www.mdpi.com/article/10.3390/ijms262411802/s1.

## Abbreviations

µM	Micromolar
ADAM9	A disintegrin and metalloproteinase domain-containing protein 9
AKT	Protein kinase B
AKT	Protein kinase B (PKB)
APOA1	Apolipoprotein A-I
CCL15	C-C motif chemokine ligand 15
CD44	CD44 antigen (cell adhesion receptor)
CDDI	Cortellis Drug Discovery Intelligence
cDNA	Complementary DNA
COPD	Chronic obstructive pulmonary disease
DMEM	Dulbecco’s Modified Eagle Medium
ECM	Extracellular matrix
EIF3H	Eukaryotic translation initiation factor 3 subunit H
ERBB family	Epidermal growth-factor receptor family (EGFR/ERBB1, ERBB2, ERBB3, ERBB4)
FBS	Fetal bovine serum
FHIT	Fragile histidine triad
GAPDH	Glyceraldehyde-3-phosphate dehydrogenase
HOTAIR	HOX transcript antisense RNA
IC50	Half-maximal inhibitory concentration
IGFBP	Insulin-like growth factor binding protein
IPA	Ingenuity pathway analysis
ITCH	E3 ubiquitin–protein ligase Itchy homolog
KRAS	Kirsten Rat Sarcoma Viral Oncogene Homolog
MAP3K11	Mitogen-activated protein kinase kinase kinase 11
MMP12	Matrix metalloproteinase-12
MMPs	Matrix metalloproteinases
MTT	3-(4,5-Dimethylthiazol-2-yl)-2,5-diphenyltetrazolium bromide
NFKBIA	NF-κB inhibitor alpha (IκBα)
NRG4	Neuregulin 4
NSCLC	Non-small cell lung cancer
PD	Pharmacodynamic
PI3K	Phosphoinositide 3-kinase
PSME3	Proteasome activator subunit 3 (PA28γ)
qPCR	Quantitative real-time polymerase chain reaction
RHO-GDI	Rho GDP-dissociation inhibitor
RPMI	Roswell Park Memorial Institute medium
SCLC	Small-cell lung cancer
SMAD	Small Mother Against Decapentaplegic
SPARCL1	SPARC-like protein 1 (Hevin)
SRC	Proto-oncogene tyrosine–protein kinase Src
TCR	T-cell receptor
TGF-β	Transforming growth factor-β
TXN	Thioredoxin
uPA	Urokinase-type plasminogen activator
uPAR	Urokinase-type plasminogen activator receptor

## References

[1] de Almeida L.G., Thode H., Eslambolchi Y., Chopra S., Young D., Gill S., Devel L., Dufour A. (2022). Matrix metalloproteinases: From molecular mechanisms to physiology, pathophysiology, and pharmacology. Pharmacol. Rev..

[2] Almutairi S., Kalloush H.M.d., Manoon N.A., Bardaweel S.K. (2023). Matrix metalloproteinases inhibitors in cancer treatment: An updated review (2013–2023). Molecules.

[3] Siddhartha R., Garg M. (2023). Interplay between extracellular matrix remodeling and angiogenesis in tumor ecosystem. Mol. Cancer Ther..

[4] Grillet B., Pereira R.V.S., Van Damme J., Abu El-Asrar A., Proost P., Opdenakker G. (2023). Matrix metalloproteinases in arthritis: Towards precision medicine. Nat. Rev. Rheumatol..

[5] Niland S., Riscanevo A.X., Eble J.A. (2021). Matrix metalloproteinases shape the tumor microenvironment in cancer progression. Int. J. Mol. Sci..

[6] Bassiouni W., Ali M.A., Schulz R. (2021). Multifunctional intracellular matrix metalloproteinases: Implications in disease. FEBS J..

[7] Christopoulou M.-E., Papakonstantinou E., Stolz D. (2023). Matrix metalloproteinases in chronic obstructive pulmonary disease. Int. J. Mol. Sci..

[8] Kwon M.J. (2023). Matrix metalloproteinases as therapeutic targets in breast cancer. Front. Oncol..

[9] Kandhwal M., Behl T., Singh S., Sharma N., Arora S., Bhatia S., Al-Harrasi A., Sachdeva M., Bungau S. (2022). Role of matrix metalloproteinase in wound healing. Am. J. Transl. Res..

[10] Fu K., Zheng X., Chen Y., Wu L., Yang Z., Chen X., Song W. (2022). Role of matrix metalloproteinases in diabetic foot ulcers: Potential therapeutic targets. Front. Pharmacol..

[11] Lee H.S., Kim W.J. (2022). The role of matrix metalloproteinase in inflammation with a focus on infectious diseases. Int. J. Mol. Sci..

[12] Mustafa S., Koran S., AlOmair L. (2022). Insights into the role of matrix metalloproteinases in cancer and its various therapeutic aspects: A review. Front. Mol. Biosci..

[13] Lin H., Xu P., Huang M. (2022). Structure-based molecular insights into matrix metalloproteinase inhibitors in cancer treatments. Future Med. Chem..

[14] He L., Kang Q., Chan K.I., Zhang Y., Zhong Z., Tan W. (2023). The immunomodulatory role of matrix metalloproteinases in colitis-associated cancer. Front. Immunol..

[15] Pezeshkian Z., Nobili S., Peyravian N., Shojaee B., Nazari H., Soleimani H., Asadzadeh-Aghdaei H., Ashrafian Bonab M., Nazemalhosseini-Mojarad E., Mini E. (2021). Insights into the role of matrix metalloproteinases in precancerous conditions and in colorectal cancer. Cancers.

[16] Tamang J.S.D., Banerjee S., Baidya S.K., Das S., Ghosh B., Jha T., Adhikari N. (2024). Matrix metalloproteinase-12 (MMP-12) and its inhibitors: A mini-review. Eur. J. Med. Chem..

[17] Lenci E., Cosottini L., Trabocchi A. (2021). Novel matrix metalloproteinase inhibitors: An updated patent review (2014–2020). Expert Opin. Ther. Pat..

[18] Yi C., Liu J., Deng W., Luo C., Qi J., Chen M., Xu H. (2022). Macrophage elastase (MMP12) critically contributes to the development of subretinal fibrosis. J. Neuroinflammation.

[19] Aristorena M., Gallardo-Vara E., Vicen M., de Las Casas-Engel M., Ojeda-Fernandez L., Nieto C., Blanco F.J., Valbuena-Diez A.C., Botella L.M., Nachtigal P. (2019). MMP-12, secreted by pro-inflammatory macrophages, targets endoglin in human macrophages and endothelial cells. Int. J. Mol. Sci..

[20] Li G.-S., Tang Y.-X., Zhang W., Li J.-D., Huang H.-Q., Liu J., Fu Z.-W., He R.-Q., Kong J.-L., Zhou H.-F. (2024). MMP12 is a Potential Predictive and Prognostic Biomarker of Various Cancers Including Lung Adenocarcinoma. Cancer Control.

[21] Eriksson Ström J., Kebede Merid S., Linder R., Pourazar J., Lindberg A., Melén E., Behndig A.F. (2025). Airway MMP-12 and DNA methylation in COPD: An integrative approach. Respir. Res..

[22] Abdel-Hamid N.M., Abass S.A. (2021). Matrix metalloproteinase contribution in management of cancer proliferation, metastasis and drug targeting. Mol. Biol. Rep..

[23] Sharma R. (2022). Mapping of global, regional and national incidence, mortality and mortality-to-incidence ratio of lung cancer in 2020 and 2050. Int. J. Clin. Oncol..

[24] Zhang W., Li G.-S., Gan X.-Y., Huang Z.-G., He R.-Q., Huang H., Li D.-M., Tang Y.-L., Tang D., Zou W. (2023). MMP12 serves as an immune cell–related marker of disease status and prognosis in lung squamous cell carcinoma. PeerJ.

[25] Hung W.-Y., Lee W.-J., Cheng G.-Z., Tsai C.-H., Yang Y.-C., Lai T.-C., Chen J.-Q., Chung C.-L., Chang J.-H., Chien M.-H. (2021). Blocking MMP-12-modulated epithelial-mesenchymal transition by repurposing penfluridol restrains lung adenocarcinoma metastasis via uPA/uPAR/TGF-β/Akt pathway. Cell. Oncol..

[26] Noël A., Perveen Z., Xiao R., Hammond H., Le Donne V., Legendre K., Gartia M.R., Sahu S., Paulsen D.B., Penn A.L. (2021). Mmp12 is upregulated by in utero second-hand smoke exposures and is a key factor contributing to aggravated lung responses in adult emphysema, asthma, and lung cancer mouse models. Front. Physiol..

[27] Lv F., Wang J., Wu Y., Chen H., Shen X. (2015). Knockdown of MMP12 inhibits the growth and invasion of lung adenocarcinoma cells. Int. J. Immunopathol. Pharmacol..

[28] Mouton A.J., Gonzalez O.J.R., Kaminski A.R., Moore E.T., Lindsey M.L. (2018). Matrix metalloproteinase-12 as an endogenous resolution promoting factor following myocardial infarction. Pharmacol. Res..

[29] Quintero-Fabián S., Arreola R., Becerril-Villanueva E., Torres-Romero J.C., Arana-Argáez V., Lara-Riegos J., Ramírez-Camacho M.A., Alvarez-Sánchez M.E. (2019). Role of matrix metalloproteinases in angiogenesis and cancer. Front. Oncol..

[30] Zheng M. (2016). Classification and pathology of lung cancer. Surg. Oncol. Clin..

[31] Abumansour H., Abusara O.H., Khalil W., Abul-Futouh H., Ibrahim A.I., Harb M.K., Abulebdah D.H., Ismail W.H. (2024). Biological evaluation of levofloxacin and its thionated derivatives: Antioxidant activity, aldehyde dehydrogenase enzyme inhibition, and cytotoxicity on A549 cell line. Naunyn-Schmiedeberg’s Arch. Pharmacol..

[32] Gridelli C., Rossi A., Carbone D.P., Guarize J., Karachaliou N., Mok T., Petrella F., Spaggiari L., Rosell R. (2015). Non-small-cell lung cancer. Nat. Rev. Dis. Primers.

[33] Wang Z., Cai G., Zhu J., Wang J., Zhang Y. (2025). Treatment of advanced-stage non-small cell lung cancer: Current progress and a glimpse into the future. Mol. Clin. Oncol..

[34] Baldavira C.M., Prieto T.G., de Souza M.L.F., Qualiotto A.N., Velosa A.P.P., Teodoro W.R., Takagaki T., Ab’Saber A., Capelozzi V.L. (2025). Matrisome analysis of NSCLC unveils clinically-important cancer-associated extracellular matrix changes. Biochim. Biophys. Acta (BBA)-Mol. Basis Dis..

[35] Xue H., Fan Y., Li Y., Zhao Q., Zhang X., Zhao P., Liu Z. (2025). Tumor-infiltrating lymphocytes in NSCLC: From immune surveillance to immunotherapy. Front. Immunol..

[36] Ibrahim A.I., Abul-Futouh H., Bourghli L.M., Abu-Sini M., Sunoqrot S., Ikhmais B., Jha V., Sarayrah Q., Abulebdah D.H., Ismail W.H. (2022). Design and synthesis of thionated levofloxacin: Insights into a new generation of quinolones with potential therapeutic and analytical applications. Curr. Issues Mol. Biol..

[37] Chen L., Zhang X.-H., Mao Z.-J., Wang D., Zhang C.-Y., Chen H.-J., Wu Y.-L., Yang J.-J. (2025). Clinical outcomes and neuroendocrine features of transformed versus primary small-cell lung cancer. Lung Cancer.

[38] Yiotakis A., Dive V. (2008). Third-generation MMP inhibitors: Recent advances in the development of highly selective inhibitors. Cancer Degrad. Proteases Cancer Biol..

[39] Raeeszadeh-Sarmazdeh M., Do L.D., Hritz B.G. (2020). Metalloproteinases and their inhibitors: Potential for the development of new therapeutics. Cells.

[40] Almutairi S., Sabbah D.A., Sweidan K., Hajjo R., Bardaweel S.K. (2025). Identification and Biological Validation of MMP-12 Inhibitors Guided by Pharmacophore-Based Virtual Screening and Docking Studies. ACS Omega.

[41] Hajjo R., Momani E., Sabbah D.A., Baker N., Tropsha A. (2023). Identifying a causal link between prolactin signaling pathways and COVID-19 vaccine-induced menstrual changes. NPJ Vaccines.

[42] Hajjo R., Sabbah D.A., Bardaweel S.K., Tropsha A. (2021). Shedding the light on post-vaccine myocarditis and pericarditis in COVID-19 and non-COVID-19 vaccine recipients. Vaccines.

[43] Hajjo R., Grulke C.M., Golbraikh A., Setola V., Huang X.-P., Roth B.L., Tropsha A. (2010). Development, validation, and use of quantitative structure− activity relationship models of 5-Hydroxytryptamine (2B) receptor ligands to identify novel receptor binders and putative valvulopathic compounds among common drugs. J. Med. Chem..

[44] Colás-Algora N., Muñoz-Pinillos P., Cacho-Navas C., Avendaño-Ortiz J., de Rivas G., Barroso S., López-Collazo E., Millán J. (2023). Simultaneous targeting of IL-1–signaling and IL-6–trans-signaling preserves human pulmonary endothelial barrier function during a cytokine storm—Brief report. Arterioscler. Thromb. Vasc. Biol..

[45] Chiou S.-H., Tseng D., Reuben A., Mallajosyula V., Molina I.S., Conley S., Wilhelmy J., McSween A.M., Yang X., Nishimiya D. (2021). Global analysis of shared T cell specificities in human non-small cell lung cancer enables HLA inference and antigen discovery. Immunity.

[46] Marques P.I., Carlos N.B., Seixas S. (2025). The tumor microbiome composition of non-small cell lung carcinoma correlates with expression differences in genes related with immunity, proteolysis and the extracellular matrix remodeling. Next Res..

[47] Flores-García L.C., García-Castillo V., Pérez-Toledo E., Trujano-Camacho S., Millán-Catalán O., Pérez-Yepez E.A., Coronel-Hernández J., Rodríguez-Dorantes M., Jacobo-Herrera N., Pérez-Plasencia C. (2025). HOTAIR Participation in Glycolysis and Glutaminolysis Through Lactate and Glutamate Production in Colorectal Cancer. Cells.

[48] Garlanda C., Mantovani A. (2021). Interleukin-1 in tumor progression, therapy, and prevention. Cancer Cell.

[49] Gong L., Geng L., Hou W., Xianyu J., Wang X. (2025). Simultaneous Detection of Calpain-2 and Matrix Metalloproteinase-12 in Non-Small Cell Lung Cancer Early Diagnosis. Sens. Actuators B Chem..

[50] Krämer A., Green J., Pollard J., Tugendreich S. (2014). Causal analysis approaches in ingenuity pathway analysis. Bioinformatics.

[51] Sternlicht M.D., Werb Z. (2001). How matrix metalloproteinases regulate cell behavior. Annu. Rev. Cell Dev. Biol..

[52] Mohan S., Thompson G.R., Amaar Y.G., Hathaway G., Tschesche H., Baylink D.J. (2002). ADAM-9 is an insulin-like growth factor binding protein-5 protease produced and secreted by human osteoblasts. Biochemistry.

[53] Go C.D., Knight J.D., Rajasekharan A., Rathod B., Hesketh G.G., Abe K.T., Youn J.-Y., Samavarchi-Tehrani P., Zhang H., Zhu L.Y. (2021). A proximity-dependent biotinylation map of a human cell. Nature.

[54] Yang Y., Iwanaga K., Raso M.G., Wislez M., Hanna A.E., Wieder E.D., Molldrem J.J., Wistuba I.I., Powis G., Demayo F.J. (2008). Phosphatidylinositol 3-kinase mediates bronchioalveolar stem cell expansion in mouse models of oncogenic K-ras-induced lung cancer. PLoS ONE.

[55] Kubota Y., Tanaka T., Kitanaka A., Ohnishi H., Okutani Y., Waki M., Ishida T., Kamano H. (2001). Src transduces erythropoietin-induced differentiation signals through phosphatidylinositol 3-kinase. EMBO J..

[56] Ehrhardt C., Ludwig S. (2009). A new player in a deadly game: Influenza viruses and the PI3K/Akt signalling pathway. Cell. Microbiol..

[57] Bernstein D., Fajardo G., Zhao M. (2011). The role of β-adrenergic receptors in heart failure: Differential regulation of cardiotoxicity and cardioprotection. Prog. Pediatr. Cardiol..

[58] Ohkawa Y., Momota H., Kato A., Hashimoto N., Tsuda Y., Kotani N., Honke K., Suzumura A., Furukawa K., Ohmi Y. (2015). Ganglioside GD3 enhances invasiveness of gliomas by forming a complex with platelet-derived growth factor receptor α and yes kinase. J. Biol. Chem..

[59] Malisan F., Franchi L., Tomassini B., Ventura N., Condò I., Rippo M.R., Rufini A., Liberati L., Nachtigall C., Kniep B. (2002). Acetylation suppresses the proapoptotic activity of GD3 ganglioside. J. Exp. Med..

[60] Rippo M.R., Malisan F., Rayagnan L., Tomassini B., Condo I., Costantini P., Susin S.A., Rufini A., Todaro M., Kroemer G. (2000). GD3 ganglioside directly targets mitochondria in a bcl-2-controlled fashion. FASEB J..

[61] Duxbury M.S., Ito H., Zinner M.J., Ashley S.W., Whang E.E. (2004). Inhibition of SRC tyrosine kinase impairs inherent and acquired gemcitabine resistance in human pancreatic adenocarcinoma cells. Clin. Cancer Res..

[62] Miyamoto-Sato E., Fujimori S., Ishizaka M., Hirai N., Masuoka K., Saito R., Ozawa Y., Hino K., Washio T., Tomita M. (2010). A comprehensive resource of interacting protein regions for refining human transcription factor networks. PLoS ONE.

[63] Lee I.Y., Lim J.M., Cho H., Kim E., Kim Y., Oh H.-K., Yang W.S., Roh K.-H., Park H.W., Mo J.-S. (2019). MST1 negatively regulates TNFα-induced NF-κB signaling through modulating LUBAC activity. Mol. Cell.

[64] Avruch J., Praskova M., Ortiz-Vega S., Liu M., Zhang X.F. (2006). Nore1 and RASSF1 regulation of cell proliferation and of the MST1/2 kinases. Methods Enzymol..

[65] Tsuruta F., Takebe A., Haratake K., Kanemori Y., Kim J., Endo T., Kigoshi Y., Fukuda T., Miyahara H., Ebina M. (2016). SCFFbl12 increases p21Waf1/Cip1 expression level through atypical ubiquitin chain synthesis. Mol. Cell. Biol..

[66] Leontieva O.V., Blagosklonny M.V. (2013). CDK4/6-inhibiting drug substitutes for p21 and p16 in senescence: Duration of cell cycle arrest and MTOR activity determine geroconversion. Cell Cycle.

[67] Nishiwaki E., Turner S.L., Harju S., Miyazaki S., Kashiwagi M., Koh J., Serizawa H. (2000). Regulation of CDK7–Carboxyl-Terminal Domain Kinase Activity by the Tumor Suppressor p16INK4A Contributes to Cell Cycle Regulation. Mol. Cell. Biol..

[68] Pekarsky Y., Garrison P.N., Palamarchuk A., Zanesi N., Aqeilan R.I., Huebner K., Barnes L.D., Croce C.M. (2004). Fhit is a physiological target of the protein kinase Src. Proc. Natl. Acad. Sci. USA.

[69] Roz L., Gramegna M., Ishii H., Croce C.M., Sozzi G. (2002). Restoration of fragile histidine triad (FHIT) expression induces apoptosis and suppresses tumorigenicity in lung and cervical cancer cell lines. Proc. Natl. Acad. Sci. USA.

[70] Sevignani C., Calin G.A., Cesari R., Sarti M., Ishii H., Yendamuri S., Vecchione A., Trapasso F., Croce C.M. (2003). Restoration of fragile histidine triad (FHIT) expression induces apoptosis and suppresses tumorigenicity in breast cancer cell lines. Cancer Res..

[71] Pang N., Lin Z., Wang X., Xu L., Xu X., Huang R., Li X., Li X., Li J. (2020). Endothelial cell-derived CCL15 mediates the transmigration of fibrocytes through the CCL15-CCR1 axis in vitro. Mol. Med. Rep..

[72] Clarivate Cortellis Drug Discovery Intelligence. https://clarivate.com/life-sciences-healthcare/research-development/discovery-development/cortellis-pre-clinical-intelligence/.

[73] Metacore C. (2025). MetaCore™ Pathway Analysis Software.

[74] HGNC HGNC: HUGO Gene Nomenclature Committee. https://www.genenames.org/.

[75] Shannon P., Markiel A., Ozier O., Baliga N.S., Wang J.T., Ramage D., Amin N., Schwikowski B., Ideker T. (2024). Cytoscape: An Open-Source Platform for Visualizing Complex Networks.

[76] QIAGEN (2025). QIAGEN Ingenuity Pathway Analysis (IPA).

[77] Hajjo R., Sabbah D.A., Tropsha A. (2022). Analyzing the systems biology effects of COVID-19 mRNA vaccines to assess their safety and putative side effects. Pathogens.

[78] Hajjo R., Setola V., Roth B.L., Tropsha A. (2012). Chemocentric informatics approach to drug discovery: Identification and experimental validation of selective estrogen receptor modulators as ligands of 5-hydroxytryptamine-6 receptors and as potential cognition enhancers. J. Med. Chem..

[79] Oliveros J.C. Venny: An Interactive Tool for Comparing Lists with Venn’s Diagrams. https://bioinformatics.psb.ugent.be/webtools/Venn/.

[80] Abdullah A.H., Alarareh A.K., Al-Sha’er M.A., Habashneh A.Y., Awwadi F.F., Bardaweel S.K. (2024). Docking, synthesis, and anticancer assessment of novel quinoline-amidrazone hybrids. Pharmacia.

[81] Bardaweel S.K., AlOmari R., Hajjo R. (2024). Integrating computational and experimental chemical biology revealed variable anticancer activities of phosphodiesterase isoenzyme 5 inhibitors (PDE5i) in lung cancer. RSC Med. Chem..

[82] Rasband W. (2007). ImageJ, US National Institutes of Health. http://rsb.info.nih.gov/ij/(1997-2007).

[83] Bardaweel S.K., Abu Sneineh B., Hajjo R., Abu Khalaf R. (2025). DPP4 inhibitors as a novel therapeutic strategy in colorectal cancer: Integrating network biology and experimental insights. PLoS ONE.

[84] Bardaweel S.K., Jaradat E., Hajjo R., AlJarrah H. (2025). Unraveling the Anticancer Potential of SSRIs in Prostate Cancer by Combining Computational Systems Biology and In Vitro Analyses. ACS Omega.

[85] Bardaweel S.K., Al-Salamat H., Hajjo R., Sabbah D., Almutairi S. (2024). Unveiling the intricacies of monoamine oxidase-A (MAO-A) inhibition in colorectal cancer: Computational systems biology, expression patterns, and the anticancer therapeutic potential. ACS Omega.

[86] Sweidan K., Elfadel H., Sabbah D.A., Bardaweel S.K., Hajjo R., Anjum S., Sinoj J., Nair V.A., Abu-Gharbieh E., El-Huneidi W. (2022). Novel derivatives of 4,6-Dihydroxy-2-quinolone-3-carboxamides as potential PI3Kα inhibitors. ChemistrySelect.

