# Supplementary material for: Molecular and System-Level Characterization of MMP12 Suppression in Lung Cancer: A Combined Bioinformatics and Molecular Approach
Source: Int J Mol Sci. 2025 Dec 6;26(24):11802. doi: 10.3390/ijms262411802 (PMC12732581; doi:10.3390/ijms262411802)
Supplement: Supplementary file 1 [file ijms-26-11802-s001.zip › ijms-4012078-supplementary.pdf]

Supplementary File

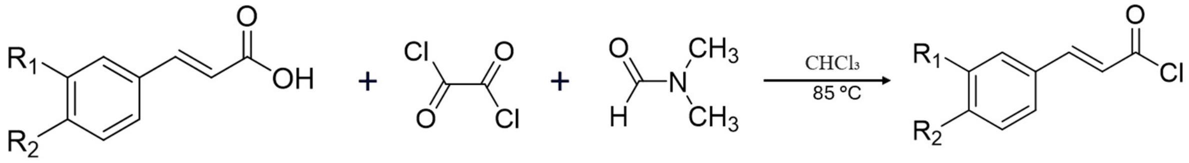

(**M1**), R_1= H, R_2= Cl (**M2**), R_1= H, R_2= Cl

Scheme S1 Synthesis of Compound M2
Reagents and conditions: Oxalyl chloride, DMF, ice bath, then heated in an oil bath at 85°C for 24 h.

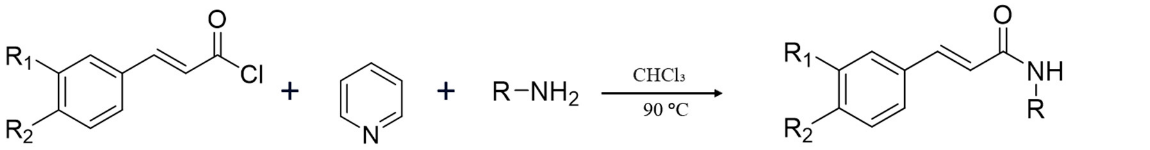

(**M2**), R_1= H, R_2= Cl (R)= **a-f**

(**C1**), R_1= H, R_2= Cl, R= a (**C4**), R_1= H, R_2= Cl, R= d
(**C2**), R_1= H, R_2= Cl, R= b (**C5**), R_1= H, R_2= Cl, R= e
(**C3**), R_1= H, R_2= Cl, R= c (**C6**), R_1= H, R_2= Cl, R= f

Scheme S2 Synthesis of **C1-C6**.
Reagents and conditions: $CHCl_3$, 90°C, pyridine, 24 h.

Figures S1(-A to -C). The ^{1}H NMR, ^{13}C NMR, and HRMS spectra of **C1**

- **(E)-*N*-(4-benzoylphenyl)-3-(4-chlorophenyl)acrylamide** (**C1**)

Off-white solid, yield= **49%,** M.P. =191-192 C°,R_f = 0.7 (EtOAc: n-hexane 1:3, *v* / *v*). **^{1}H NMR** (500 MHz, $CDCl_3$) δ 7.86 (d, *J* = 8.2 Hz, 2H, H3,H5), 7.81 – 7.72 (m, 5H, H2,H6+H9,H13, H2”), 7.64 (s, 1H, NH-amide), 7.59 (t, *J* = 7.4 Hz, 1H, H11), 7.48 (dd, *J* = 13.4, 7.5 Hz, 4H, H3’, H5’+ H10, H12), 7.37 (d, *J* = 8.1 Hz, 2H, H2’, H6’), 6.55 (d, *J* = 15.5 Hz, 1H, H1”). **^{13}C NMR** (126 MHz, $CDCl_3$) δ 195.81 (C7), 163.95 (Amide Carbon), 142.18 (C4), 142.05 (C2”), 137.94 (C8), 136.36 (C1), 133.36 (C4’), 133.00 (C1’), 132.44 (C11), 131.86 (C2,C6), 130.04 (C3’, C5’), 129.40 (C9, C13), 129.36 (C2’, C6’), 128.46 (C10, C12), 120.93 (C1”), 119.15 (C3, C5). **HRMS** (ESI) *m/z*: calcd for $C_{22}H_{17}ClNO_2$ $[M+H]^+$: 362.09423, found: 362.09431.

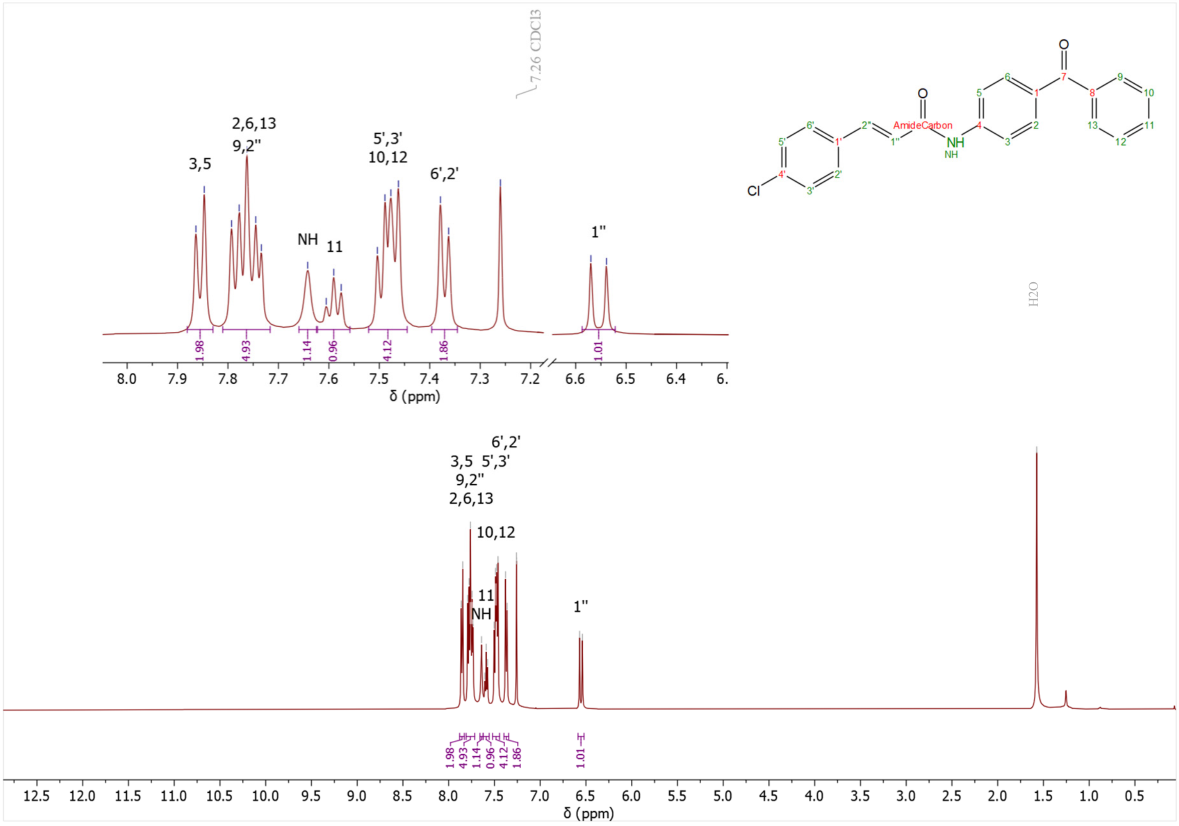

Figure S1-A. The ^{1}H NMR spectrum of compound **C1.** ^{1}H NMR instrument (500 MHz), solvent used is ($CDCL_3$).

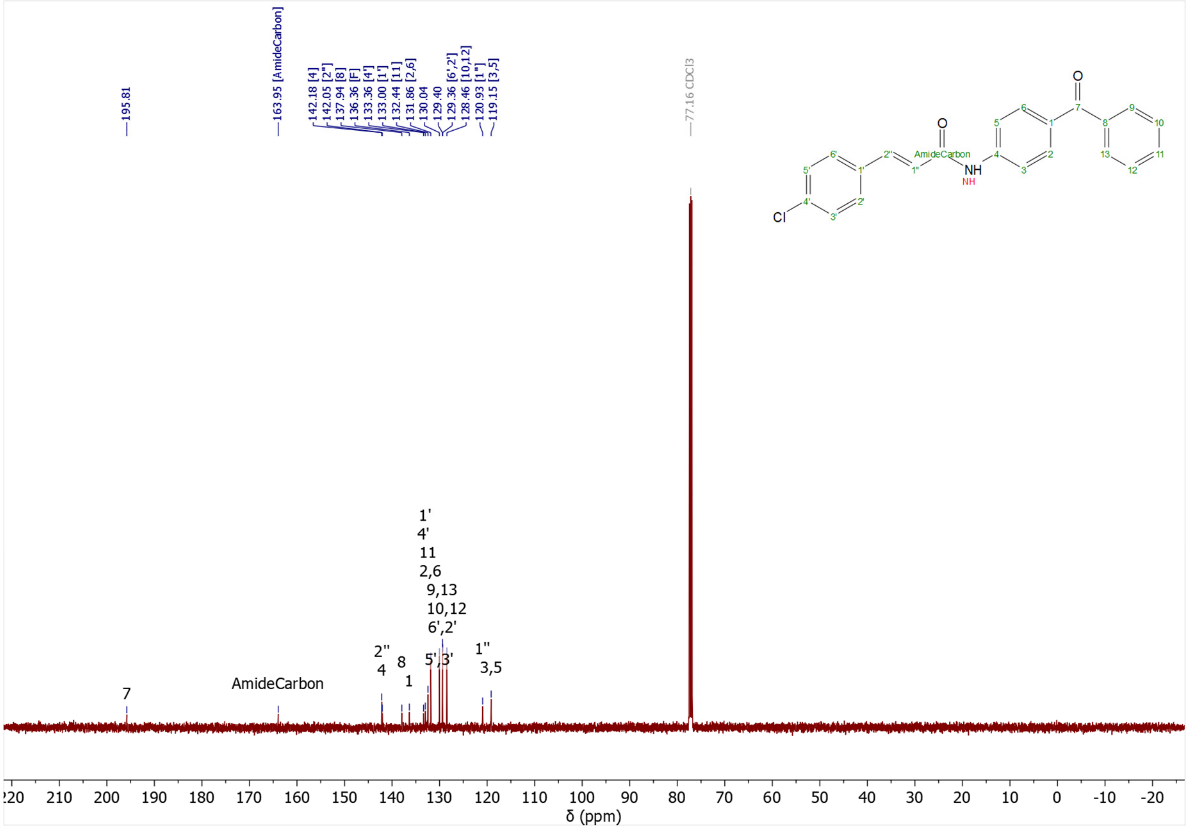

Figure S1-B. The ^{13}C NMR spectrum of compound **C1.** NMR instrument (500 MHz), solvent used is ($CDCL_3$)

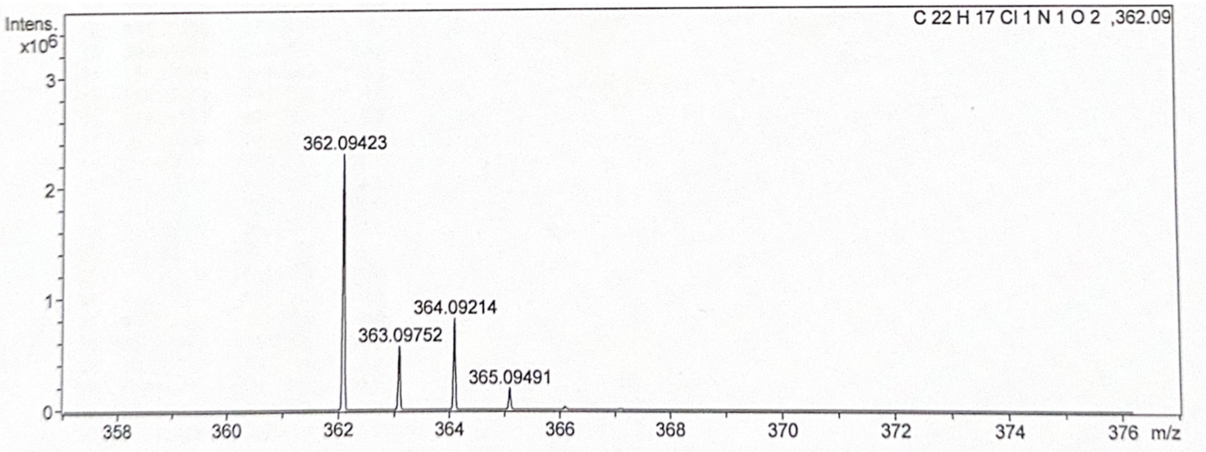

Figure S1-C. The HRMS (ESI) spectrum of compound **C1.**

Figures S2(-A to -C). The ^{1}H NMR, ^{13}C NMR, and HRMS spectra of **C2**

- **(E)-*N*-(3-benzoylphenyl)-3-(4-chlorophenyl)acrylamide** (**C2**)

Beige solid, yield= 55%, M.P. =139-141 C°,R_f = 0.53 (EtOAc: n-hexane 1:3, *v* / *v*). **^{1}H NMR** (500 MHz, DMSO) δ 8.60 (s, 1H, H6), 7.85 (d, *J* = 3.9 Hz, 1H, H4), 7.72 (d, *J* = 8.1 Hz, 3H, H2', H6' + H2), 7.58 (d, *J* = 16.0 Hz, 1H, H2''), 7.50 – 7.40 (m, 8H, H3 +H3', H5' + H9, H13 + H10, H12+ H11), 6.55 (d, *J* = 16.0 Hz, 1H, H1''). **^{13}C NMR** (126 MHz, DMSO) δ 167.43 (C7), 161.13 (Amide Carbon), 149.09 (C2''), 142.53 (C1), 134.71 (C4'+C5+C8), 133.23 (C1'+C11), 129.95 (C3 + C2', C6' + C9, C13), 128.94 (C3', C5' + C10, C12), 124.17 (C2+C4) 120.09 (C1''+ C6). **HRMS** (ESI) m/z: calcd for $C_{22}H_{16}Cl_1N_1O_2$ [M+Na]+: 384.07618, found: 384.07618

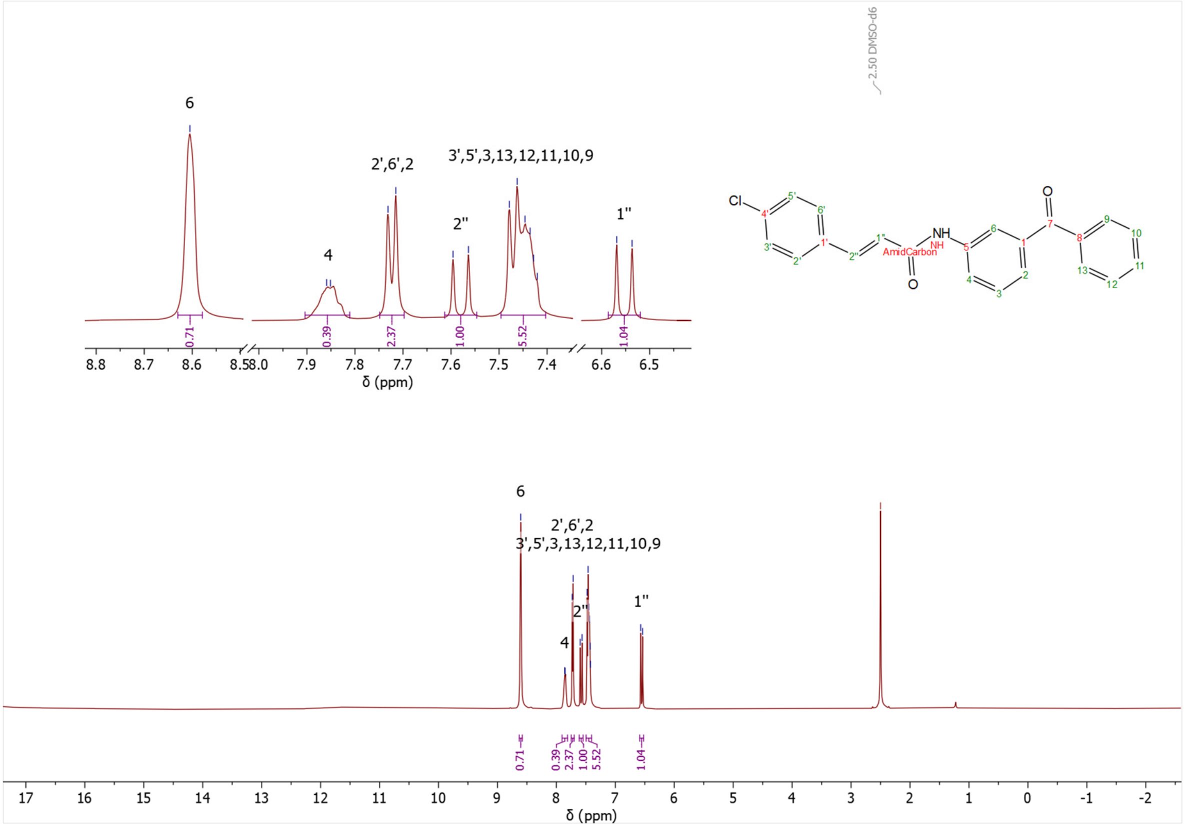

Figure S2-A. The ^{1}H NMR spectrum of compound **C2.** ^{1}H NMR instrument (500 MHz), solvent used is (DMSO-*d6*).

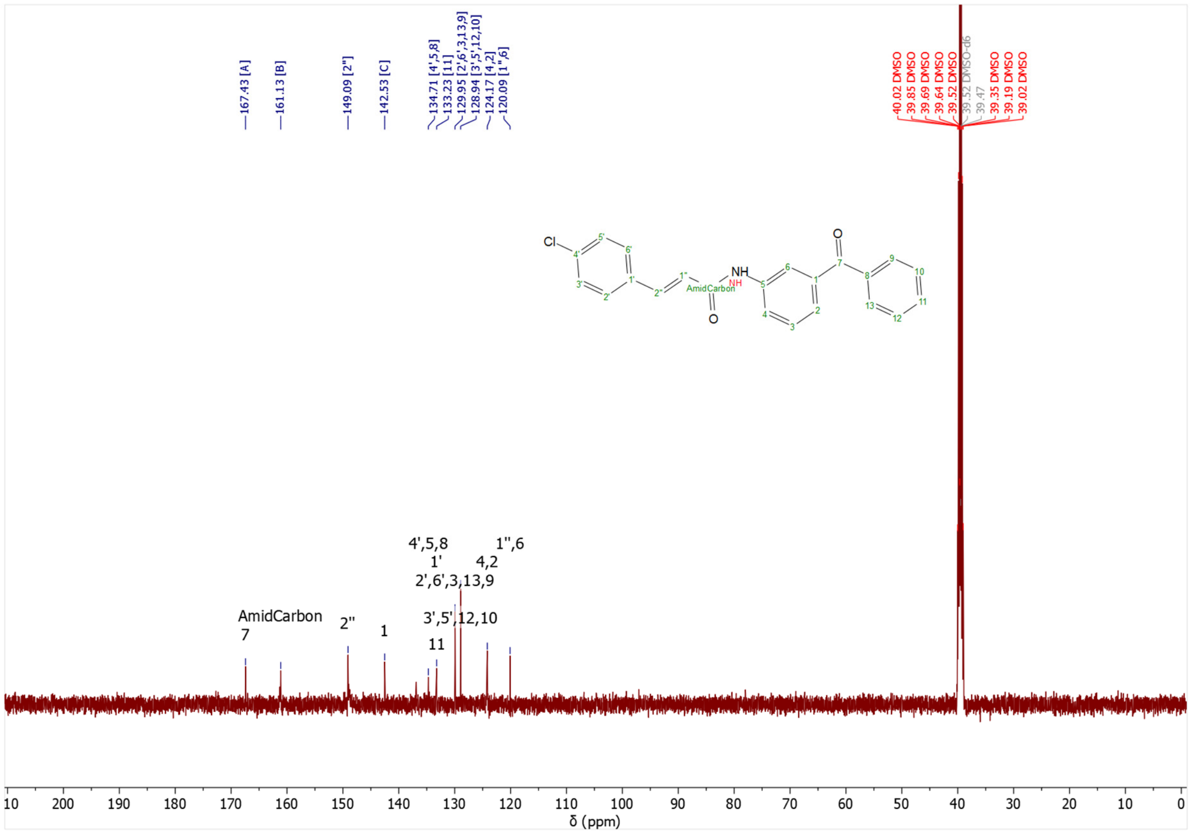

Figure S2-B. The ^{13}C NMR spectrum of compound **C2.** NMR instrument (500 MHz), solvent used is (DMSO-*d6*)

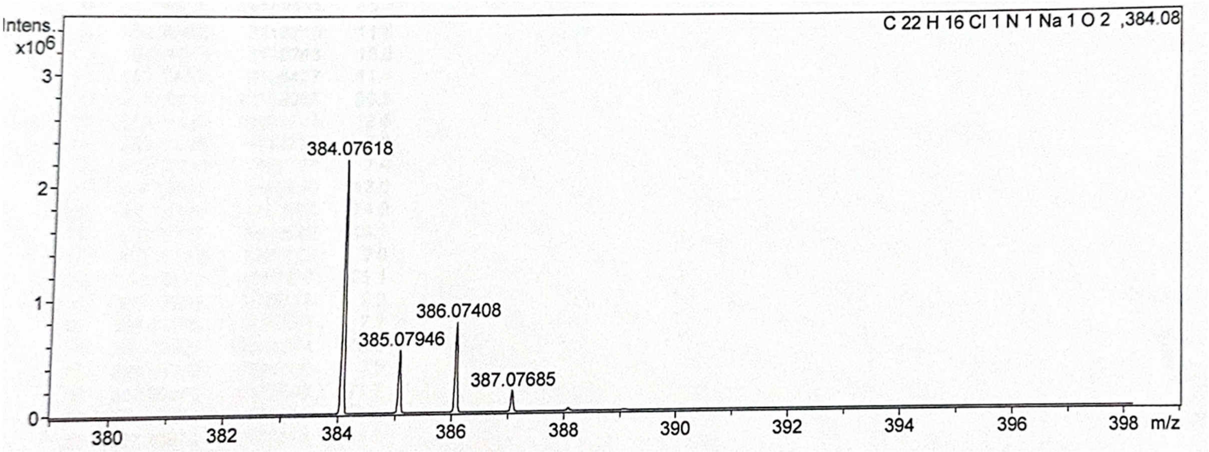

Figure S2-C. The HRMS (ESI) spectrum of compound **C2.**

Figures S3-A and -B. The ^{1}H NMR, ^{13}C NMR, and HRMS spectra of **C3**

- **(E)-*N*-(2-benzoyl-4-chlorophenyl)-3-(4-chlorophenyl)acrylamide** (**C3**)

Beige solid, yield= 40 %, M.P. =198-199 C°,R_f = 0.58 (EtOAc: n-hexane 1:3, *v* / *v*). **^{1}H NMR** (500 MHz, DMSO) δ 7.66 – 7.49 (m, 5H, H9,H13 + H10, H12 + H11), 7.49 – 7.39 (m, 3H, H2’, H6’ + H4), 7.39 – 7.29 (d, 2H, H3’, H5’), 7.22 (d, *J* = 11.5 Hz, 1H, H2”), 7.11 (S, 1H, H6), 6.86 (d, *J* = 8.9 Hz, 1H, H3), 6.46 (d, *J* = 16.0 Hz, 1H, H1”). **^{13}C NMR** (126 MHz, DMSO) δ 197.63 (C7), 168.33 (Amid Carbon), 144.82 (C2”), 139.65 (C2+C8), 134.56 (C4’), 132.69 (C4+C11), 131.94 (C1’), 130.89 (C6), 129.50 (C9, C13), 129.01 (C3’, C5’), 128.93 (C2’, C6’), 128.66 (C1+C5+C10, C12), 119.40 (C3), 117.59 (C1”).

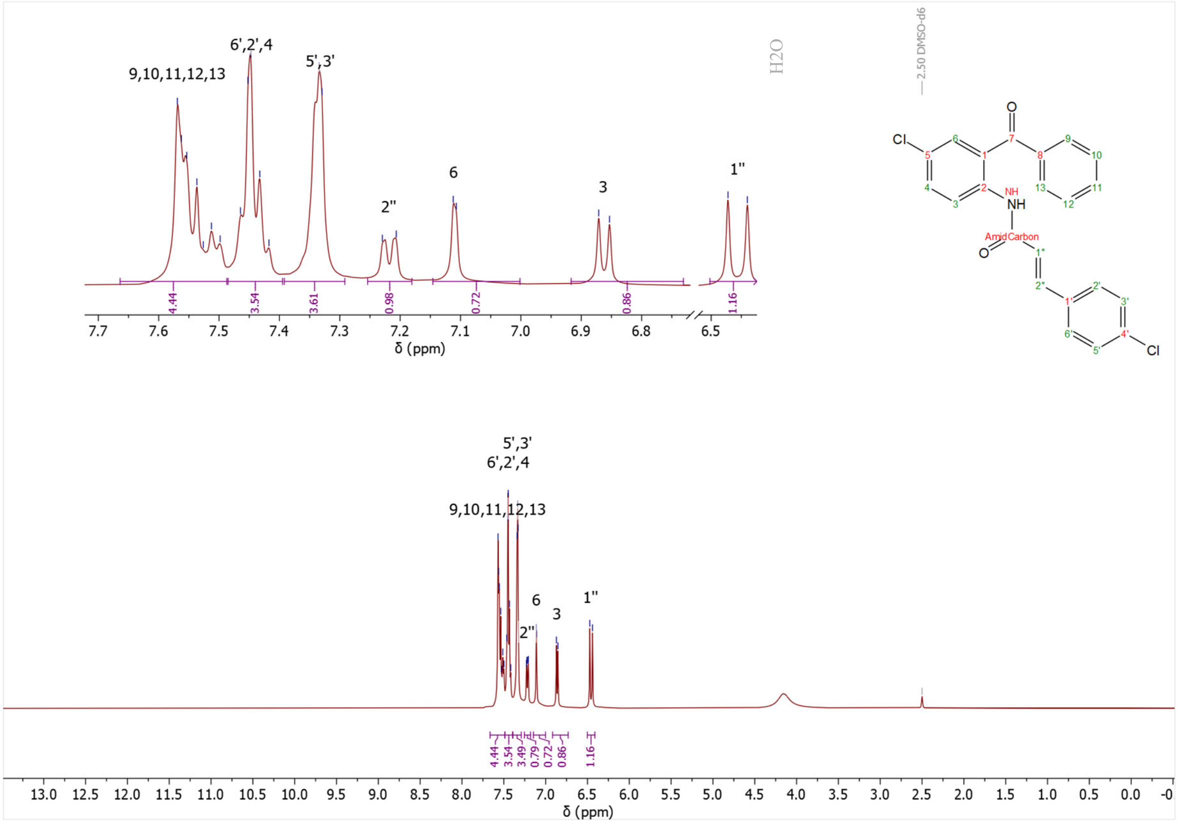

Figure S3-A. The ^{1}H NMR spectrum of compound **C3.** ^{1}H NMR instrument (500 MHz), solvent used is (DMSO-*d6*).

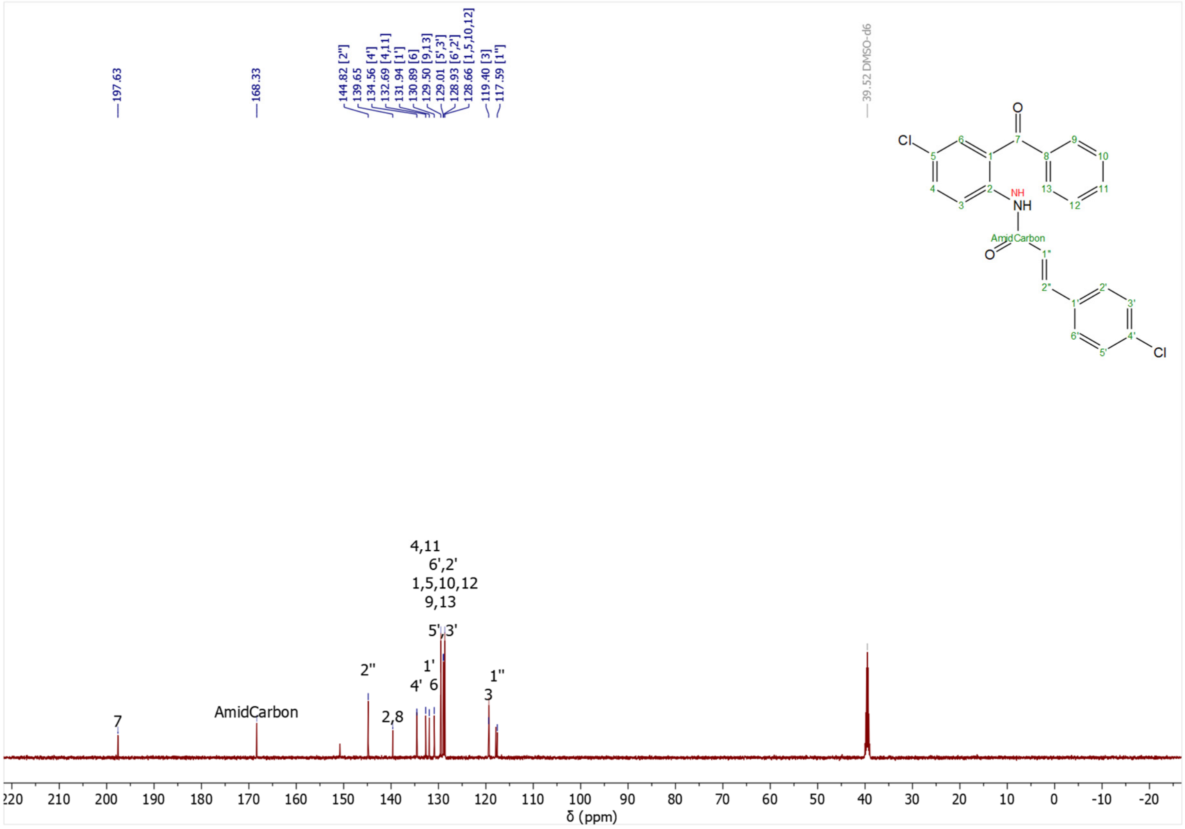

Figure S3-B. The ^{13}C NMR spectrum of compound **C3.** NMR instrument (500 MHz), solvent used is (DMSO-*d6*)

Figures S4-A and -B. The ^{1}H NMR, ^{13}C NMR, and HRMS spectra of **C4**

- **(E)-*N*-(2-benzoyl-4-methylphenyl)-3-(4-chlorophenyl)acrylamide (C4)**

Brown solid, yield= 45%, M.P. =159-160 C°,R_f = 0.55 (EtOAc: n-hexane 1:3, *v* / *v*). **^{1}H NMR** (500 MHz, DMSO) δ 7.59 (s, 1H, H3), 7.60 – 7.52 (m, 6H, (H2''+ H3', H5' +H10', H12' +H11)), 7.45 (d, *J* = 5.2 Hz, 1H, H6), 7.37 (d, *J* = 2.7 Hz, 4H, H2', H6' + H9, H13), 6.46 (d, *J* = 15.9 Hz, 2H, H1''+ H5), 2.16 (s, 1H, H(A)). **^{13}C NMR** (126 MHz, DMSO) δ 198.66 (C7), 168.70 (Amide Carbon), 146.02 (C2''), 145.24 (C2) 140.88 (C4+C8), 131.70 (C2', C6' +C11), 131.31 (C1'+ C6), 129.88 (C4'), 129.19 (C3', C5'), 129.10 (C9, C13), 128.99 (C1+ C10, C12), 119.52 (C1''+C3, C5), 22.11 (C(A)).

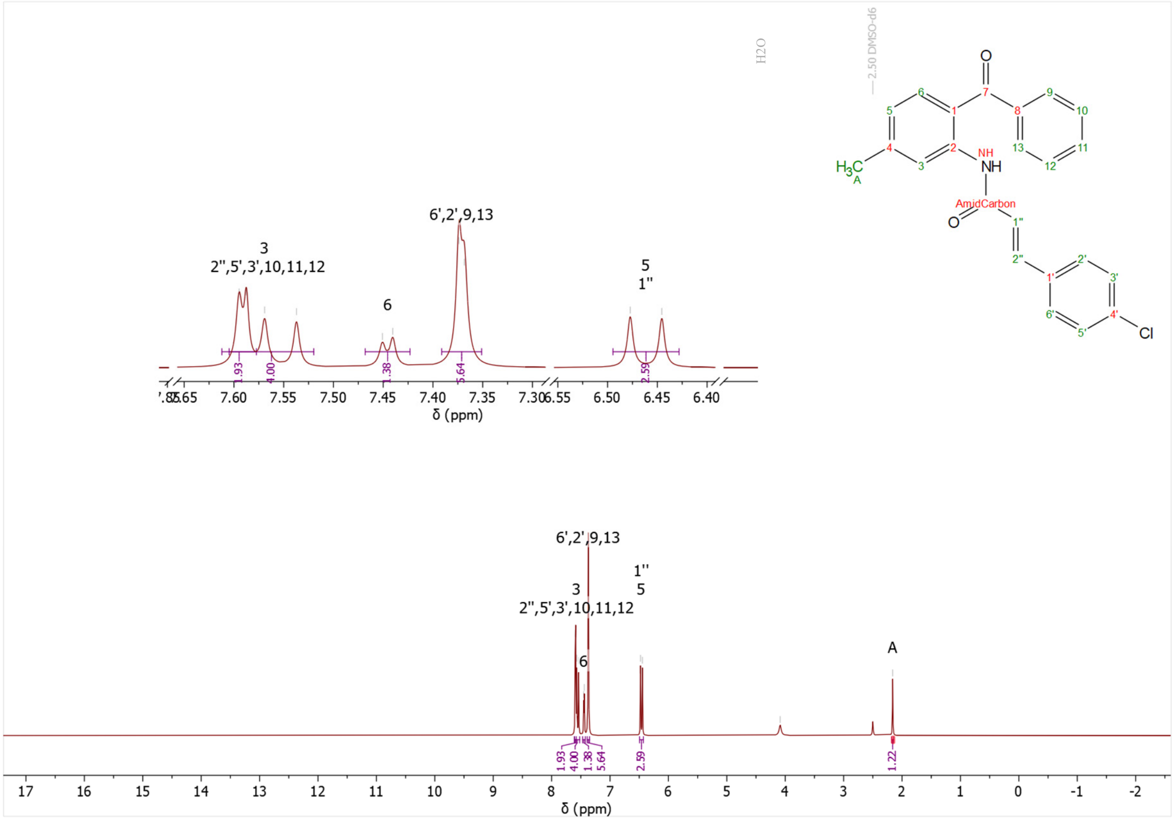

Figure S4-A. The ^{1}H NMR spectrum of compound **C4.** ^{1}H NMR instrument (500 MHz), solvent used is (DMSO-*d6*).

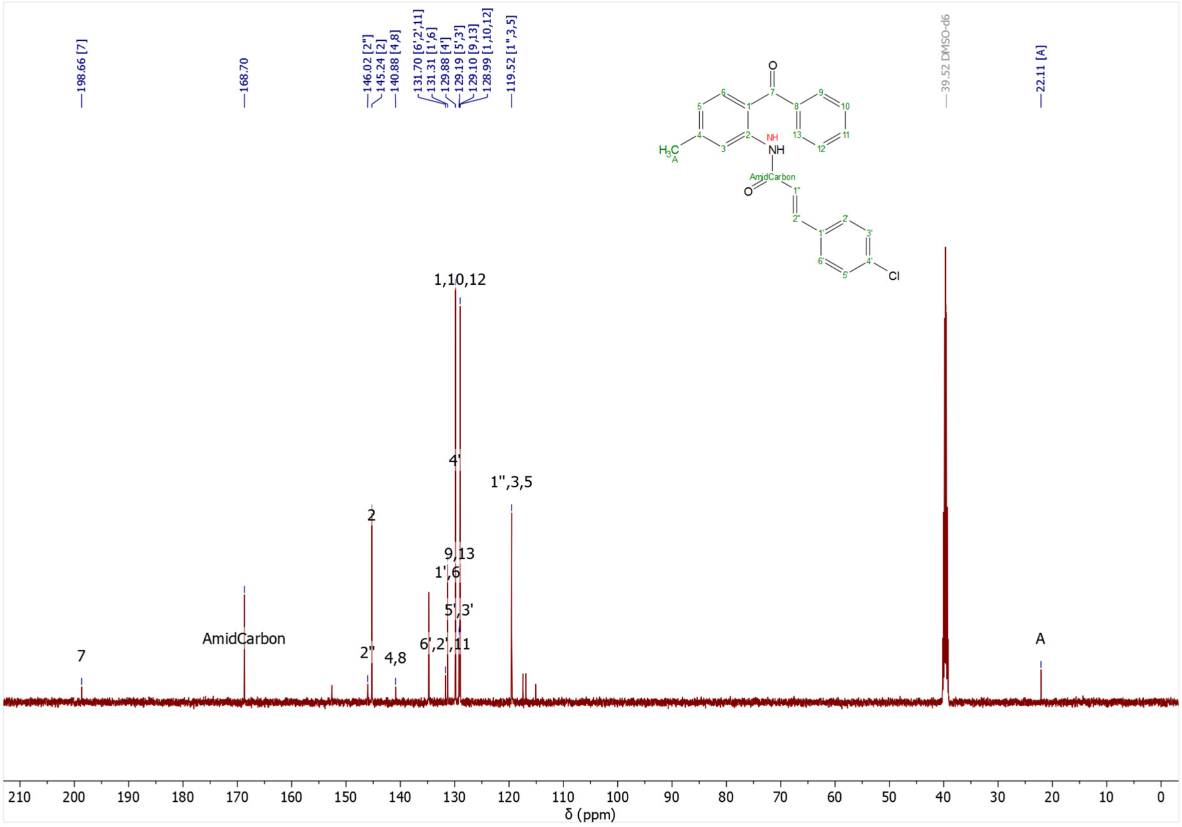

Figure S4-B. The ^{13}C NMR spectrum of compound **C4.** NMR instrument (500 MHz), solvent used is (DMSO-*d6*)

Figures S5-A and -B. The ^{1}H NMR, ^{13}C NMR, and HRMS spectra of **C5**

- **(E)-*N*-(2-benzoylphenyl)-3-(4-chlorophenyl)acrylamide (C5)**

Yellow solid, yield= 92%, M.P. =229-230 C°,R_f = 0.53 (EtOAc: n-hexane 1:3, *v* / *v*). **^{1}H NMR** (500 MHz, DMSO) δ 7.56 - 7.14 (m, 14H, H3+ H4+ H5+ H6+H9, H13 +H10, H12 + H11 + H2', H6' +H3', H5'), 6.81 (d, *J* = 8.4 Hz, 1H, H2''), 6.43 (d, *J* = 14.8 Hz, 1H, H1''). **^{13}C NMR** (126 MHz, DMSO) δ 199.16 (C7), 168.78 (Amide Carbon), 152.24 (C2), 145.21 (C2''), 140.49 (C1'), 135.26 (C8), 134.63 (C4'), 131.87 (C6+C11), 131.20 (C4), 129.77 (C1), 129.25 (C2',C6' +C3', C5'), 129.02 (C9, C13), 128.87 (C10, C12), 119.40 (C3, C5), 115.27 (C1'').

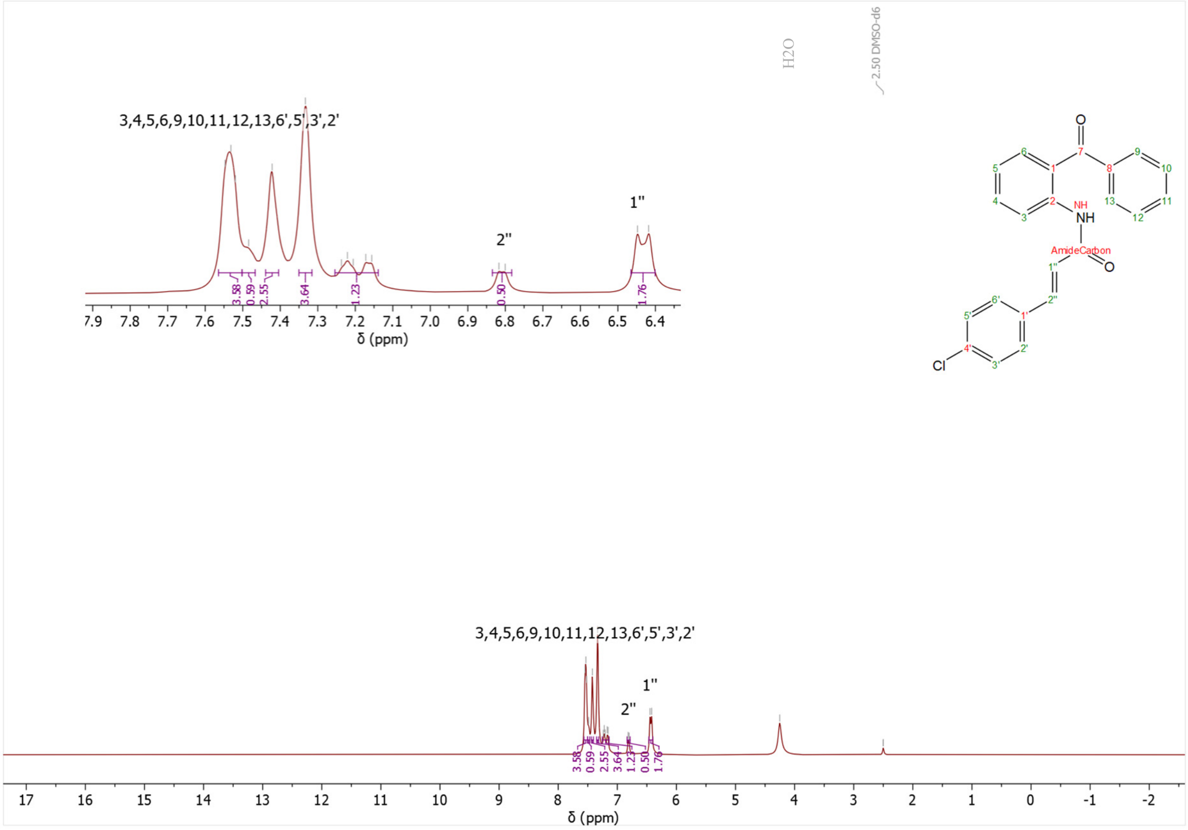

Figure S5-A. The ^{1}H NMR spectrum of compound **C5.** ^{1}H NMR instrument (500 MHz), solvent used is (DMSO-*d6*).

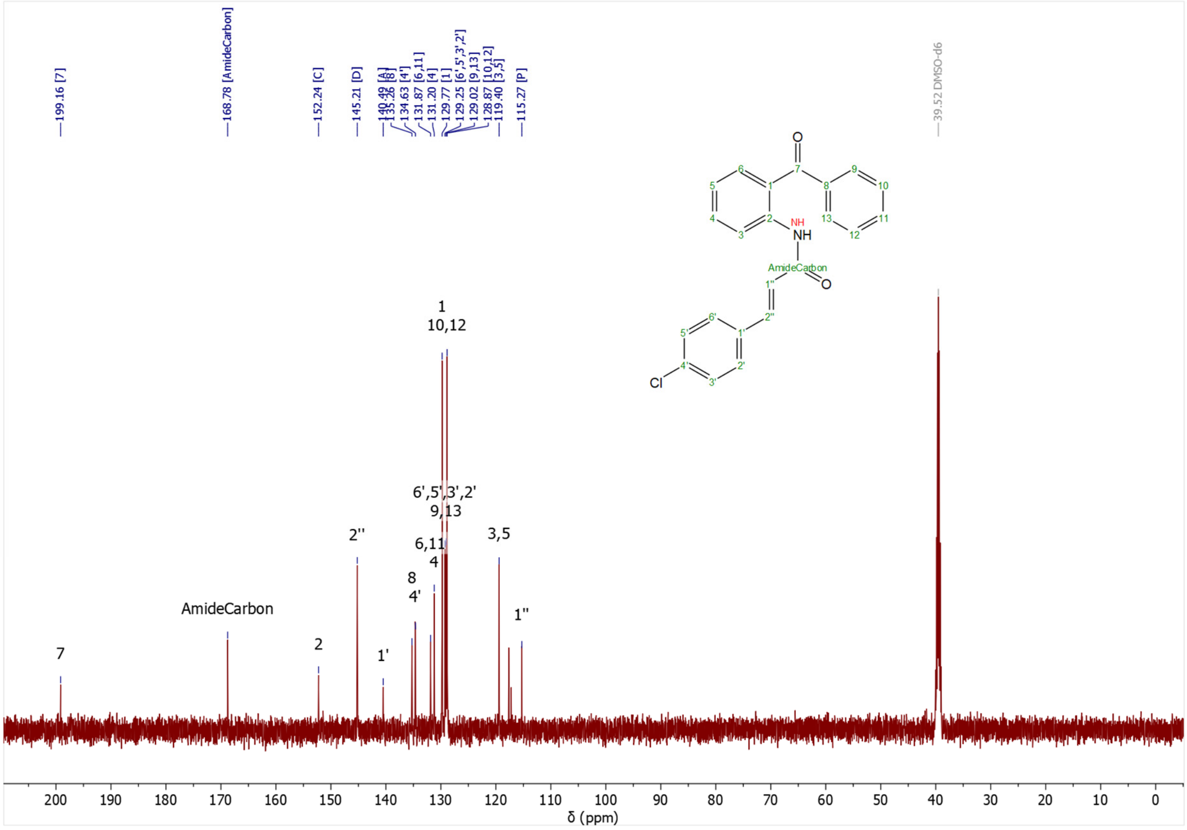

Figure S5-B. The ^{13}C NMR spectrum of compound **C5.** NMR instrument (500 MHz), solvent used is (DMSO-*d6*)

Figures S6-A and -B. The ^{1}H NMR, ^{13}C NMR, and HRMS spectra of **C6**

- **(E)-3-(4-chlorophenyl)-*N*-(2-(4-methylbenzoyl)phenyl)acrylamide (C6)**

Brown solid, yield= 40%, M.P. =212-215 C°, R_f = 0.56 (EtOAc: n-hexane 1:3, *v* / *v*). **^{1}H NMR** (500 MHz, DMSO) δ 7.59 (d, 2H, H2, H6)), 7.60 – 7.52 (m, 5H, (H2''+ H3', H5' + H10 + H11), 7.45 (d, *J* = 5.2 Hz, 1H, H9), 7.37 (d, *J* = 2.7 Hz, 5H, H2', H6'+ H3, H5+ H12), 6.46 (d, *J* = 15.9 Hz, 1H, H1''), 2.16 (s, 1H, (H(A)). **^{13}C NMR** (126 MHz, DMSO) δ 198.16 (C7), 168.20 (Amide Carbon), 145.52 (C2''), 144.74 (C4), 140.38 (C13), 131.20 (C1+ C1'+ C4'), 130.80 (C11), 129.37 (C2, C6 + C3, C5+ C2', C6' + C3',C5'), 128.69 (C9), 128.59 (C8), 128.49 (C10), 119.01 (C1'', C12), 21.60 (C(A)).

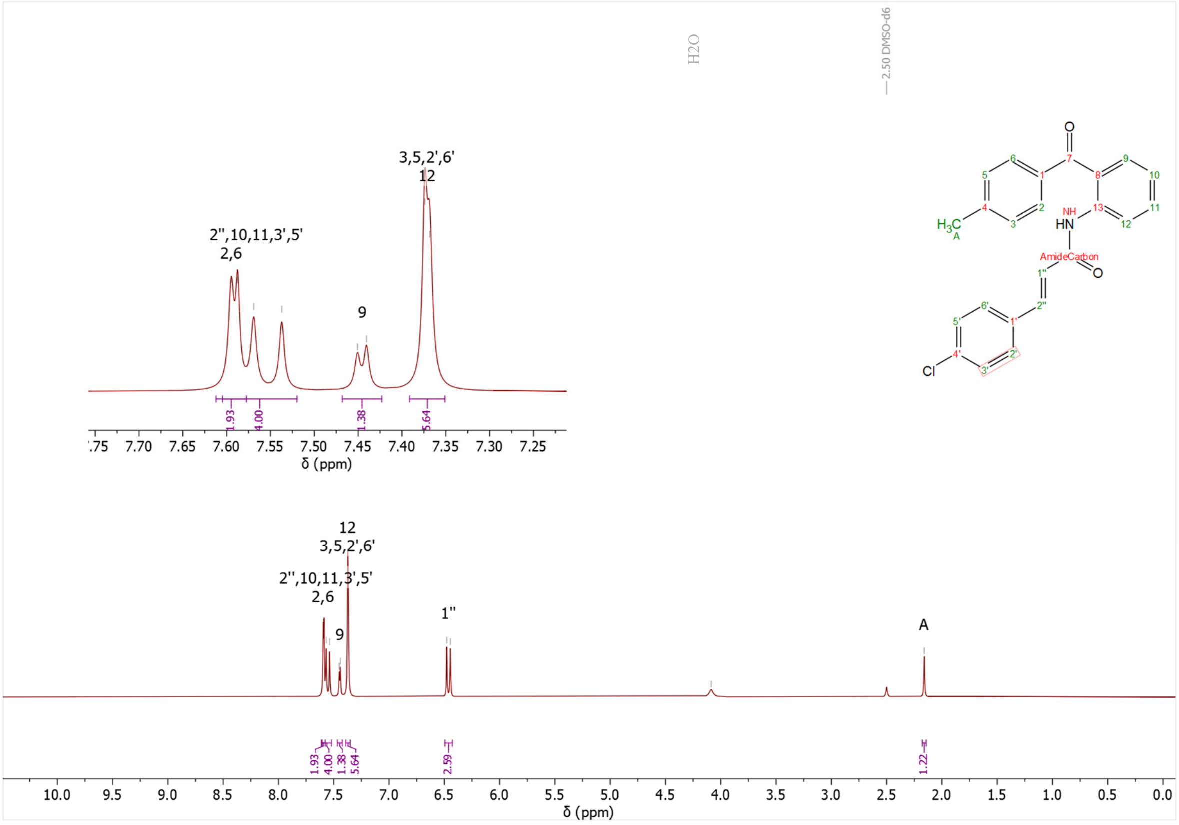

Figure S6-A. The ^{1}H NMR spectrum of compound **C6.** ^{1}H NMR instrument (500 MHz), solvent used is (DMSO-*d6*).

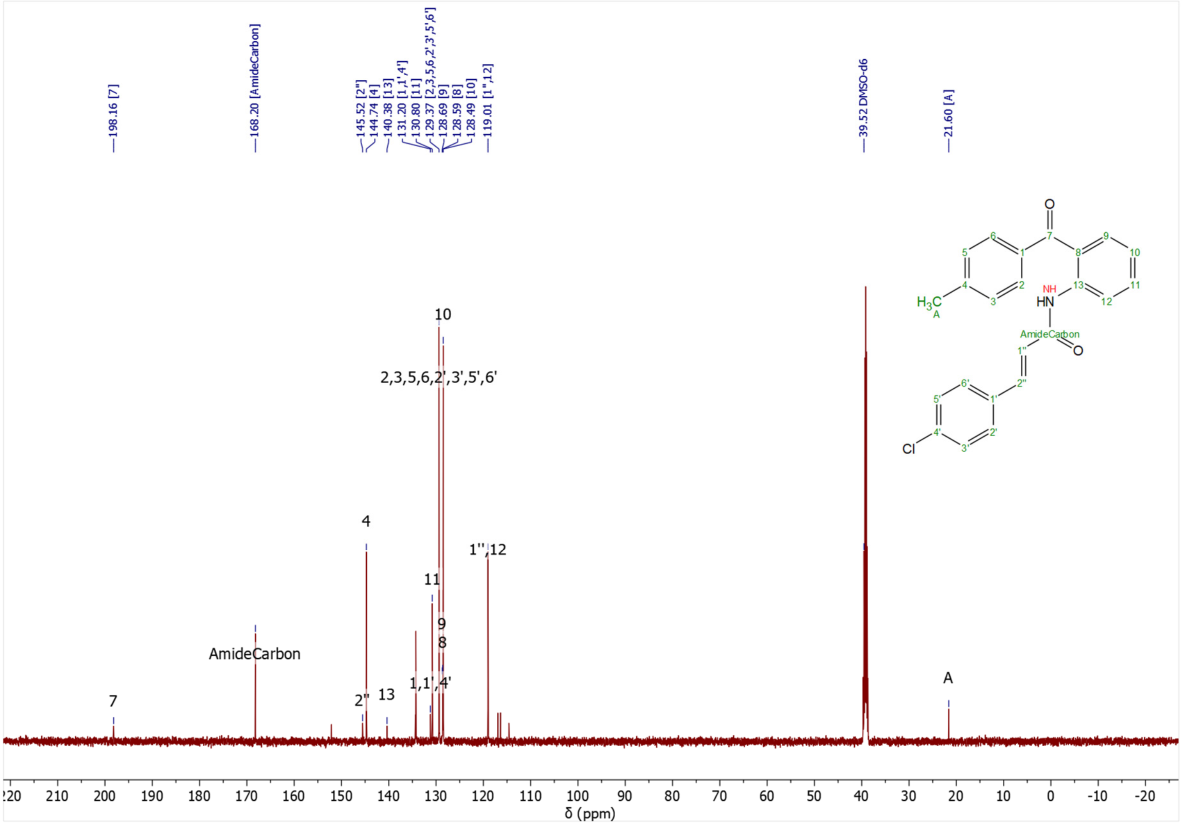

Figure S6-B. The ^{13}C NMR spectrum of compound **C6.** NMR instrument (500 MHz), solvent used is (DMSO-*d6*)

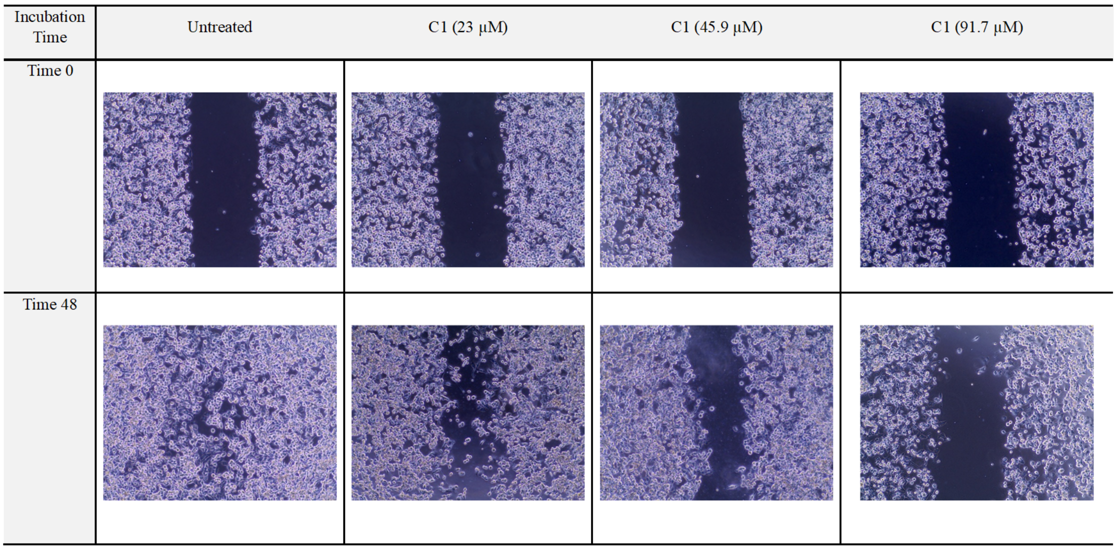

Figure S7. The effect of MMP-12 inhibitor (**C1**) treatment on H1299 cells migration.

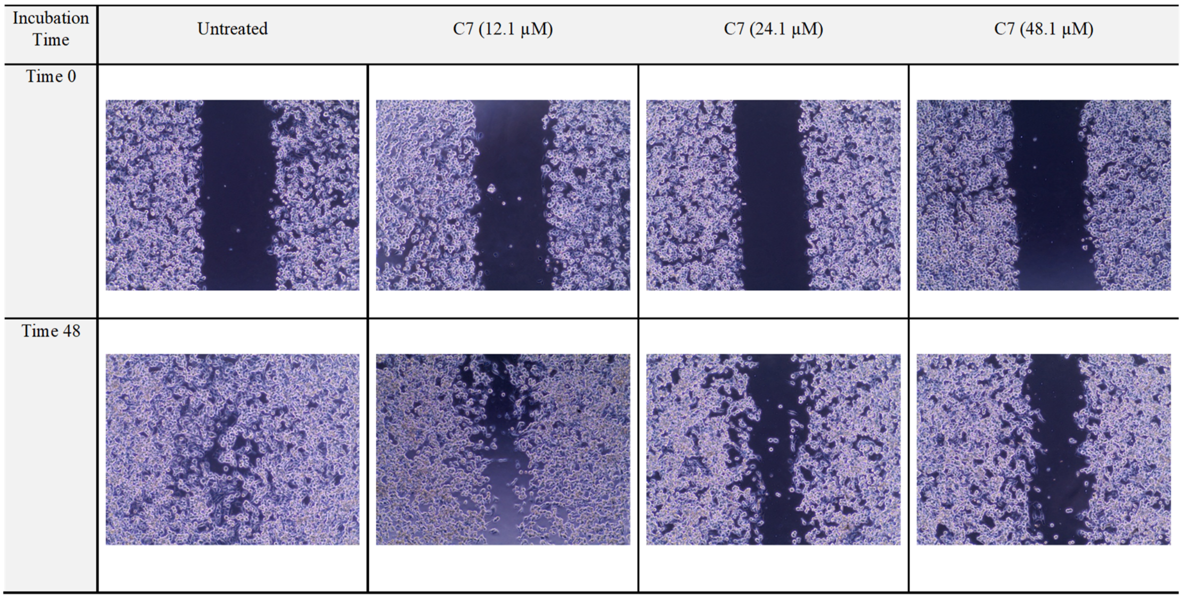

Figure S8. The effect of MMP-12 inhibitor (**C7**) treatment on H1299 cells migration.

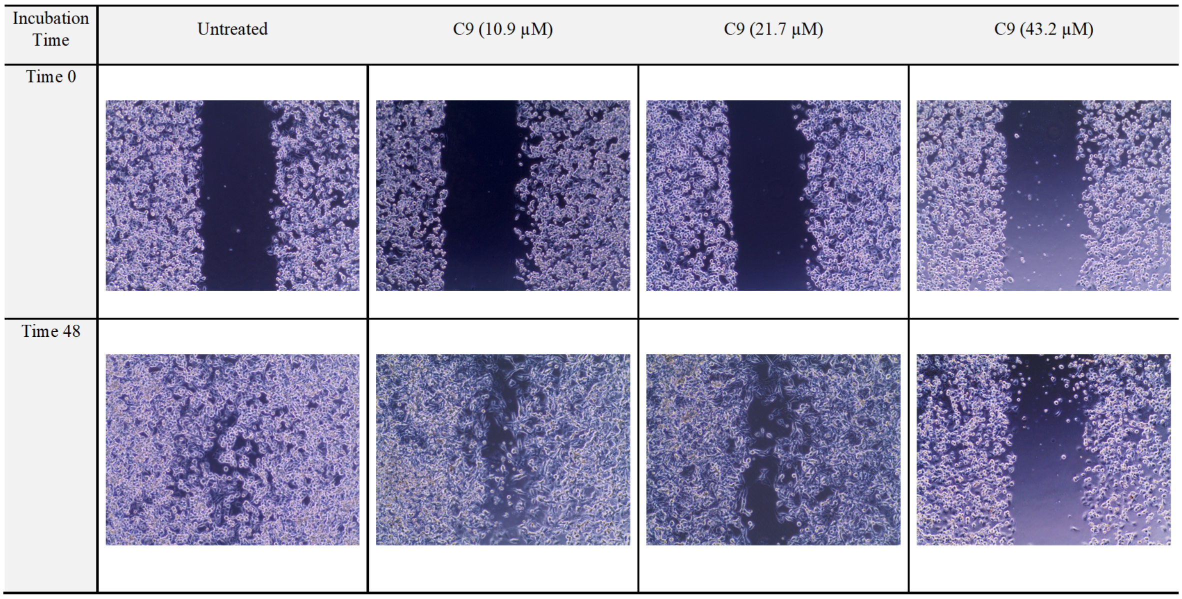

Figure S9. The effect of MMP-12 inhibitor (**C9**) treatment on H1299 cells migration.

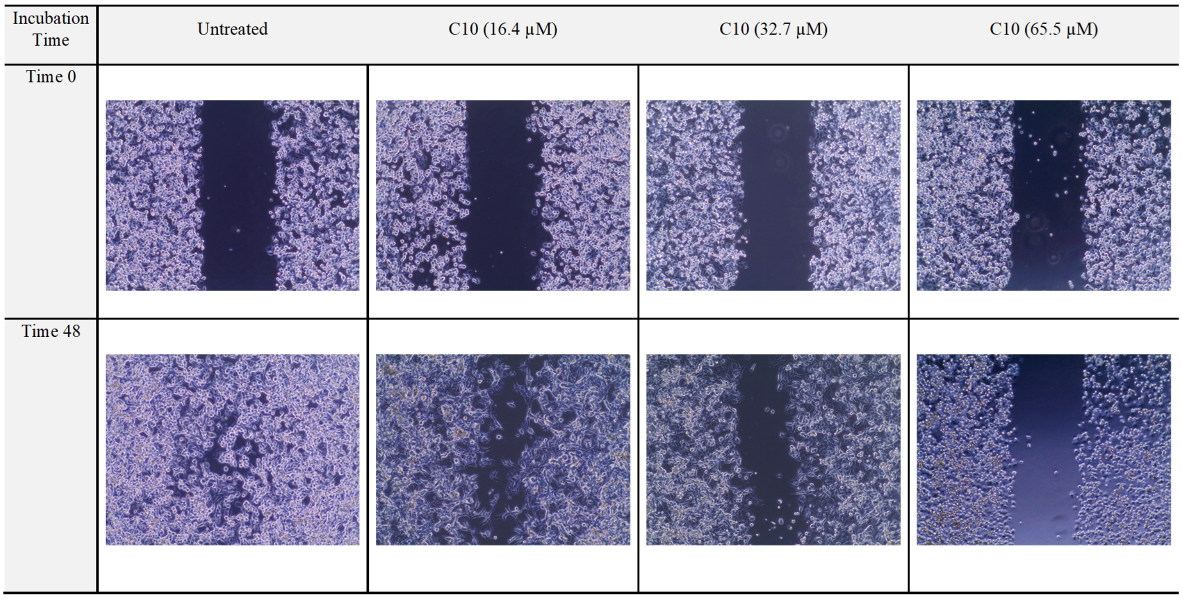

Figure S10. The effect of MMP-12 inhibitor (**C10**) treatment on H1299 cells migration.

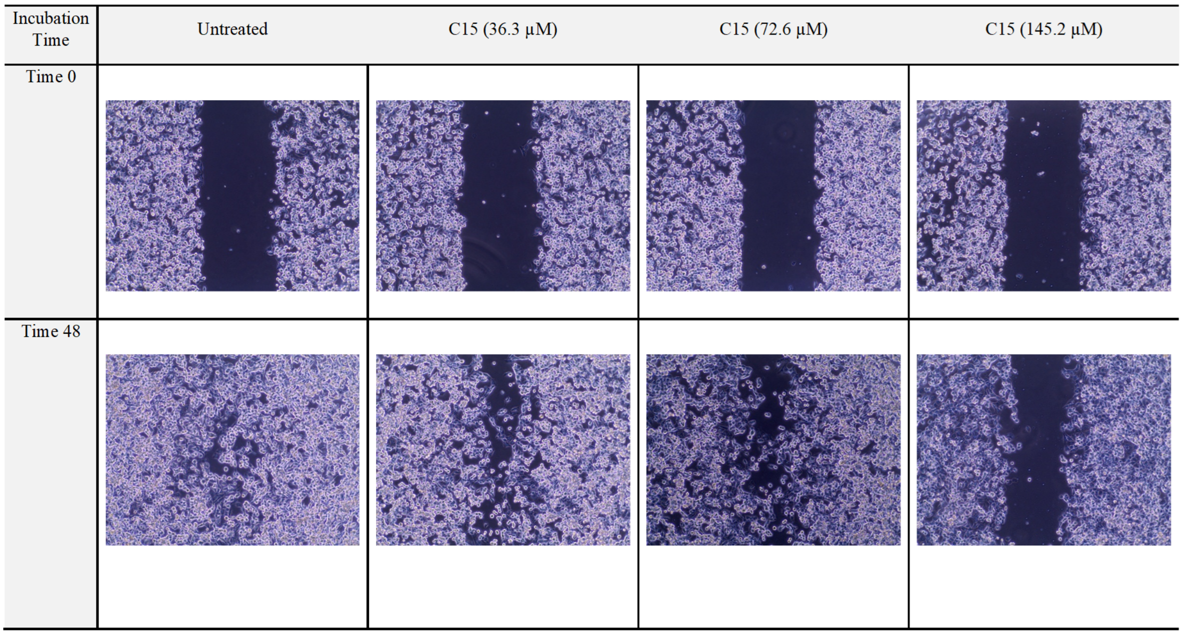

Figure S11. The effect of MMP-12 inhibitor (**C15**) treatment on H1299 cells migration.

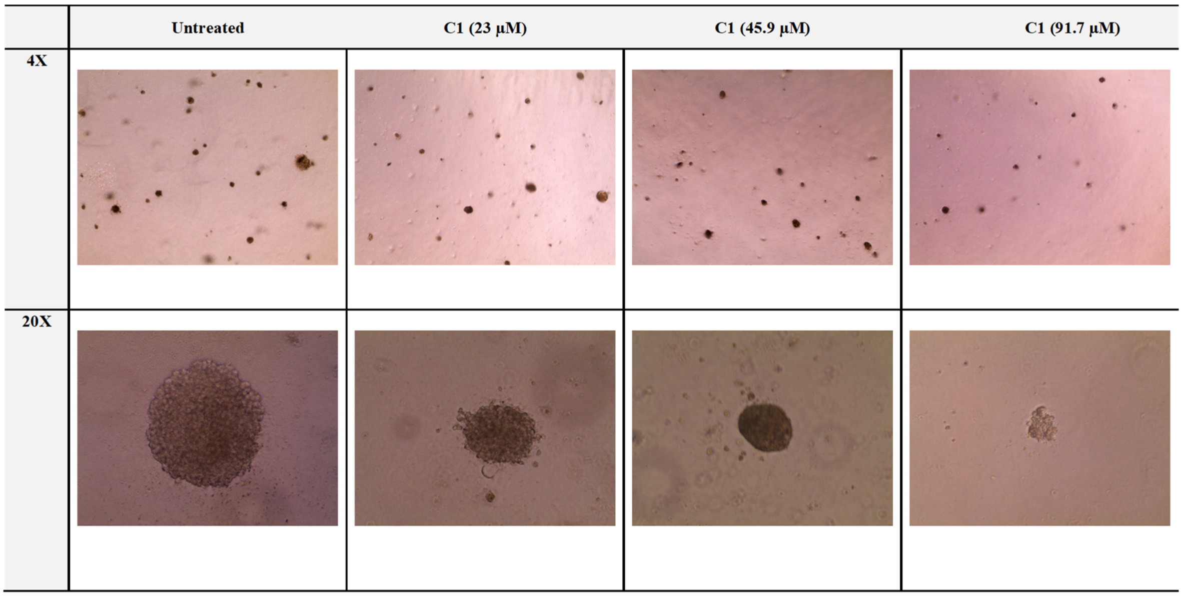

Figure S12. The effect of MMP-12 inhibitor (**C1**) treatment on anchorage independent growth of H1299 lung cancer cell line using colony formation assay. Images show colonies at different magnifications using EVOS XL Core imaging system. µM: micromolar

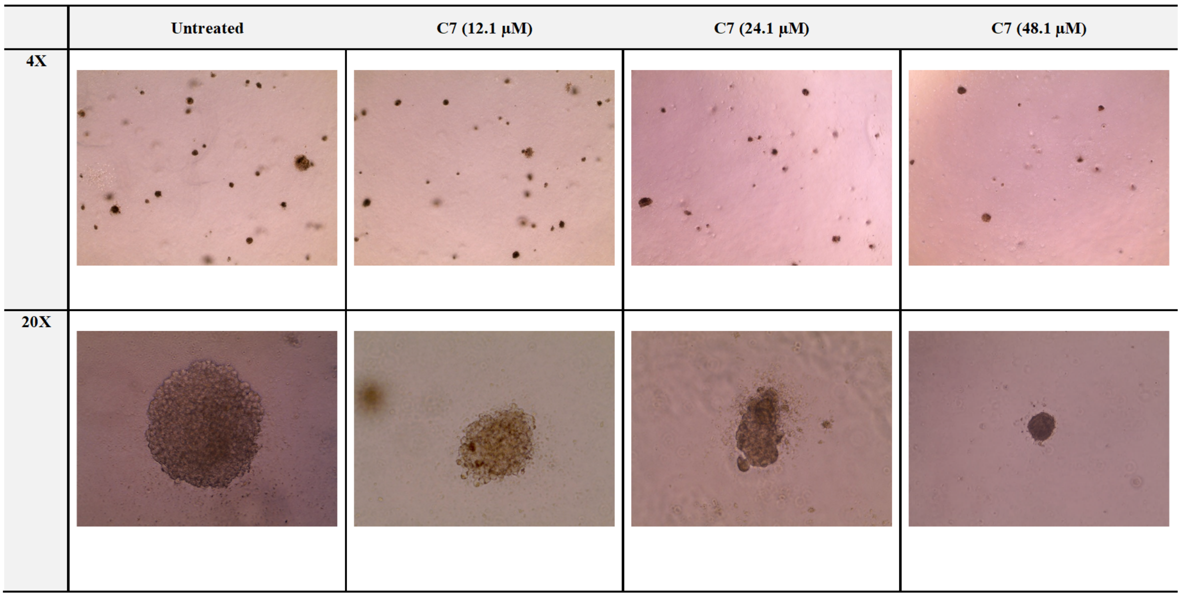

Figure S13. The effect of MMP-12 inhibitor (**C7**) treatment on anchorage independent growth of H1299 lung cancer cell line using colony formation assay. Images show colonies at different magnifications using EVOS XL Core imaging system. µM: micromolar

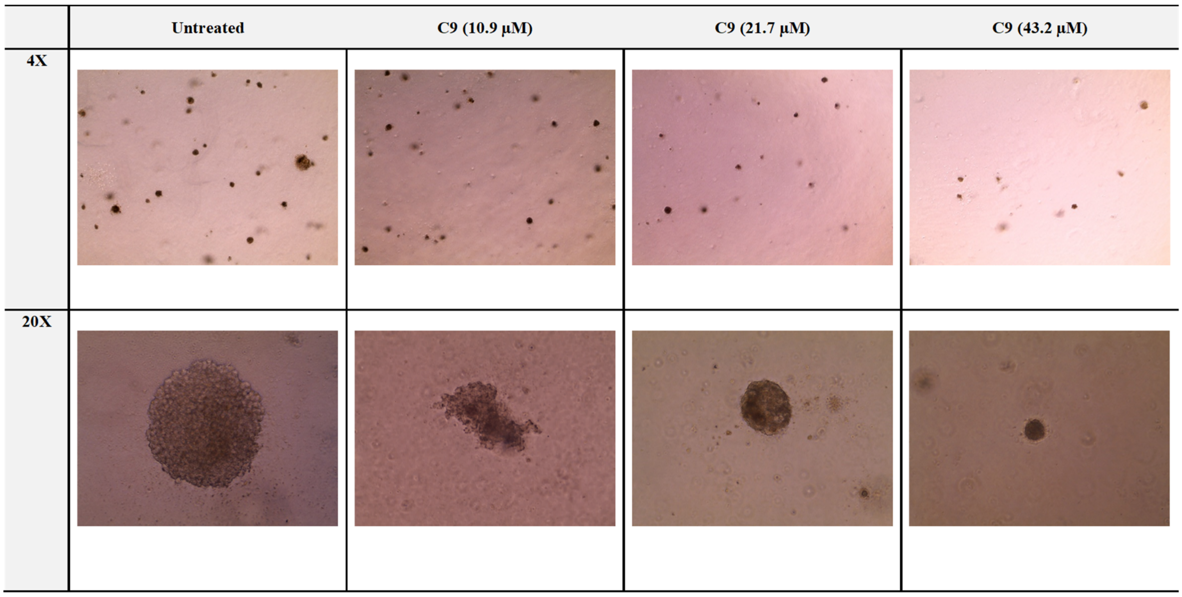

Figure S14. The effect of MMP-12 inhibitor (**C9**) treatment on anchorage independent growth of H1299 lung cancer cell line using colony formation assay. Images show colonies at different magnifications using EVOS XL Core imaging system. µM: micromolar

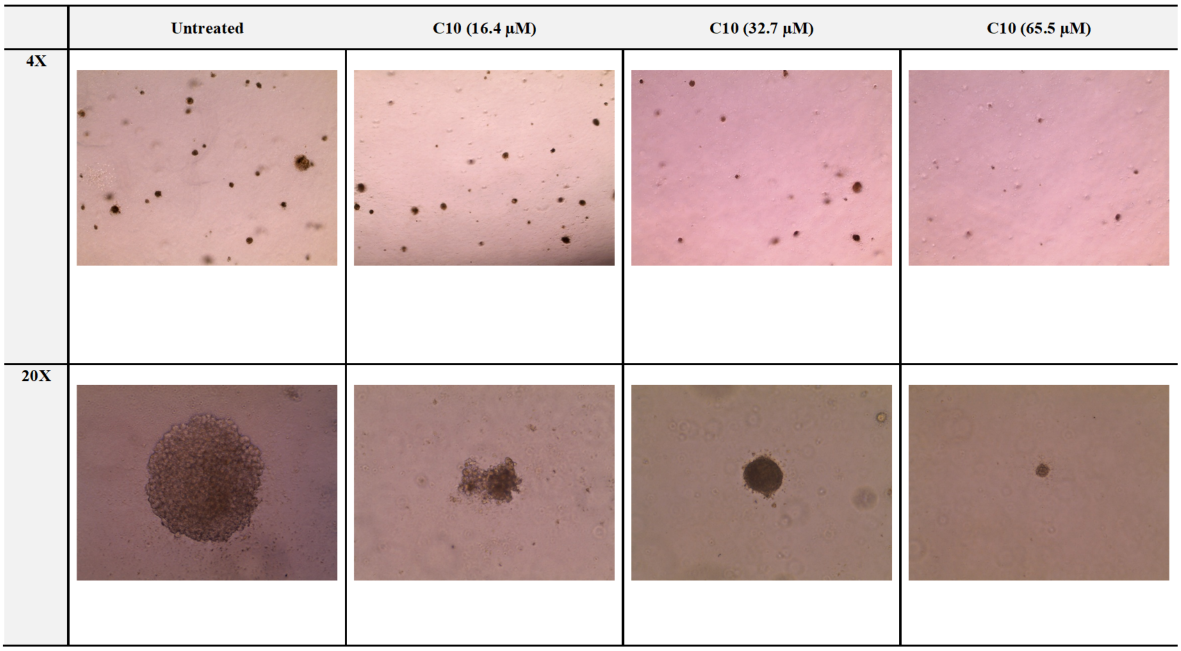

Figure S15. The effect of MMP-12 inhibitor (**C10**) treatment on anchorage independent growth of H1299 lung cancer cell line using colony formation assay. Images show colonies at different magnifications using EVOS XL Core imaging system. µM: micromolar

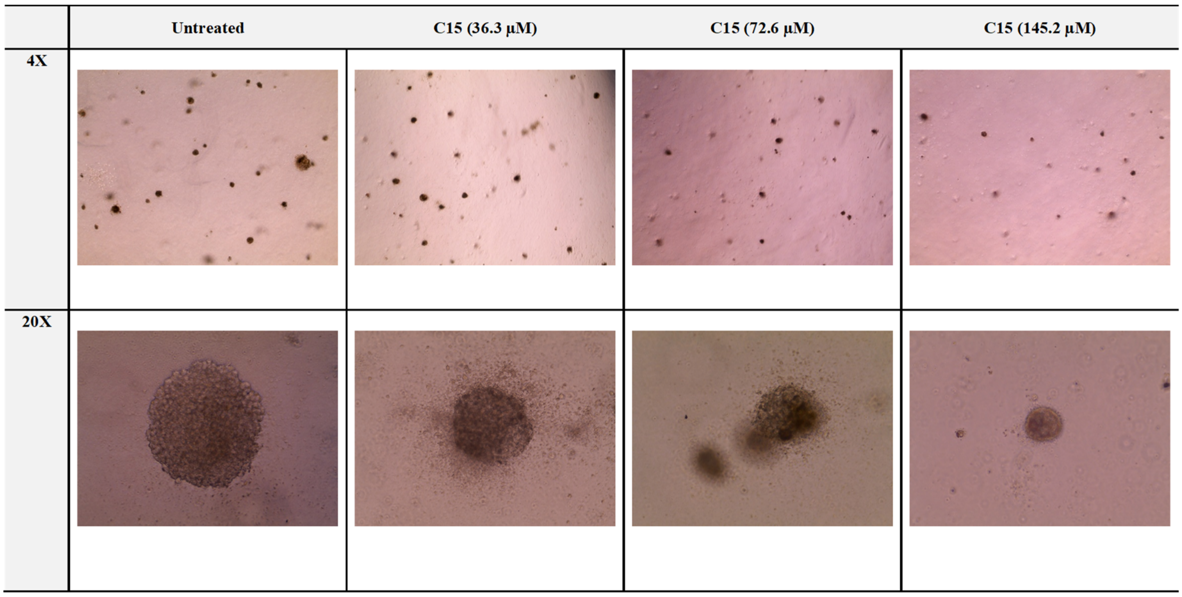

Figure S16. The effect of MMP-12 inhibitor (**C15**) treatment on anchorage independent-growth of H1299 lung cancer cell line using colony formation assay. Images show colonies at different magnifications using EVOS XL Core imaging system. µM: micromolar.

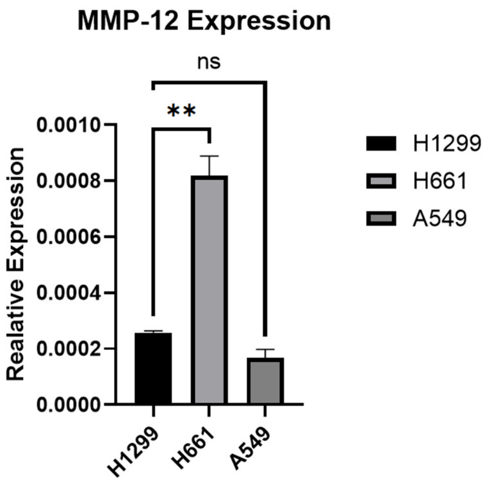

Figure S17. Relative expression levels of MMP-12 in untreated H1299, A549, and H661 lung cancer cell lines.

Baseline MMP-12 expression was quantified in three untreated lung cancer cell lines using the 2^–ΔCt method, with GAPDH as the internal control. Relative expression is presented as mean ± SD from duplicate measurements. A549 exhibited the highest MMP-12 expression, followed by H1299 and H661. Statistical analysis was performed using one-way ANOVA, and significance levels are indicated as follows: ns (not significant) P > 0.05; * P ≤ 0.05; ** P ≤ 0.01; *** P ≤ 0.001; **** P ≤ 0.0001 (GraphPad Prism 8).

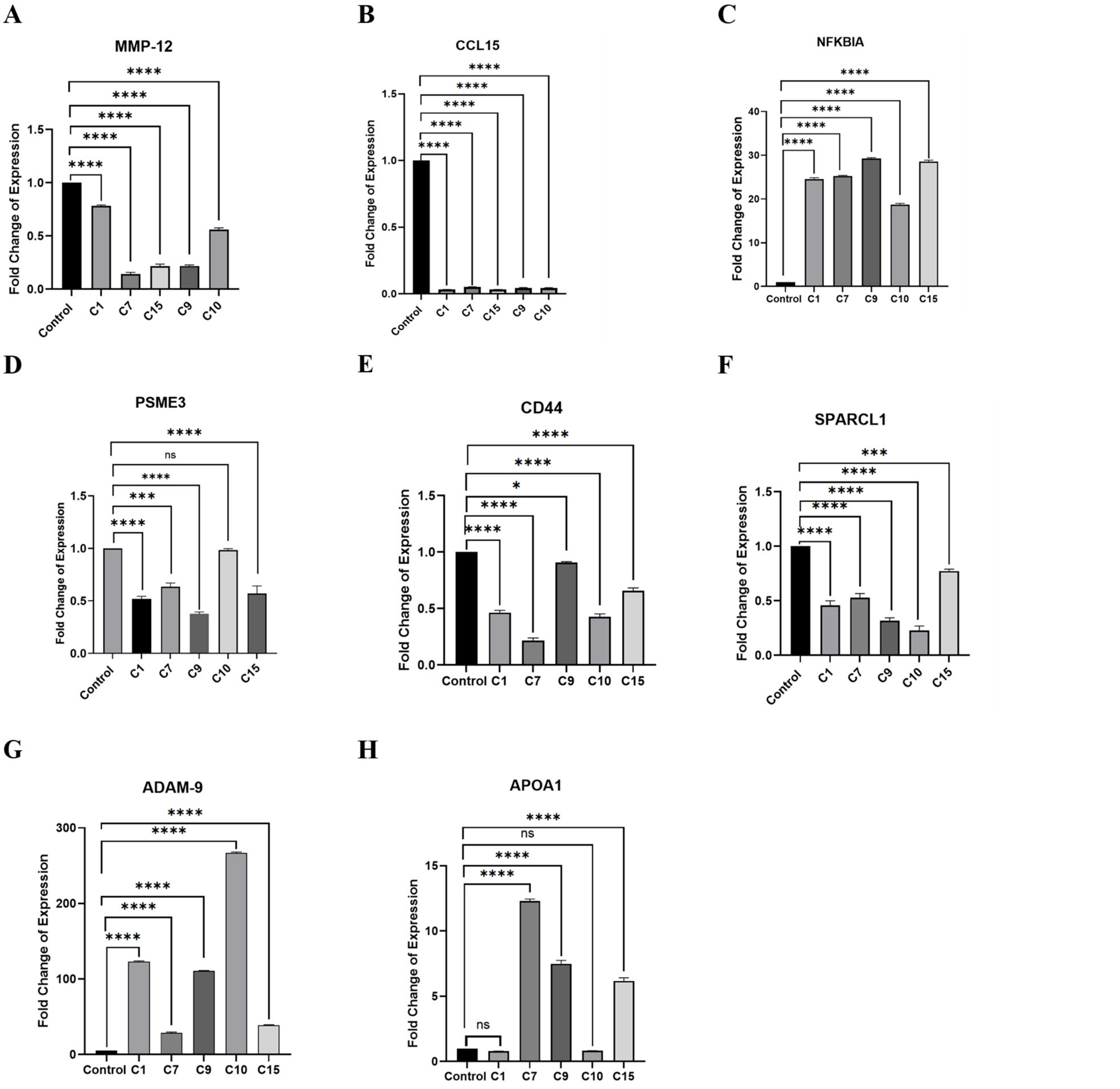

Figure S18. The Effect of 72 hours treatment of MMP-12 inhibitors on RNA expression of the prioritized genes in the H1299 lung cancer cell line in relation to the control, untreated cells. **(A)** The Effect of MMP-12 inhibitors on expression of *MMP-12* gene. **(B)** The Effect of MMP-12 inhibitors on expression of *CCL15* gene. **(C)** The Effect of MMP-12 inhibitors on expression of *NFKBIA* gene. **D)** The Effect of MMP-12 inhibitors on expression of *PSME3* gene. **(E).** The Effect of MMP-12 inhibitors on expression of *CD44* gene. **(F)** The Effect of MMP-12 inhibitors on RNA expression of *SPARCL1* gene. **(G)** The Effect of MMP-12 inhibitors on expression of *ADAM9* gene. **(H)** The Effect of 72 hours treatment of MMP-12 inhibitors on expression of *APOA1* gene.

A

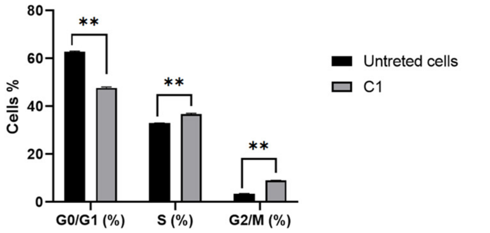

B

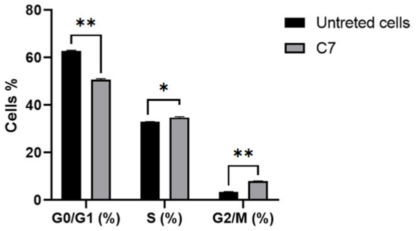

C

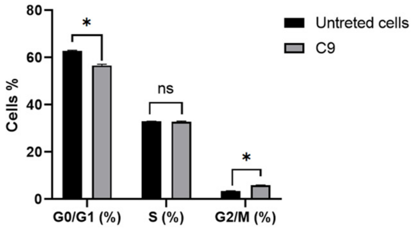

D

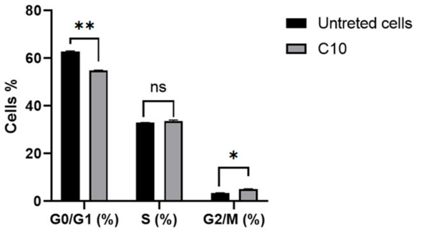

E

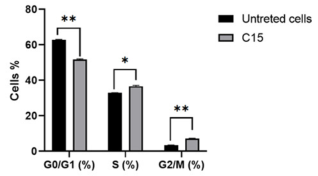

Figure S19. Percentages of cell cycle phases effect of MMP-12 for 48 h (1/2 IC_{50}) on the cell cycle of lung cancer cell lines H1299.

(A) Percentages of cell cycle phases effect of compound **C1.** **(B)** Percentages of cell cycle phases effect of compound **C7.** **(C)** Percentages of cell cycle phases effect of compound **C9.** **(D)** Percentages of cell cycle phases effect of compound **C10** **(E)** Percentages of cell cycle phases effect of compound **C15.**

Percentages represent DNA content upon PI staining of respective samples showing in G0/G1, S, and G2/M phases of cell cycle. All results are expressed as mean + SD.

Fold difference expressed as mean±SD and was measured using ΔΔCt. All experiments were run in duplicates. P-value < 0.05 expresses significantly different from respective untreated cells' status; while asterisk: ns (not-significant) P > 0.05; * P ≤ 0.05; ** P ≤ 0.01; *** P ≤ 0.001; **** P ≤ 0.0001 (according to GraphPad *Prism* 8). h: hour; µM: micromolar.

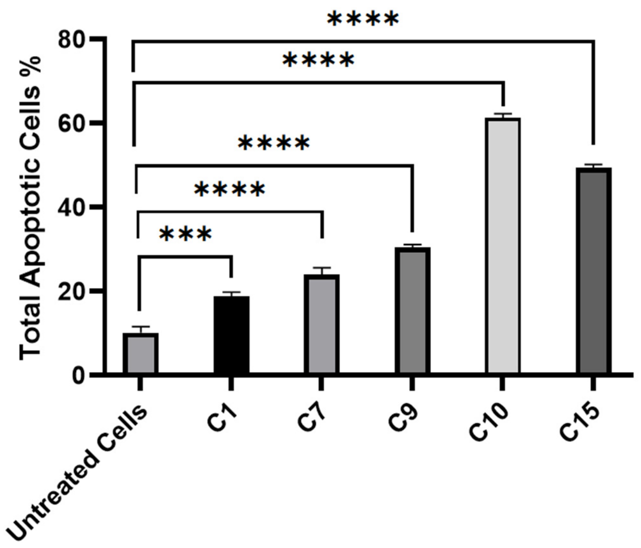

Figure S20. Effect of MMP-12 inhibitors on apoptosis induction in H1299 lung cancer cells.

Total apoptotic cells (%) in H1299 cells treated with the indicated compounds at double IC_{50} for 72 hours, as assessed by Annexin V-FITC/PI flow cytometry. Data are presented as mean ± SD (n = 2). Statistical significance was determined using one-way ANOVA with Tukey's post hoc test (***p < 0.001, ****p < 0.0001).

Table S1. Full Downstream targets of MMP-12 extracted from Metacore™ interaction analysis.

Downstream Network Object	Gene Symbol*	Effect of MMP-12	Mechanism
ACTR3	*ACTR3*	Unspecified	Cleavage
ADAM9	*ADAM9*	Unspecified	Cleavage
Aha1	*AHSA1*	Unspecified	Cleavage
AL7A1	*ALDH7A1*	Unspecified	Cleavage
Alpha crystallin B	*CRYAB*	Unspecified	Cleavage
Alpha-actinin 1	*ACTN1*	Unspecified	Cleavage
Alpha-actinin 4	*ACTN4*	Unspecified	Cleavage
Alpha-parvin	*PARVA*	Unspecified	Cleavage
Annexin I	*ANXA1*	Unspecified	Cleavage
AP3D1	*AP3D1*	Unspecified	Cleavage
ARCN1	*ARCN1*	Unspecified	Cleavage
ARP2	*ACTR2*	Unspecified	Cleavage
ARPC1B	*ARPC1B*	Unspecified	Cleavage
Ataxin-10	*ATXN10*	Unspecified	Cleavage

ATIC	*ATIC*	Unspecified	Cleavage
Beta-catenin	*CBY1*	Unspecified	Cleavage
Biglycan	*BGN*	Unspecified	Cleavage
Caldesmon	*CALD1*	Unspecified	Cleavage
Calponin-2	*CNN2*	Unspecified	Cleavage
Calponin-3	*CNN3*	Unspecified	Cleavage
Calreticulin	*CALR*	Unspecified	Cleavage
Calumenin	*CALU*	Unspecified	Cleavage
CAPZA1	*CAPZA1*	Unspecified	Cleavage
CAPZA2	*CAPZA2*	Unspecified	Cleavage
Carboxypeptidase H	*CPE*	Unspecified	Cleavage
CCL1	*CCL1*	Unspecified	Cleavage
CCL14	*CCL14*	Unspecified	Cleavage
CD44	*CD44*	Unspecified	Cleavage
c-Kit	*KIT*	Unspecified	Cleavage
Clusterin	*CLU*	Unspecified	Cleavage
Collagen II	*COL2A1*	Unspecified	Cleavage
Collagen XII	*COL12A1*	Unspecified	Cleavage
COPE	*COPE*	Unspecified	Cleavage
CORO1C	*CORO1C*	Unspecified	Cleavage
Cytochrome B5	*CYB5*	Unspecified	Cleavage
DRG1	*DRG1*	Unspecified	Cleavage
Dystroglycan	*DAG1*	Unspecified	Cleavage
eIF2S1	*EIF2S1*	Unspecified	Cleavage
EIF3EIP	*EIF3L*	Unspecified	Cleavage
eIF3S5	*EIF3F*	Unspecified	Cleavage
ENO2	*ENO2*	Unspecified	Cleavage
Eotaxin	*CCL11*	Unspecified	Cleavage
Ephrin-A5	*EFNA5*	Unspecified	Cleavage
ERdj5	*DNAJC10*	Unspecified	Cleavage

Fibrillin 1	*FBN1*	Unspecified	Cleavage
Fibrillin 2	*FBN2*	Unspecified	Cleavage
Fibrinogen	*FGA*	Unspecified	Cleavage
Fibromodulin	*FMOD*	Unspecified	Cleavage
Galectin-3	*LGALS3*	Unspecified	Cleavage
GCP2	*GOLGA4*	Unspecified	Cleavage
GRO-2	*CXCL2*	Unspecified	Cleavage
GRO-3	*CXCL3*	Unspecified	Cleavage
GSK3 beta	*GSK3B*	Unspecified	Cleavage
HDGF2	*HDGFL2*	Unspecified	Cleavage
HMGA2	*HMGA2*	Unspecified	Cleavage
hnRNP C	*HNRNPC*	Unspecified	Binding
HSP90 alpha	*HSP90A*	Unspecified	Cleavage
IP10	*CXCL10*	Unspecified	Cleavage
I-TAC	*CXCL11*	Unspecified	Cleavage
Karyopherin beta 1	*KPNB1*	Unspecified	Cleavage
LAMA5	*LAMA5*	Unspecified	Cleavage
MIG	*MIG*	Unspecified	Cleavage
MMP-15	*MMP15*	Unspecified	Binding
Plasminogen	*PLG*	Unspecified	Cleavage
PPase (inorganic)	*PPA1*	Unspecified	Cleavage
PPBP	*PPBP*	Unspecified	Cleavage
PRG4	*PRG4*	Unspecified	Cleavage
Prolargin	*PRELP*	Unspecified	Cleavage
Ran	*RAN*	Unspecified	Cleavage
RARS	*RARS1*	Unspecified	Cleavage
Septin 7	*SEPTIN7*	Unspecified	Cleavage
SERA	*PHGDH*	Unspecified	Cleavage
TMEM66	*SARAF*	Unspecified	Binding

*Genes names are as per the HGNC approved nomenclature (HUGO Gene Nomenclature Committee)

Table S2. Primers' forward and reverse sequences with their optimized annealing temperature.

Primer	Primer sequence	Ta (°C)
**MMP12-hsa**	• **Forward:** 5′-GATGCTGTCACTACCGTGGGAA-3′<br>• **Reverse:** 5′-CAATGCCAGATGGCAAGGTTGG-3′	60 °C
**NFKIBIA**	• **Forward**: 5'-ACCTGGTGTCACTCCTGTTGA-3'<br>• **Reverse**: 5'-CTGCTGCTGTATCCGGGTG-3'	60 °C
**PSME3**	• **Forward**: 5'-CCC TCA TGT GGA GGA CTA CC-3'<br>• **Reverse**: 5'-TGT CAC ATA TTG ATT CCT CAG C-3'	60 °C
**SPARC1**	• **Forward:** 5'-CAACTGCTGAAACGGTAGCA-3'<br>• **Reverse:** 5'-GAACTCTTGCCCTGTTCTGC-3'	60 °C
**CD44**	• **Forward**: 5′-GAC ACATAT TGC TTC AAT GCT TCA-3′<br>• **Reverse:** 5′-GAT GCC AAGATG ATC AGC CAT TCT G-3′	62 °C
**ADAM-9**	• **Forward:** 5′-CCTCGGGGACCCTTCGTGT-3′<br>• **Reverse:** 5′-ATCCCATAACTCGCATTCTCTAAA-3′	56 °C
**CCL15**	• **Forward:** 5′-CTGACTGCTGCACCTCCTACATC-3′<br>• **Reverse:** 5′-GACCACTGGGTTTGGCACAGAC-3′	60 °C
**APOA1**	• **Forward:** 5′-CTAAAGCTCCTTGACAACTGGG-3′<br>• **Reverse:** 5′- TTTCCAGGTTATCCCAGAACTC-3′	60 °C

Table S3. Chemical Structures of Amine Derivatives (a-f)

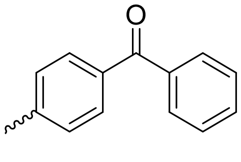 a	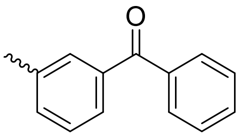 b	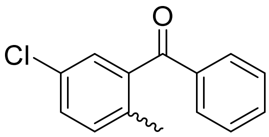 c
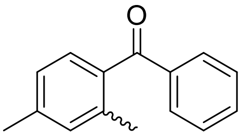 d	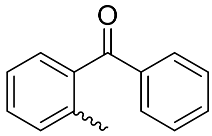 e	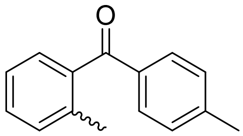 f

Table S4. The IC_{50} values for synthesized and purchased MMP-12 inhibitors against A549, H1299 and H661 lung cancer cells.

Cell Line	A549	H1299	H661
**Incubation Time (hours)**		**C1 IC_{50} (μM)**	
**48 Hours**	197.6	358.9	60.4
**72 Hours**	118.8	91.7	33.9
**96 Hours**	81.95	99.7	129.0
**Incubation Time (hours)**		**C2 IC_{50} (μM)**	
**48 Hours**	>1000	827.1	598.6
**72 Hours**	>1000	692.4	410.4
**96 Hours**	>1000	349.1	350.5
**Incubation Time (hours)**		**C3 IC_{50} (μM)**	
**48 Hours**	251.0	557.1	570.6
**72 Hours**	268.9	401.0	300.8
**96 Hours**	999.2	338.0	202.9
**Incubation Time (hours)**		**C4 IC_{50} (μM)**	
**48 Hours**	963.0	615.9	562.1
**72 Hours**	395.8	302.1	318.9
**96 Hours**	168.5	253.9	145.6
**Incubation Time (hours)**		**C5 IC_{50} (μM)**	
**48 Hours**	>1000	635.8	488.9

**72 Hours**	>1000	448.7	365.8
**96 Hours**	865.0	419.4	225.6
**Incubation Time (hours)**		**C6 IC_{50} (µM)**	
**48 Hours**	>1000	360.6	447.8
**72 Hours**	>1000	267.9	259.9
**96 Hours**	829.5	256.2	270.4
**Incubation Time (hours)**		**C7 IC_{50} (µM)**	
**48 Hours**	320.0	197.5	43.4
**72 Hours**	308.2	48.11	52.4
**96 Hours**	234.8	71.83	82.8
**Incubation Time (hours)**		**C8 IC_{50} (µM)**	
**48 Hours**	>1000	803.3	573.7
**72 Hours**	>1000	349.7	388.3
**96 Hours**	>1000	302.1	662.0
**Incubation Time (hours)**		**C9 IC_{50} (µM)**	
**48 Hours**	427.9	496.3	84.4
**72 Hours**	99.7	43.4	51.3
**96 Hours**	35.69	66.8	51.0
**Incubation Time (hours)**		**C10 IC_{50} (µM)**	
**48 Hours**	335.4	622.5	62.4
**72 Hours**	101.2	65.5	55.8
**96 Hours**	52.1	65.3	64.9
**Incubation Time (hours)**		**C11 IC_{50} (µM)**	
**48 Hours**	>1000	412.5	145.4
**72 Hours**	339	340.5	379.7
**96 Hours**	236.8	513	>1000
**Incubation Time (hours)**		**C12 IC_{50} (µM)**	
**48 Hours**	>1000	773.4	467.1
**72 Hours**	>1000	437.2	618.0
**96 Hours**	>1000	>1000	>1000
**Incubation Time (hours)**		**C13 IC_{50} (µM)**	
**48 Hours**	>1000	433.0	665.1
**72 Hours**	>1000	>1000	619.2
**96 Hours**	961.7	>1000	481.6
**Incubation Time (hours)**		**C14 IC_{50} (µM)**	
**48 Hours**	732.0	722.1	>1000
**72 Hours**	725.0	565.6	595.2
**96 Hours**	294.3	254.3	205.8
**Incubation Time (hours)**		**C15 IC_{50} (µM)**	
**48 Hours**	500	222.2	79.97

**72 Hours**	312.1	145.2	130.6
**96 Hours**	132.4	178.6	128.4
**Incubation Time (hours)**	**C16 IC_{50} (μM)**		
**48 Hours**	>1000	>1000	>1000
**72 Hours**	>1000	>1000	257.2
**96 Hours**	461.3	492.4	>1000
**Incubation Time (hours)**	**C17 IC_{50} (μM)**		
**48 Hours**	>1000	>1000	>1000
**72 Hours**	>1000	>1000	426.3
**96 Hours**	>1000	273.6	>1000
**Incubation Time (hours)**	**C18 IC_{50} (μM)**		
**48 Hours**	>1000	>1000	875.1
**72 Hours**	>1000	>1000	>1000
**96 Hours**	>1000	514.2	>1000
**Incubation Time (hours)**	**C19 IC_{50} (μM)**		
**48 Hours**	>1000	984.4	829.7
**72 Hours**	>1000	583.8	513.3
**96 Hours**	>1000	>1000	>1000
**Incubation Time (hours)**	**C20 IC_{50} (μM)**		
**48 Hours**	>1000	>1000	>1000
**72 Hours**	266.7	852.9	>1000
**96 Hours**	219.9	696.3	333.8
**Incubation Time (hours)**	**C21 IC_{50} (μM)**		
**48 Hours**	>1000	966.8	>1000
**72 Hours**	>1000	594.1	>1000
**96 Hours**	>1000	337.1	510.8
**Incubation Time (hours)**	**C22 IC_{50} (μM)**		
**48 Hours**	>1000	964.1	406.2
**72 Hours**	>1000	542.8	327.3
**96 Hours**	>1000	729.8	444.8
**Incubation Time (hours)**	**C23 IC_{50} (μM)**		
**48 Hours**	>1000	>1000	533.3
**72 Hours**	>1000	>1000	448.7
**96 Hours**	726.0	>1000	694.9
**Incubation Time (hours)**	**C24 IC_{50} (μM)**		
**48 Hours**	>1000	>1000	>1000
**72 Hours**	>1000	>1000	>1000
**96 Hours**	>1000	>1000	359.7
**Incubation Time (hours)**	**C25 IC_{50} (μM)**		
**48 Hours**	>1000	>1000	457.3

**72 Hours**	>1000	>1000	304.5
**96 Hours**	>1000	>1000	>1000
**Incubation Time (hours)**	**C26 IC_{50} (µM)**		
**48 Hours**	>1000	481.3	>1000
**72 Hours**	>1000	489.8	>1000
**96 Hours**	>1000	>1000	>1000
**Incubation Time (hours)**	**C27 IC_{50} (µM)**		
**48 Hours**	>1000	607.0	588.9
**72 Hours**	>1000	554.0	514.0
**96 Hours**	>1000	>1000	485.8
**Incubation Time (hours)**	**H1 IC_{50} (µM)**		
**48 Hours**	>1000	>1000	988.9
**72 Hours**	>1000	672.2	935.8
**96 Hours**	>1000	>1000	>1000
**Incubation Time (hours)**	**H2 IC_{50} (µM)**		
**48 Hours**	>1000	>1000	927.5
**72 Hours**	>1000	>1000	726.7
**96 Hours**	>1000	744.3	998.4
**Incubation Time (hours)**	**H3 IC_{50} (µM)**		
**48 Hours**	>1000	492.1	>1000
**72 Hours**	>1000	382.1	>1000
**96 Hours**	>1000	>1000	552.0
**Incubation Time (hours)**	**H4 IC_{50} (µM)**		
**48 Hours**	>1000	696.1	939.3
**72 Hours**	>1000	251.4	777.8
**96 Hours**	>1000	392.6	162.0

Experiments were carried out for 48, 72, and 96 hours of treatment duration. Experiments were run in duplicate for at least three independent trials (n=6). Standard deviation did not exceed 10%. µM: micromolar.

Table S5. The effect of MMP-12 inhibitors treatment on H1299 cells migration.

Compound	Concentration (µM)	Wound closure %	*p*-value	Significance	Wound migration rate (px/h)
Untreated	-	99	-	-	16,702 px/h
C1	91.7	10	≤ 0.0001	Significant	1,640.73 px/h
C1	45.9	50	≤ 0.0001	Significant	9,225 px/h
C1	23	72	≤ 0.0001	Significant	11,751 px/h
C7	48.1	46	≤ 0.0001	Significant	7,358 px/h
C7	24.1	48	≤ 0.0001	Significant	7,770 px/h
C7	12.1	77	≤ 0.001	Significant	12,336 px/h
C9	43.2	20	≤ 0.0001	Significant	3,684.85 px/h

**C9**	**21.7**	**24**	**≤ 0.0001**	Significant	**13,078.10 px/h**
**C9**	**10.9**	**89**	**≤ 0.01**	Significant	**16,141.02 px/h**
**C10**	**65.5**	**6**	**≤ 0.0001**	Significant	**964 px/h**
**C10**	**32.7**	**40**	**≤ 0.0001**	Significant	**6,665 px/h**
**C10**	**16.4**	**67**	**≤ 0.0001**	Significant	**10,983.31 px/h**
**C15**	**145.2**	**33**	**≤ 0.0001**	Significant	**5,907 px/h**
**C15**	**72.6**	**85**	**≤ 0.01**	Significant	**14,790 px/h**
**C15**	**36.3**	**91**	**> 0.05**	ns	**15,449 px/h**

P-value < 0.05 express significantly different from respective untreated conditions; while asterisk: ns (not-significant) P > 0.05; * P ≤ 0.05; ** P ≤ 0.01; *** P ≤ 0.001; **** P ≤ 0.0001 (according to GraphPad prism 9). µM: micromolar.

Table S6. Effect of **C1**, **C7**, **C9**, **C10**, and **C15**, on colony size and count of lung cancer cell line (H1299) using colony formation assay.

**Compound**	**Concentration (µM)**	**Colony count**	***p*-value**	**Significance**	**Colony average size (px)**	***p*-value**	**Significance**
**Untreated**	**-**	**920**	**-**	-	**746758**		
**C1**	**91.7**	**292**	**≤ 0.0001**	Significant	**51655**	**≤ 0.0001**	Significant
**C1**	**45.9**	**408**	**≤ 0.0001**	Significant	**130041**	**≤ 0.0001**	Significant
**C1**	**23**	**520**	**≤ 0.0001**	Significant	**262500**	**≤ 0.0001**	Significant
**C7**	**48.1**	**252**	**≤ 0.0001**	Significant	**36831**	**≤ 0.0001**	Significant
**C7**	**24.1**	**436**	**≤ 0.0001**	Significant	**206454**	**≤ 0.0001**	Significant
**C7**	**12.1**	**608**	**≤ 0.0001**	Significant	**303115**	**≤ 0.0001**	Significant
**C9**	**65.5**	**142**	**≤ 0.0001**	Significant	**9195**	**≤ 0.0001**	Significant
**C9**	**32.7**	**262**	**≤ 0.0001**	Significant	**77102**	**≤ 0.0001**	Significant
**C9**	**16.4**	**632**	**≤ 0.0001**	Significant	**268382**	**≤ 0.0001**	Significant
**C10**	**43.2**	**152**	**≤ 0.0001**	Significant	**21796**	**≤ 0.0001**	Significant
**C10**	**21.7**	**352**	**≤ 0.0001**	Significant	**141917**	**≤ 0.0001**	Significant
**C10**	**10.9**	**504**	**≤ 0.0001**	Significant	**230732**	**≤ 0.0001**	Significant
**C15**	**145.2**	**295**	**≤ 0.0001**	Significant	**66131**	**≤ 0.0001**	Significant
**C15**	**72.6**	**428**	**≤ 0.0001**	Significant	**217288**	**≤ 0.0001**	Significant
**C15**	**36.3**	**656**	**≤ 0.0001**	Significant	**359402**	**≤ 0.0001**	Significant

Colony size was measured using particle analysis upon identifying colony colour threshold through ImageJ software (Ver. 1.53e.). P-value <0.05 indicates statistical significance in comparison to untreated control, while asterisk: ns (not-significant) P > 0.05; * P ≤ 0.05; ** P ≤ 0.01; *** P ≤ 0.001; **** P ≤ 0.000l (according to GraphPad prism 9). uM: micromolar

Table S7. Western blot analysis of MMP-12 (Normalized to GAPDH) in H1299 lung cancer cell line treated with compounds **C15**, **C9**, and **C10** at ¼ and ½ IC_{50}.

	**C10** 1/2 IC_{50}	**C10** 1/4 IC_{50}	**C9** 1/2 IC_{50}	**C9** 1/4 IC_{50}	**C15** 1/2 IC_{50}	**C15** 1/4 IC_{50}
**GAPDH**	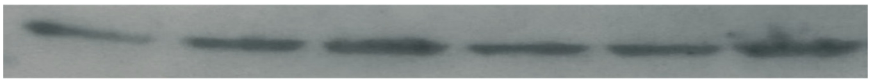					
	**C15** 1/2 IC_{50}	**C15** 1/4 IC_{50}	**C9** 1/2 IC_{50}	**C9** 1/4 IC_{50}	**C10** 1/2 IC_{50}	**C10** 1/4 IC_{50}
**MMP-12**	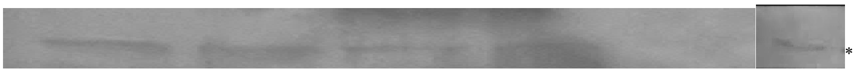					
	**C15** ½ IC_{50}	**C15** ¼ IC_{50}	**C9** ½ IC_{50}	**C9** ¼ IC_{50}	**C10** ½ IC_{50}	**C10** ¼ IC_{50}
Normalized MMP-12	0.21080	0.12695	0.16278	0.22239	No Band	0.13214

* The **C10** (¼ IC_{50}) sample was run on a separate gel processed in parallel with the other treatments under identical electrophoresis and blotting conditions. Normalization to GAPDH was applied uniformly across all samples

Table S8 Western blot analysis of MMP-12 (normalized to GAPDH) in H1299 lung cancer cell line treated with compounds **C1** and **C7** at ¼ and ½ IC_{50}.

	**C1** 1/2 IC_{50}	**C1** 1/4 IC_{50}	**C7** 1/2 IC_{50}	**C7** 1/4 IC_{50}
GAPDH	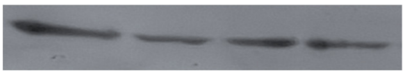			
MMP-12	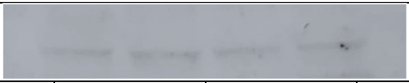			
Normalized MMP-12	0.1966	0.3747	0.2924	0.3510

Table S9. Western blot analysis of MMP-12 (normalized to GAPDH) in H1299 cells treated with compounds **C1**, **C7**, **C9**, **C10**, and **C15** at 0.1 IC_{50}, with Untreated Controls from H1299, A549, and H661.

	**C1** 0.1 IC_{50}	**C7** 0.1 IC_{50}	**C15** 0.1 IC_{50}	**C9** 0.1 IC_{50}	**C10** 0.1 IC_{50}	**H1299**	**H661**	**A549**
GAPDH	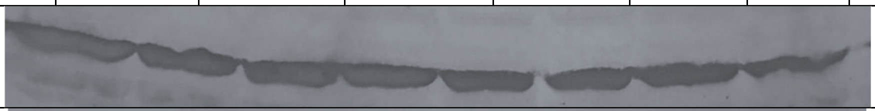							
MMP-12	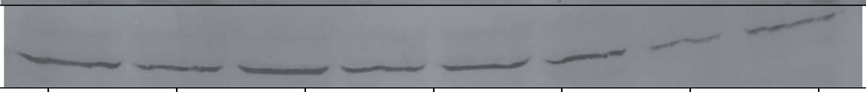							
Normalized MMP-12	0.6296	0.7599	0.8647	0.8134	0.8426	0.9696	0.6882	0.9011

Table S10 Effect of MMP-12 inhibitors on apoptosis induction in H1299 cells as measured by Annexin V/PI staining.

Sample	Viable (%)	Early Apoptosis (%)	Late Apoptosis (%)	Necrosis (%)	Total Apoptosis (%)
**Control**	88.1	8.7	2.6	0.7	11.3
**Cisplatin**	58.1	28.6	11.6	1.7	40.2
**C1**	78.6	16.6	2.9	1.9	19.5
**C7**	73.7	18.5	6.7	1.2	25.2
**C9**	69	25	5.1	0.9	30.1
**C10**	38.9	55.2	5.5	0.4	60.7
**C15**	49.8	43.1	5.8	1.3	48.9

Cells were treated with test compounds at their double IC_{50} concentrations for 72 hours, stained with Annexin V-FITC and propidium iodide (PI), and analyzed via flow cytometry. The table summarizes the percentages of viable, early apoptotic, late apoptotic, and necrotic cells. Cisplatin was used as a positive control.
